# The Increase in Hemoglobin Concentration With Altitude Differs Between World Regions and Is Less in Children Than in Adults

**DOI:** 10.1097/HS9.0000000000000854

**Published:** 2023-04-05

**Authors:** Heimo Mairbäurl, Samuel Kilian, Svenja Seide, Martina U. Muckenthaler, Max Gassmann, Rukundo K. Benedict

**Affiliations:** 1 Translational Pneumology, University Hospital Heidelberg, Germany; 2 Translational Lung Research Center Heidelberg (TLRC), Member of the German Center for Lung Research, Heidelberg, Germany; 3 Institute of Medical Biometry and Informatics (IMBI), University of Heidelberg, Germany; 4 Pediatric Oncology, Hematology & Immunology, University Hospital Heidelberg, Germany; 5 Institute of Veterinary Physiology, Vetsuisse Faculty, and Zurich Center for Integrative Human Physiology (ZIHP), University of Zürich, Switzerland; 6 Universidad Peruana Cayetano Heredia (UPCH), Lima, Peru; 7 ICF, Demographic and Health Surveys (DHS), Rockville, MD, USA

## Abstract

To compensate for decreased oxygen partial pressure, high-altitude residents increase hemoglobin concentrations [Hb]. The elevation varies between world regions, posing problems in defining cutoff values for anemia or polycythemia. The currently used altitude adjustments (World Health Organization [WHO]), however, do not account for regional differences. Data from The Demographic and Health Survey (DHS) Program were analyzed from 32 countries harboring >4% of residents at altitudes above 1000 m. [Hb]-increase, (ΔHb/km altitude) was calculated by linear regression analysis. Tables show 95% reference intervals (RIs) for different altitude ranges, world regions, and age groups. The prevalence of anemia and polycythemia was calculated using regressions in comparison to WHO adjustments. The most pronounced Δ[Hb]/km was found in East Africans and South Americans while [Hb] increased least in South/South-East Asia. In African regions and Middle East, [Hb] was decreased in some altitude regions showing inconsistent changes in different age groups. Of note, in all regions, the Δ[Hb]/km was lower in children than in adults, and in the Middle East, it was even negative. Overall, the Δ[Hb]/km from our analysis differed from the region-independent adjustments currently suggested by the WHO resulting in a lower anemia prevalence at very high altitudes. The distinct patterns of Δ[Hb] with altitude in residents from different world regions imply that one single, region-independent correction factor for altitude is not be applicable for diagnosing abnormal [Hb]. Therefore, we provide regression coefficients and reference-tables that are specific for world regions and altitude ranges to improve diagnosing abnormal [Hb].

## Graphical abstract

**Figure:**
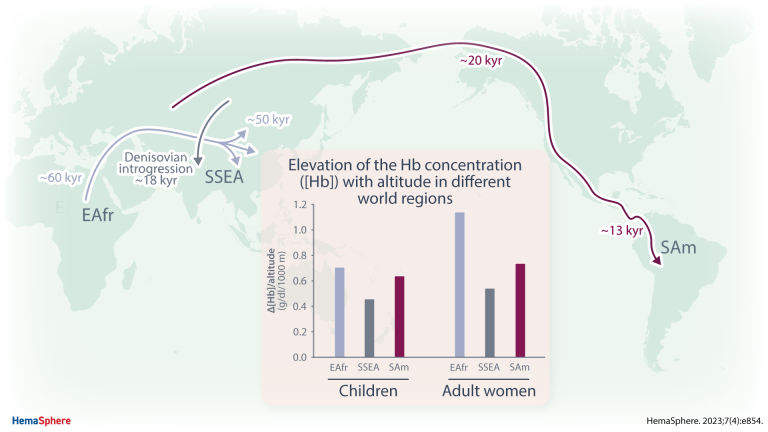

## INTRODUCTION

The hemoglobin concentrations [Hb] of high-altitude residents increases with altitude.^1,2^ Approximately 5% of the world’s population lives at altitudes above 1500 m,^3^ and thus is at risk to suffer from anemia because highland residents rely on adequate iron and vitamin intake^4^ to foster erythropoiesis for maintaining this chronically elevated [Hb]. In turn, anemia adversely affects growth and development, and impairs physical performance. Moreover, abnormally high [Hb] is often related to chronic mountain sickness.^5^

Increasing [Hb] is an effective means to maintain a normal arterial blood O_2_ content despite decreased oxygen partial pressure (PaO_2_) in inspiratory air at high altitude that otherwise compromises tissue oxygen supply by decreasing arterial oxygen saturation (SaO_2_).^6^ Mechanisms of adjustment are best observed in lowlanders ascending to high altitude, where the increase in [Hb] is achieved by a decrease in plasma volume^7^ and by stimulation of erythropoiesis via an HIF-2α dependent increase in blood erythropoietin levels^4^ inducing a slow elevation in total Hb-mass.^7,8^ The increase in [Hb] in high altitude residents is caused by converse changes of plasma volume and total Hb-mass,^9^ too. Interestingly, at comparable total blood volume, the higher [Hb] of Andeans compared with Sherpas seems to be caused by a reduced plasma volume in the Andeans^9^ indicating that besides erythropoiesis also adjustments of plasma volume significantly affect blood oxygen transport.

Because hypoxia increases [Hb] in high-altitude residents it is a challenge to define whether an obtained [Hb] value is abnormal. The World Health Organization (WHO) provides cutoff values defining anemia^10^ and polycythemia^11^ for sea-level populations and values for adjustment accounting for [Hb] increments above the sea level.^12^ This adjustment is intended for worldwide use but appears inappropriate because of strong evidence for differences in the magnitude of [Hb] increase among world regions.^1^ As an example, Tibetan and Ethiopian altitude-populations have considerably lower mean Hb concentrations than Andean populations.^1,13^ It is therefore questionable whether fixed correction factors apply worldwide or whether region-specific adjustments are better suited for accurate diagnosis of abnormal [Hb]. Seeking to address this issue, we analyzed datasets from The Demographic and Health Surveys (DHS) Program from low- and middle-income countries where >4% of the population lives at altitudes above 1000 m.

## MATERIALS AND METHODS

### Data Source

This study is based on data from the DHS and Malaria Indicator Surveys (MIS) surveys conducted between 2000 and 2017. Only those countries were analyzed, where ≥ 4% of the studied individuals lived above 1000 m (Table 1). Because sample sizes at high altitude were too small for country-wise evaluation, countries were grouped (United Nations world region classification system, United Nations Statistics Division 2018) into Central America (CAm), South America (SAm), West and Central Africa (WCAfr), Southern Africa (SAfr), Eastern Africa (EAfr), Middle East (ME), Central and West Asia (CWA), and South and South-East Asia (SSEA). Of note, this grouping does not account for ethnicity, which has not been documented in the surveys.

**Table 1:** Countries Included in Analysis by World Region

Region	Countries	Code	Surveys	Total, n	% >1000 m	Max. alt.	b,g,f,m
Central America	Guatemala	GU	1	36,097	55	3331	b,g,f
	Honduras	HN	2	57,856	27	2159	b,g,f
South America	Bolivia	BO	2	11,209	65	4915	b,g,f
	Peru	PE	2	58,443	44	5037	b,g,f
West/Central Africa	Angola	AO	2	7496	53	2068	b,g,f
	Chad	DRC	2	29,708	20	3601	b,g,f,m
	Cameroon	CM	2	21,183	23	2407	b,g,f
	Guinea	GN	2	7984	10	2960	b,g,f
Southern Africa	Lesotho	LS	2	14,629	100	2815	b,g,f,m
	Namibia	NM	1	9625	77	2834	b,g,f,m
	Swaziland	SZ	1	10,232	22	2510	b,g,f,m
	South Africa	ZA	1	5554	60	1881	b,g,f,m
Eastern Africa	Burundi	BU	2	15,482	84	2572	b,g,f
	Ethiopia	ET	2	68,597	83	3563	b,g,f,m
	Kenya	KE	1	3074	71	2994	b,g,f
	Madagascar	MD	2	11,723	22	1706	b,g,f
	Malawi	MW	2	14,464	41	1762	b,g,f
	Mozambique	MZ	1	17,982	6	1622	b,g,f
	Rwanda	RW	2	20,111	100	2768	b,g,f
	Tanzania	TZ	2	36,444	57	2602	b,g,f
	Uganda	UG	2	16,682	93	2357	b,g,f,m
	Zimbabwe	ZW	2	37,696	68	1843	b,g,f,m
Middle East	Jordan	JO	2	19,380	19	2002	b,g,f
	Yemen	YE	1	3589	73	3091	b,g,f
Central/West-Asia	Armenia	AM	2	14,248	69	2834	b,g,f
	Azerbaijan	AZ	1	1801	4	1850	b,g,f
	Kyrgyzstan	KY	1	11,619	54	3378	b,g,f
	Tajikistan	TJ	1	14,625	26	3875	b,g,f
South/South-East Asia	India	IA	2	938,159	10	5951	b,g,f,m
	Myanmar	MM	1	16,018	9	3110	b,g,f
	Nepal	NP	2	15,991	36	3110	b,g,f
	Timor-Leste	TL	2	16,388	16	2179	b,g,f,m
Total				1,564,089			

Only those countries from DHS-surveys with >4% of samples obtained from people living at altitudes >1000 m were analyzed. The table shows the countries, number of available surveys, total number of samples, the proportion of samples collected above 1000 m, and the maximal altitude max.alt.; m) where samples had been obtained. Columns “b,g,f,m” show where samples were available from boys (b) and girls (g) age 6 to 59 months, and from females (f; not-pregnant and pregnant) and males (m) age ≥15 years.

DHS = Demographic and Health Surveys**.**

### Sampling bias

DHS and MIS surveys are nationally representative surveys that use a 2-stage cluster design sampling approach. Within each country a household listing is conducted prior to fieldwork and the data collectors have no role in selecting households. DHS and MIS survey response rates are very high, generally around 90%.^14^ In some surveys, hemoglobin data are collected within a subsample of the population and prior studies have reported no bias toward identifiable subpopulations.^15^

### Inclusion and exclusion criteria

**Figure 1.**
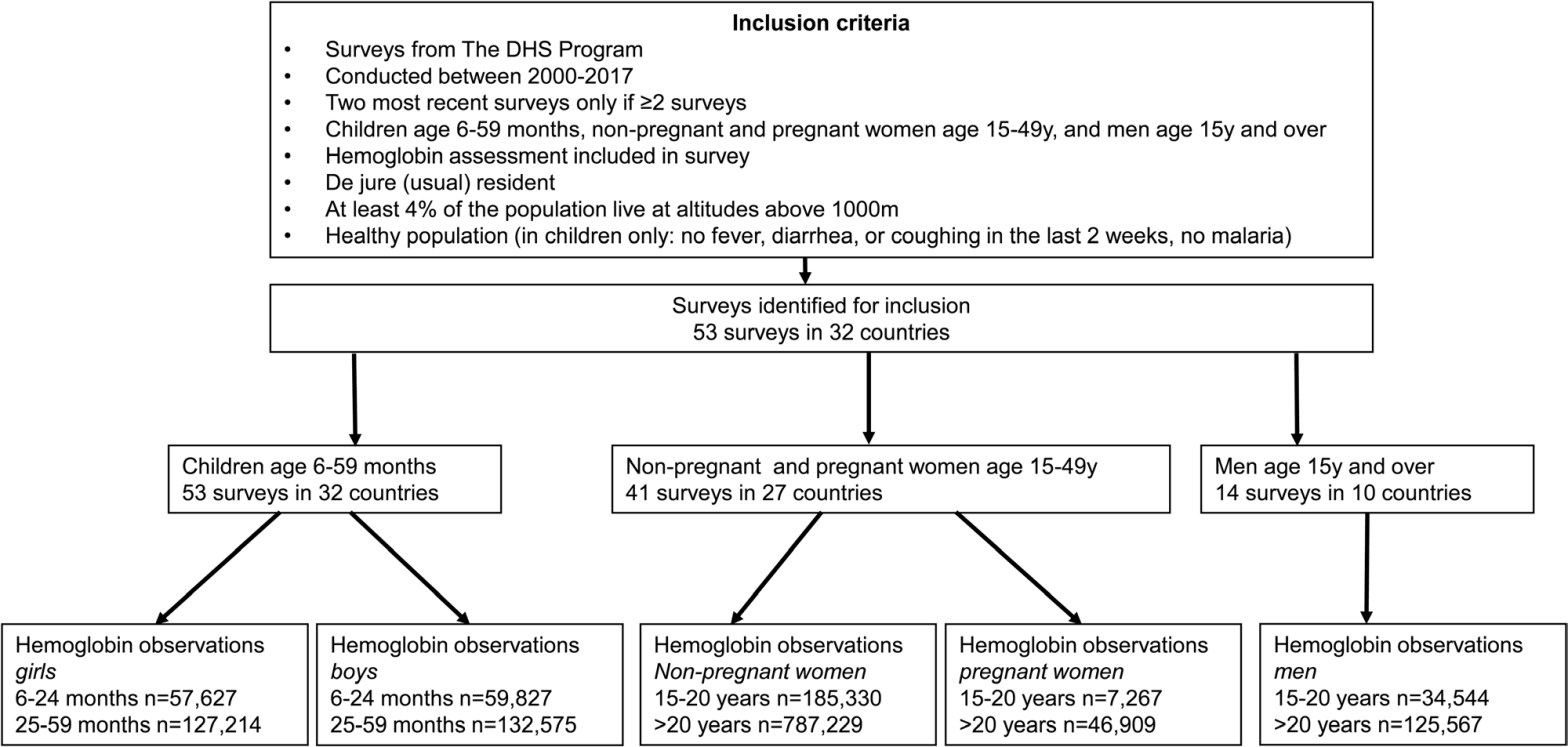
**Flow chart of selection of data from The DHS.** DHS = Demographic and Health Surveys.

Included were healthy household residents, whereby some households contributed more than one member. Excluded were children who presented with cough, diarrhea, fever, or respiratory infections within 2 weeks before blood sampling and children with malaria. The classification of “healthy” is based on illness recall and malaria rapid diagnostic test (RDT). Other markers indicative for illnesses were not obtained. Laboratory data on iron metabolism and inflammation were not obtained. Smokers with >10 cigarettes per day were excluded (<1% of the study population). Indoor cooking smoke, another source of carbon monoxide known to ultimately affect [Hb], was not applied for subgrouping. Figure 1 provides a schematic presentation of the sampling regimen.

### Groups

#### Sex

The surveys contained data on females and males. However, DHS-target populations are female and male children under age 5 and women age >15. Data on boys and girls were pooled because there is no difference in [Hb] at this age.^16–18^ Analyses shown in the article are mostly among females because of incomplete data on the males (Table 1).

#### Age

Given the age-related increase in [Hb],^16^ the data were subgrouped into children age 6–24 and 25–59 months. Adults were subgrouped into age 15–20 and >20 years. No data were available on females and males age 5–14 years.

#### Pregnancy

Separate subgroups are reported for pregnant women age 15–20 and age >20 years. We did not distinguish among pregnancy-trimesters as this information was not collected.

#### Wealth index

Micronutrient status was not recorded in DHS surveys. The DHS wealth index, a composite measure of a household’s living standard, categorizes households into 5 wealth quintiles, allowing to see how health differs between the poor and wealthy (dhsprogram.com). Therefore, the wealth index is a surrogate for the socio-economic status, which also includes access to goods, services, and assets.^19^ A value of 1 indicates the lowest and 5 the highest wealth status. Undernutrition is correlated with lower wealth status and so we used low wealth status as a surrogate for poor health.

#### Altitude (meters)

Altitude of residence was obtained from GPS readings at the sampling site. Altitude is categorized into the near sea level (−500 to 500 m), then into ranges of 500 m stepwise up to 5000 m, and into the group >5000 m. Table 1 shows the maximal altitude for each country, where the samples had been obtained.

### Hemoglobin concentration [Hb]

#### Measurements

The DHS Program follows a standard protocol for [Hb] measurement. Trained data collectors obtained a capillary blood sample (finger or heel). [Hb] was measured in a HemoCue 201+ or HemoCue 301 analyzer (Radiometer Group, Ängelholm, Sweden).

#### Removal of outliers

Because some [Hb] were very low or high for apparently healthy, nonhospitalized individuals, thresholds suggested for a healthy low-altitude population were applied,^20^ and outliers were not used for further analyses. All [Hb] values at the sea level between 7.0 and 18.0 g/dL for nonpregnant women age >15 were included. The inclusion range for pregnant women age >15 and for children of both sexes age <5 was 6.0–17.0 g/dL; it was 9.0–20.0 g/dL for men age >20.^20^ Those upper and lower limits were adjusted for altitude with 2 different methods:

1. Upper and lower sea-level limits [Hb] were altitude-corrected based on suggestions by the WHO^12^ using the formula

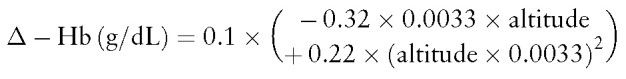



for all [Hb] regardless of world region, sex, age, and pregnancy. Suppl. Figures S1A and B show the application of this method for SSEA and for SAm, respectively. Values included for analysis are shown in black, outliers in orange, indicating that many more samples were removed in SSEA than in SAm. Numbers of outliers identified with this method are shown in Suppl. Table S2.

2. Upper and lower limits [Hb] were also altitude-corrected applying regression coefficients (Δ[Hb]/km) in Table 2. Suppl. Figures S2A and B show the application of this method for SSEA and for SAm, respectively, where values used for analysis are shown in black and outliers in orange.

**Table 2:** Intercepts and Regression Coefficients From Regression Analysis Describing the Change in [Hb] With the Residential Altitude, Sea-level to Maximal Altitude, in Children and in Nonpregnant and Pregnant Women From All World Regions

A. Change in Hb Concentration With Altitude (Slope; ΔHb/Altitude; g/dL/km)																										
World Region			CAm			SAm			WCAfr			SAfr			EAfr			ME			CWA			SSEA		
Age	Sex	Pr	Slope	SE	R^2^	Slope	SE	R^2^	Slope	SE	R^2^	Slope	SE	R^2^	Slope	SE	R^2^	Slope	SE	R^2^	Slope	SE	R^2^	Slope	SE	R^2^
6–24 mo	bg	–	0.405	0.019	0.048	0.611	0.012	0.326	0.673	0.030	0.054	0.567	0.050	0.052	0.713	0.015	0.102	−0.251	0.043	0.008	0.279	0.036	0.015	0.443	0.013	0.022
25–59 mo	bg	–	0.534	0.013	0.096	0.642	0.008	0.397	0.716	0.021	0.063	0.348	0.033	0.023	0.703	0.009	0.102	−0.285	0.029	0.009	0.381	0.025	0.032	0.493	0.009	0.030
15–20 y	f	np	0.742	0.015	0.155	0.807	0.009	0.489	0.890	0.042	0.070	0.721	0.042	0.074	1.201	0.014	0.230	0.576	0.406	*0.022*	0.548	0.038	0.046	0.560	0.009	0.032
>20 y	f	np	0.648	0.008	0.100	0.769	0.004	0.418	0.752	0.023	0.044	0.649	0.022	0.053	1.121	0.008	0.184	0.529	0.047	0.016	0.636	0.017	0.051	0.558	0.004	0.032
15–20 y	f	pr	0.676	0.062	0.119	0.824	0.047	0.432	0.719	0.151	0.028	0.478	0.180	0.035	1.173	0.067	0.145	1.004	0.782	0.043	0.087	0.268	0.001	0.585	0.059	0.025
>20 y	f	pr	0.635	0.035	0.107	0.702	0.021	0.366	0.832	0.061	0.051	0.376	0.097	0.023	1.098	0.026	0.166	0.448	0.130	0.015	0.592	0.069	0.048	0.503	0.018	0.024
**B. Intercept of regression line representing the Hb concentration at the sea level (g/dL)**																										
**World Region**			**CAm**			**SAm**			**WCAfr**			**SAfr**			**EAfr**			**ME**			**CWA**			**SSEA**		
**Age**	**Sex**	**Prg**		**Int**	**SE**		**Int**	**SE**		**Int**	**SE**		**Int**	**SE**		**Int**	**SE**		**INT**	**SE**		**Int**	**SE**		**Int**	**SE**
6–24 mo	bg	–		10.64	0.02		10.47	0.03		9.66	0.03		10.06	0.07		9.78	0.02		10.67	0.05		10.69	0.04		10.12	0.01
25–59 mo	bg	–		11.44	0.02		11.49	0.02		10.26	0.02		11.12	0.05		10.63	0.01		11.44	0.03		11.35	0.03		10.71	0.01
15–20 y	F	np		12.84	0.02		12.67	0.02		11.65	0.03		12.32	0.06		11.52	0.02		12.24	0.30		12.24	0.05		11.64	0.01
>20 y	F	np		12.87	0.01		12.74	0.01		11.72	0.02		12.36	0.03		11.57	0.01		12.04	0.04		12.05	0.02		11.66	0.01
15–20 y	F	pr		11.66	0.07		11.24	0.10		10.41	0.12		11.62	0.26		10.04	0.08		11.05	0.58		11.39	0.30		10.76	0.03
>20 y	F	Pr		11.63	0.04		11.60	0.04		10.53	0.05		11.54	0.13		10.35	0.03		11.14	0.11		11.01	0.08		10.80	0.01

A: The regression coefficients (slope; g/dl/km) ± standard error (SE), and the coefficient of determination, R^2^, which indicates the change in [Hb] with altitude of residency. B: The intercepts (int; g/dL) ± SE, which represent the calculated Hb concentration at the sea level. Both were calculated from all data obtained from sea-level up to the extreme altitudes within each region. Details on the regression statistics are provided in the Supplement. [Hb] values from boys and girls (bg) were combined because there is no sex-difference in [Hb] up to an age of ~15 years.^16–18^ Age is shown in months (mo) for the children and in years (y) for the adult, not-pregnant (np) or pregnant (pr) females (f). *P* values for multiple comparisons of intercepts and slopes are shown is the supplement in Suppl. Table S8 (difference between age/pregnancy subgroups within world regions) and in Suppl. Table S9 (difference between regions within age/pregnancy subgroups).

CAm = Central America; CWA = Central and Western Asia; EAfr = Eastern Africa; ME = Middle East; SAfr = Southern Africa; SAm = South America; SSEA = South and South-East Asia; WCAfr = West/Central Africa.

### Calculations

#### Change in [Hb] with altitude

Mean and median [Hb], standard deviation (SD), and nonparametric 95% reference intervals (95% RIs) were calculated for the above-mentioned altitude ranges for all subgroups (sex, age, and pregnancy status) within each world region. Main- and subgroup-analyses were performed with the software R, version 4.0.1.^21^ Differences in the mean [Hb] between the 500-m altitude ranges (Suppl. Table S4) within each age and pregnancy subgroup have been calculated by one way analysis of variance (ANOVA) using Tukey (HSD) for post-hoc multiple comparisons.

#### Subgroup-analysis

It was evaluated whether a relation existed between the mean [Hb] within any altitude range and the respective wealth quintiles. In addition, we compared mean [Hb] in populations from Nepal and the high-elevation states in India in close vicinity to the Himalayans (Jammu and Kashmir, Himachal Pradesh, Uttarakhand, Sikkim, and Arunachal Pradesh) with those from the remainder of the SSEA region because the residents of these Himalayan regions share genetic markers with Tibetan highland populations^22^ known for their low [Hb] at altitude when compared to South Americans.^23,24^

#### Regression analysis

Linear regression analysis was performed to obtain values of Δ[Hb]/km of altitude of females for each age-pregnancy group for each world region. The age-pregnancy grouping variable is a combination of age group and pregnancy status and consists of 6 categories: 6–24 months (6–24 mo), 25–59 months (25–59 mo), 15–20 years nonpregnant (15–20 y np), 15–20 years pregnant (15–20 y p), >20 years nonpregnant (>20 y np), and >20 years pregnant (>20 y p). To allow flexible estimation of intercepts and slopes without assuming additivity of effects across age-pregnancy groups or region groups, and for easier use, linear regression was performed in each age-pregnancy-region group separately rather than exploring the difference to a reference category and categorial covariates. Then, hemoglobin was the dependent variable and altitude (numerical) the independent variable. *P*-values for differences of intercepts and slopes between 2 age-pregnancy groups within the same region group were calculated by Wald tests and are given in Suppl. Table S8. Analogously, *P* values for differences of intercepts and slopes between 2 region groups within the same age-pregnancy group were calculated and are shown in Suppl. Table S9. All *P* values are purely descriptive and were not adjusted for multiple testing. Estimated intercepts and slopes after removal of outliers are shown in Suppl. Table S3A and B. Details on the model are provided in the supplement.

#### Calculation of prevalence of anemia and polycythemia

The prevalence of anemia and polycythemia was calculated based on the cutoff values suggested by the WHO^11,12^ for the sea level. For comparison, the 95% confidence interval (CI) lower and upper limits for a residential altitude <500 m was used. Sea-level cutoffs were adjusted for altitude using the formula suggested by the WHO^12^ or regression coefficients specific for the respective subgroups from each world region (Table 2). Because of low numbers of studied high-altitude residents in some world regions, [Hb] values were pooled to form 3 groups, boys and girls age 6–59 months, not-pregnant women age >15 years, and pregnant women age >15 years.

## RESULTS

### Sample characteristics

[Hb] was analyzed in 53 surveys from 32 countries. A single survey was available for 11 countries; there were surveys from 2 different years for 21 countries providing a total of 1,576,945 individual samples (Figure 1). Suppl. Table S1 summarizes the characteristics (age, sex, pregnancy, and wealth status) of the populations for the different world regions. Analyses were performed on 1,564,089 (Table 1) individuals since 12,856 (0.8%) of the samples were excluded because [Hb] fell outside the pre-defined ranges discussed above (Suppl. Table S2). With this approach we aimed at removing erroneous measurements, while in addition some healthy subjects may have been removed as well. The majority of the samples were available from females older than 20 years, the smallest sample size was obtained from children age 6 to 24 months. The age distribution of the individuals varied slightly between regions (Suppl. Table S1).

### Dependency of [Hb] on altitude of residence and world region

#### [Hb] near sea level

**Figure 2.**
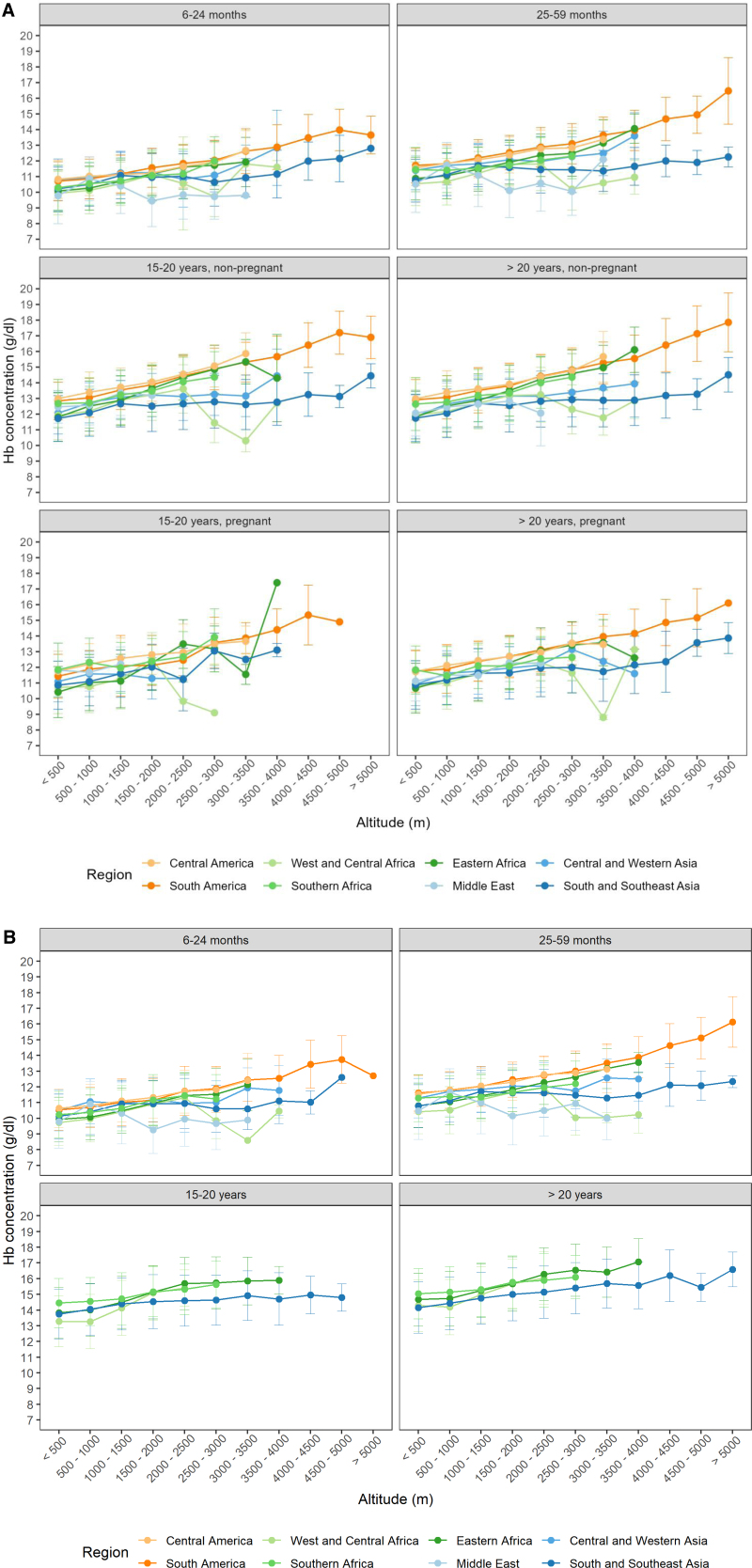
**Dependency of the [Hb] of females (A) and males (B) on age and altitude of residence in different regions of the world.** Altitudes of residence were sorted into the respective categories. Mean values of [Hb] ± standard deviation (SD) were calculated for each altitude range for different age groups of females, pregnant or not, and for males as indicated in the respective header, for the different world regions (color-codes in the inset below the figure). No SD is shown when only one data point was available. Details are also shown in look-up tables in the supplement (Suppl. Table S4).

Our data show that the mean [Hb] at altitudes <500 m (Suppl. Table S4) is all within a range of approximately 1.0 to 1.5 g/dL in the world regions within age-matched and pregnancy subgroups in females (Figure 2A) and in males (Figure 2B). ANOVA revealed that within each age group mean sea level [Hb] was significantly higher in CAm and SAm than in the other regions (not shown). The lower [Hb] in adult, not-pregnant females compared to adult males was also clearly evident (Figure 2A,B; Suppl. Table S4). Suppl. Table S4 also shows that the mean sea level [Hb] increased with age in females and males confirming earlier reports.^16^ Likewise, the [Hb] was lower in pregnant than in nonpregnant women of the respective age group.^25^ These differences are also indicated by the intercepts of the regression analysis performed in females (Table 2).

#### Increase in [Hb] with altitude

Figure 2 indicates that, in most world regions, the mean [Hb] in females (Figure 2A) and males (Figure 2B) increased with the altitude of residence. This is additionally shown in look-up tables (Suppl. Table S4), which also provide indicators of variation for different altitude ranges from <500 to >5000 m in 500 m increments, grouped by world region, sex, age, and pregnancy. In Suppl. Table S7, we show that in children and not-pregnant females from CAm and SAm, a statistically significant difference in mean [Hb] was found between most low- and high-altitude increments. In WCAfr, ME, and CWA changes in mean [Hb] were irregular and sometimes [Hb] was lower at higher than at lower altitudes; patterns differ between age groups (Figure 2; Suppl. Table S4). No statistically significant difference between mean [Hb] values of the different altitude increments was found in children and 15 to 20 years old pregnant females from WCAfr, SSEA, whereas the Hb-differences between low and very high-altitude regions were statistically significant in the not-pregnant females of both age groups.

**Figure 3.**
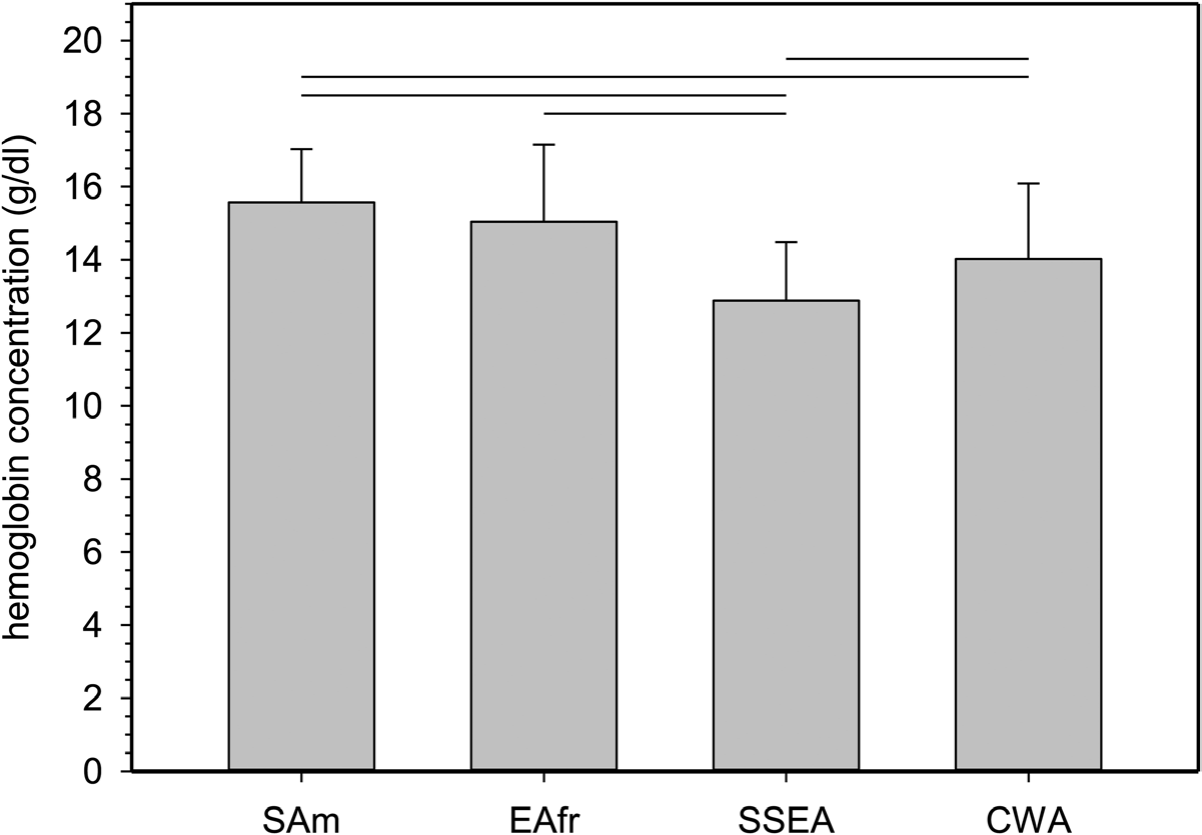
**Comparison of mean [Hb] values from not-pregnant women >15 y living between 3500 and 4000 m.** SAm, EAfr, SSEA, and CWA were chosen because they are the only regions with data available for residential altitudes >3500 m. Because sample numbers were small data from all not-pregnant women age > 15 years were combined (SAm 5413, EAfr 30, SSEA 664, CWA 70). Mean altitudes were: SAm, 3754 ± 130 m; EAfr, 3571 ± 16 m; SSEA, 3709 ± 146 m; CWA, 3708 ± 107 m). Horizontal lines indicate statistical significance between mean [Hb] (± 1 standard deviation); *P* values were <0.001. CWA = Central and Western Asia; EAfr = Eastern Africa; SAm = South America; SSEA = South and South-East Asia.

We also compared [Hb] values from the altitude range between 3500 and 4000 m, where data were only available from SAm, EAfr, SSEA, and CWA. Also, data from all not-pregnant women age >15 were combined. Numbers were too low to compare children and pregnant women. Figure 3 shows that mean [Hb] was significantly higher in SAm than in SSEA and CWA (*P* < 0.001), but was not different from EAfr (*P* = 1.0). There was also a statistically significant difference between SSEA and CWA.

#### Regression analysis, all altitudes

Table 2 shows the results of the linear regression analysis performed to quantify the increase in [Hb] with altitude. We performed two kinds of calculations: For Table 2, regressions were calculated using Hb-values obtained from all altitude ranges, that is, from the sea level to the region-specific highest altitude. Regression coefficients indicate the increase in [Hb] with altitude (ΔHb/km of altitude; g/dL/km), the intercepts indicate modeled [Hb] values for the sea level. Table 2 indicates several important aspects: (1) the ΔHb/km of children (boys and girls combined) and of females was highest in EAfr followed by CAm and SAm and was much lower in most other world regions, and even negative in children from ME. (2) Within each of the world regions, the Δ[Hb]/km was smallest in children age 6 to 24 months and increased with age. The Δ[Hb]/km was 20% to 50% lower in the children than in not-pregnant and pregnant women. It is important to note that, although correlations were statistically highly significant, R^2^ was highest in samples from SAm (>0.32) followed by EAfr (R^2^: ~0.1 to ~0.33), whereas in the other world regions R^2^ was well below 0.1 and the standard error of Δ[Hb]/km was high indicating a weak dependency of [Hb] on residential altitude. Suppl. Tables S8 and S9 show the respective P values for the difference between regressions within age groups and within regions, respectively.

#### Regression analysis, altitudes > 1000 m

Because the WHO in the current guidelines for Hb-adjustments for altitude does not suggest adjustment of [Hb] for altitudes below 1000 m,^12^ we also calculated regression coefficients only from samples obtained at altitudes >1000 m. Of note, it has been shown recently that the [Hb] increases significantly every 300 m of altitude between 200 and 2000 m above sea-level in Switzerland.^26^ Correlation analyses are summarized in Suppl. Table S3. Interestingly, in this calculation Δ[Hb]/km is considerably higher in SAm and EAfr compared with analyzing the sea level to maximal altitude (Table 2). In contrast, in SSEA the Δ[Hb]/km was much lower at altitudes >1000 m than with the lower altitudes included, and the correlation lost statistical significance in several subgroups (Suppl. Table S3). In ME, the Δ[Hb]/km became even more negative in the preschool children.

#### Subanalyses

We next evaluated whether the lack of [Hb] elevation with altitude in certain world regions was associated with the wealth status. Low wealth status is associated with undernutrition, which ultimately might impair erythropoiesis and thus affect [Hb]. Suppl. Figures S3A–H indicate that the increase—or the lack of increase—in [Hb] appeared not related to this parameter. Another subanalysis concerned the relatively small (compared with SAm) increase in [Hb] with altitude in SSEA that might be caused by specific subpopulations because of genetic similarities of Himalayan Sherpas and Tibetan highlanders.^22,24^ Unexpectedly, the pattern of [Hb]-changes with altitude was similar in residents of the Himalayan regions compared to the rest of SSEA (Suppl. Figure S4).

### Normal [Hb] for altitude and detection of abnormal values

It is obvious from the change in [Hb] with altitude in the different world regions (Figure 2; Table 2; Suppl. Table S4) that sea-level cutoff values for the detection of abnormal [Hb] values cannot be applied in high altitude residents. Furthermore, because of the different Δ[Hb]/km in different world regions and age groups, a single set of correction factors for all groups may result in false diagnoses of abnormal [Hb]. Table 3 shows values for [Hb]-adjustment for altitude for all world regions derived from regression analysis up to the maximal residential altitude where samples had been obtained in comparison with the current WHO suggestions.^12^

**Table 3:** Adjusting [Hb] for Altitude

		km	0.5	1	1.5	2	2.5	3	3.5	4	4.5	5	5.5
Region	Age/preg	Slope	g/dL	g/dL	g/dL	g/dL	g/dL	g/dL	g/dL	g/dL	g/dL	g/dL	g/dL
**WHO-worldwide**			**0**	**0.1**	**0.4**	**0.7**	**1.2**	**1.8**	**2.6**				
CAm	6–24 mo	0.336	0.2	0.3	0.5	0.7	0.8	1.0	1.2				
	25–59 mo	0.535	0.3	0.5	0.8	1.1	1.3	1.6	1.9				
	15–20 np	0.742	0.4	0.7	1.1	1.5	1.9	2.2	2.6				
	>20 np	0.648	0.3	0.6	1.0	1.3	1.6	1.9	2.3				
	15–20 pr	0.676	0.3	0.7	1.0	1.4	1.7	2.0	2.4				
	>20 pr	0.635	0.3	0.6	1.0	1.3	1.6	1.9	2.2				
**WHO-worldwide**			**0**	**0.1**	**0.4**	**0.7**	**1.2**	**1.8**	**2.6**	**3.4**	**4.4**	**5.5**	**6.7**
SAm	6–24 mo	0.621	0.3	0.6	0.9	1.2	1.6	1.9	2.2	2.5	2.8	3.1	3.4
	25–59 mo	0.642	0.3	0.6	1.0	1.3	1.6	1.9	2.2	2.6	2.9	3.2	3.5
	15–20 np	0.807	0.4	0.8	1.2	1.6	2.0	2.4	2.8	3.2	3.6	4.0	4.4
	>20 np	0.769	0.4	0.8	1.2	1.5	1.9	2.3	2.7	3.1	3.5	3.8	4.2
	15–20 pr	0.824	0.4	0.8	1.2	1.6	2.1	2.5	2.9	3.3	3.7	4.1	4.5
	>20 pr	0.702	0.4	0.7	1.1	1.4	1.8	2.1	2.5	2.8	3.2	3.5	3.9
**WHO-worldwide**			**0**	**0.1**	**0.4**	**0.7**	**1.2**	**1.8**	**2.6**	**3.4**			
WCAfr	6–24 mo	0.648	0.3	0.6	1.0	1.3	1.6	1.9	2.3	2.6			
	25–59 mo	0.712	0.4	0.7	1.1	1.4	1.8	2.1	2.5	2.8			
	15–20 np	0.890	0.4	0.9	1.3	1.8	2.2	2.7	3.1	3.6			
	>20 np	0.752	0.4	0.8	1.1	1.5	1.9	2.3	2.6	3.0			
	15–20 pr	0.719	0.4	0.7	1.1	1.4	1.8	2.2	2.5	2.9			
	>20 pr	0.832	0.4	0.8	1.2	1.7	2.1	2.5	2.9	3.3			
**WHO-worldwide**			**0**	**0.1**	**0.4**	**0.7**	**1.2**	**1.8**					
SAfr	6–24 mo	0.511	0.3	0.5	0.8	1.0	1.3	1.5					
	25–59 mo	0.331	0.2	0.3	0.5	0.7	0.8	1.0					
	15–20 np	0.721	0.4	0.7	1.1	1.4	1.8	2.2					
	>20 np	0.649	0.3	0.6	1.0	1.3	1.6	1.9					
	15–20 pr	0.478	0.2	0.5	0.7	1.0	1.2	1.4					
	>20 pr	0.376	0.2	0.4	0.6	0.8	0.9	1.1					
**WHO-worldwide**			**0**	**0.1**	**0.4**	**0.7**	**1.2**	**1.8**	**2.6**	**3.4**			
EAfr	6–24 mo	0.717	0.4	0.7	1.1	1.4	1.8	2.2	2.5	2.9			
	25–59 mo	0.703	0.4	0.7	1.1	1.4	1.8	2.1	2.5	2.8			
	15–20 np	1.201	0.6	1.2	1.8	2.4	3.0	3.6	4.2	4.8			
	>20 np	1.121	0.6	1.1	1.7	2.2	2.8	3.4	3.9	4.5			
	15–20 pr	1.173	0.6	1.2	1.8	2.3	2.9	3.5	4.1	4.7			
	>20 pr	1.098	0.5	1.1	1.6	2.2	2.7	3.3	3.8	4.4			
**WHO-worldwide**			**0**	**0.1**	**0.4**	**0.7**	**1.2**	**1.8**	**2.6**				
ME	6–24 mo	−0.241	−0.1	−0.2	−0.4	−0.5	−0.6	−0.7	−0.8				
	25–59 mo	−0.323	−0.2	−0.3	−0.5	−0.6	−0.8	−1.0	−1.1				
	15–20 np	0.576	0.3	0.6	0.9	1.2	1.4	1.7	2.0				
	>20 np	0.529	0.3	0.5	0.8	1.1	1.3	1.6	1.9				
	15–20 pr	1.004	0.5	1.0	1.5	2.0	2.5	3.0	3.5				
	>20 pr	0.448	0.2	0.4	0.7	0.9	1.1	1.3	1.6				
**WHO-worldwide**			**0**	**0.1**	**0.4**	**0.7**	**1.2**	**1.8**	**2.6**	**3.4**			
CWA	6–24 mo	0.258	0.1	0.3	0.4	0.5	0.6	0.8	0.9	1.0			
	25–59 mo	0.373	0.2	0.4	0.6	0.7	0.9	1.1	1.3	1.5			
	15–20 np	0.548	0.3	0.5	0.8	1.1	1.4	1.6	1.9	2.2			
	>20 np	0.636	0.3	0.6	1.0	1.3	1.6	1.9	2.2	2.5			
	15–20 pr	0.087	0.0	0.1	0.1	0.2	0.2	0.3	0.3	0.3			
	>20 pr	0.592	0.3	0.6	0.9	1.2	1.5	1.8	2.1	2.4			
**WHO-worldwide**			**0**	**0.1**	**0.4**	**0.7**	**1.2**	**1.8**	**2.6**	**3.4**	**4.4**	**5.5**	**6.7**
SSEA	6–24 mo	0.448	0.2	0.4	0.7	0.9	1.1	1.3	1.6	1.8	2.0	2.2	2.5
	25–59 mo	0.500	0.3	0.5	0.8	1.0	1.3	1.5	1.8	2.0	2.3	2.5	2.8
	15–20 np	0.560	0.3	0.6	0.8	1.1	1.4	1.7	2.0	2.2	2.5	2.8	3.1
	>20 np	0.558	0.3	0.6	0.8	1.1	1.4	1.7	2.0	2.2	2.5	2.8	3.1
	15–20 pr	0.585	0.3	0.6	0.9	1.2	1.5	1.8	2.0	2.3	2.6	2.9	3.2
	>20 pr	0.503	0.3	0.5	0.8	1.0	1.3	1.5	1.8	2.0	2.3	2.5	2.8
**Mean of all**			**0.3**	**0.6**	**0.9**	**1.2**	**1.5**	**1.8**	**2.2**	**2.7**	**2.8**	**3.1**	**3.5**
**SD**			**0.2**	**0.3**	**0.4**	**0.6**	**0.7**	**0.9**	**1.1**	**1.0**	**0.6**	**0.6**	**0.7**

The [Hb] (g/dL) should be subtracted from a [Hb] measured at altitude for adjustment to the sea level, or should be added to a reference-value at the sea level to calculate the corresponding value at a given altitude. The table compares the correction values provided by the WHO^12^ (gray shaded lines) with the ones derived from linear regressions (slope; increase in [Hb] with altitude (g/dL/km) from Table 2). The altitude is shown in km. Values are only shown for the altitude regions available within each region. Mean of all are the mean values ± standard deviation (SD) of all adjustment-values calculated from our regressions for all age and pregnancy subgroups and all world regions.

CAm = Central America; CWA = Central and Western Asia; EAfr = Eastern Africa; ME = Middle East; np = not-pregnant; pr = pregnant; SAfr = Southern Africa; SAm = South America; SSEA = South and South-East Asia; WCAfr = West/Central Africa.

Table 3 shows that calculated adjustment values differ in part considerably from the WHO-suggestion, particularly in the children, and that the deviation from the WHO-suggestion varies considerably between world regions and the residential altitude. In altitude ranges between 500 and approximately 2000 m our calculated values for [Hb] adjustment are higher than those suggested by the WHO in most world regions and subgroups, due to our linear approach compared with the quadratic function used by the WHO. There was good agreement between both approaches at 2500 and 3500 m, but not uniformly in all world regions. At altitudes >3000 to 3500 m, WHO adjustments are up to ~3 g/dL higher than those derived by our linear approach. Importantly, in SSEA the WHO-adjustment is vastly higher than that derived from our linear regression at altitudes >2500 m deviating by up to 4 g/dL.

**Figure 4.**
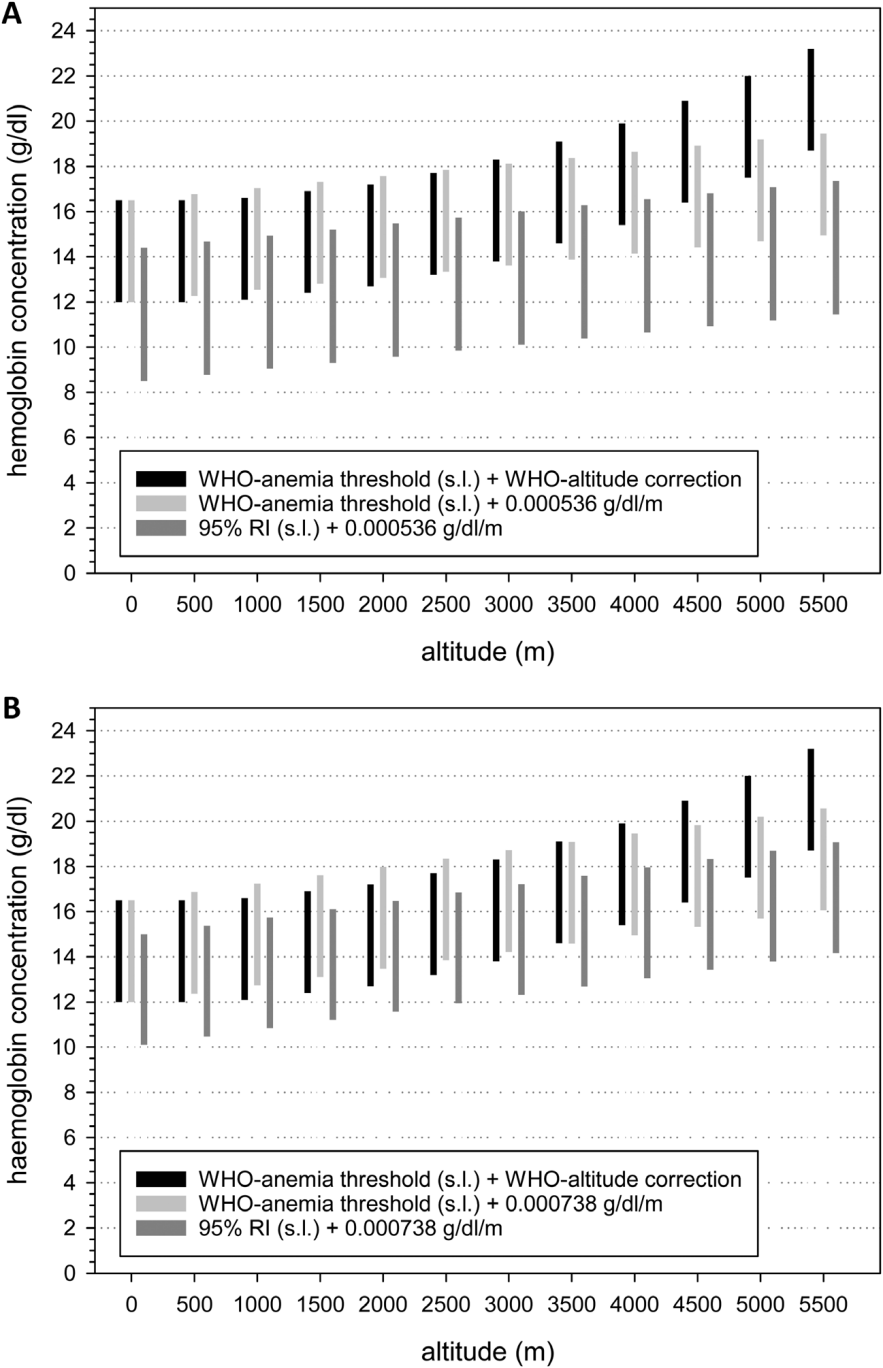
**Comparison of normal ranges for [Hb] for females from South/South-East Asia (A) and from South America (B) obtained with different models.** Each bar represents a reference interval obtained with a different approach: black bars: cut off values for anemia and polycythemia suggested by the WHO for sea-level and altitude adjustment according to the WHO. Light gray bars: cutoff values for anemia and polycythemia suggested by the WHO for the sea level and altitude adjustment with the delta-Hb/km from our regression analysis. Dark gray bars: lower and upper limit of the reference interval calculated for the sea level (shown in Suppl. Table S4) and its altitude adjustment with our linear regressions. WHO = World Health Organization.

Another approach to define whether a measured [Hb] value falls within the normal range might be using look-up tables. Suppl. Table S4 lists mean [Hb] values, one SD, and the upper and lower limits of the 95% RIs as indicators of variation grouped by sex, age, and pregnancy in 500 m altitude-increments within each of the world regions. Because these values were calculated after removing outliers, [Hb] falling within these ranges can be considered normal. Figure 4 and Suppl. Figure S5 show a graphical comparison of normal [Hb] ranges as bars: ranges indicated by black and light gray bars were based on the sea-level thresholds for anemia and polycythemia suggested by the WHO,^12^ dark gray bars were based on the upper and the lower limits of 95% RI for samples collected at altitudes below 500 m (Suppl. Table S4). Black bars show the adjustment for altitude using the WHO-model,^12^ for the light and dark gray bars linear regressions (Table 2) was applied. These figures show the discrepant [Hb] normal-ranges and indicate that the ranges based on the WHO approach are higher than those obtained with the other models, particularly in SSEA. The figure also shows that the ranges derived from the 95% RIs are shifted toward lower [Hb] values at any specific altitude range, which indicates that anemia thresholds might be lower than those suggested by the WHO.

### Prevalence of anemia and polycythemia

**Figure 5.**
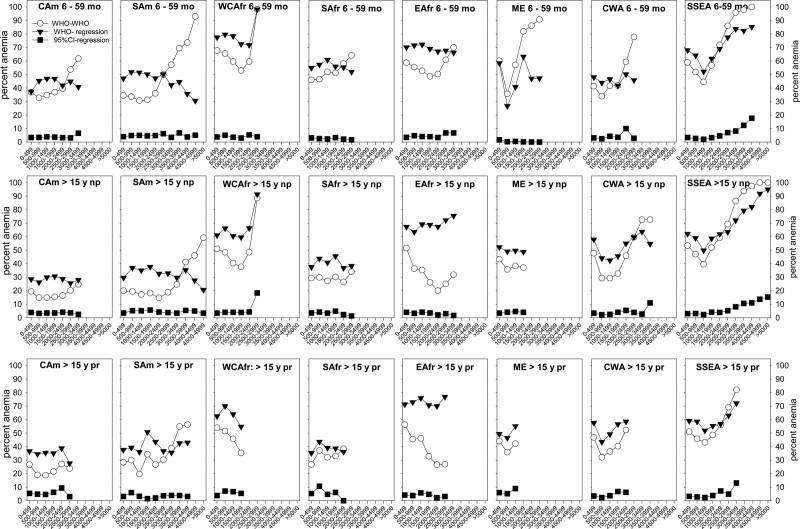
**Prevalence of anemia in boys and girls age 6 to 59 month, and in not-pregnant and pregnant women older than 15 years in different world regions; comparison of methods of evaluation.** It shows the percent-anemics. The number of anemic individuals and total number of individuals in the respective groups are shown in Suppl. Table S5. “WHO-WHO” (open circles) indicates that anemia thresholds suggested by the WHO for the sea level (boys, girls, and pregnant women: 11 g/dL; nonpregnant women >15 years: 12 g/dL) and the WHO-suggestion for altitude-adjustment^25^ were used. “WHO-regression” (black inverse triangles) indicates the use of the WHO-anemia thresholds for the sea level,^25^ which were adjusted for altitude using linear equations. “95% CI-regression” (black squares) indicates that the lower level of the 95% confidence interval for Hb-values obtained below a residential altitude of 500 m above the sea level were used as anemia thresholds, which were adjusted for altitude using the linear regression. Age groups according to the WHO^25^ (preschool children age 6 to 59 months (mo), and nonpregnant (np) and pregnant (pr) women age > 15 years (y), rather than more detailed grouping shown in Figure 2 and in the look-up tables because of low numbers in some age-subgroups, particularly at high altitudes. Values are only shown when the total number of individuals in the respective group was >50.

The prevalence of anemia and polycythemia was calculated using the sea-level-thresholds and their adjustment for altitude as suggested by the WHO^12^ that is set for use in all world regions, and by adjustment for altitude using our world region- and age- and pregnancy-specific Δ[Hb]/km (Table 2). For comparison, the lower and upper limits of the 95% RI calculated from data after removal of abnormally low and high [Hb] values were applied to the complete dataset. Because in some groups the number of samples was small, particularly in the high-altitude ranges, children of both age groups as well as females >15 years were pooled resulting in the groups of children age 6 to 59 months, not-pregnant women, and pregnant women age > 15 years. Figure 5 compares the prevalence of anemia, and Suppl. Table S5 provides absolute numbers and group-sizes.

#### Prevalence of anemia near sea level <500 m

Based on the WHO-thresholds, in all world regions, anemia-prevalence was higher in the children than in pregnant and nonpregnant females. In all age- and pregnancy groups, the highest prevalence of anemia was found in WCAfr, EAfr, ME, and SSEA.

#### Prevalence of anemia at altitudes >500 m

Based on the WHO model, there was a pronounced increase in anemia-prevalence with increasing altitude in most world regions (Figure 5, Suppl. Table S5). Prevalence was >80% in children from SAm, WCAfr, ME, and SSEA, and in not-pregnant females from WCAfr and SSEA in the extreme-altitude ranges. Anemia prevalence in pregnant females was lower than in the not-pregnant females in most regions. Using WHO-sea-level-thresholds and altitude adjustment with our linear regressions shows a somewhat increased anemia prevalence at the more moderate altitudes but also a lower prevalence at high-altitude ranges in nearly all world regions. The altitude, where the two curves intersect, is in the range of approximately 2500 m. Using the sea-level 95% CI-lower limit as anemia-threshold and linear altitude adjustment indicates a much lower prevalence, but also a slight increase with increasing residential altitude.

#### Prevalence of polycythemia at low and high altitudes

The prevalence of polycythemia (Suppl. Table S6) is, in general, much lower than that for anemia. Using literature-values for polycythemia-thresholds from the sea level^16,27^ and WHO- and regression-derived altitude adjustments results in the prevalence of polycythemia below 1%, even at altitudes >2000 m in most world regions. Only in children and adult, nonpregnant women the prevalence reaches a prevalence up to 5% at altitudes >4000 m in SAm (Suppl. Table S6). In contrast, using the 95% CI and regressions-derived altitude adjustment, the prevalence for polycythemia is somewhat higher (1% to 2.5% at altitudes up to ~2500 m), and increases with increasing altitude reaching values up to ~11% in not-pregnant females living at altitudes >4000 m (Suppl. Table S6).

## DISCUSSION

Our analysis of DHS data shows statistically significant correlation between residential altitude and [Hb] values (Δ[Hb]/km), but also pronounced differences in its magnitude between world regions, confirming earlier results^1^ and literature comparing specific altitude regions of individual countries.^13,28^ The highest values for Δ[Hb]/km were found in adult females from the 3 African and the 2 American regions (1.2 to 0.7 g/dL/km), whereas the Δ[Hb]/km was below 0.6 g/dL/km in the ME and the 2 Asian regions (Table 2). These values compare well with previous data from a meta-analysis.^1^ We report here for the first time that in all world regions the Δ[Hb]/km was approximately 20%–25% smaller in the children than in the adult females. Importantly, in the children from ME, [Hb] even decreased with increasing altitude. It is of note that, although all correlations were statistically significant, in some age-subgroups from ME, WCAfr, and CWA, mean [Hb] values sometimes increased with altitude but then decreased at higher altitudes or there was no consistent change (Figure 2, Suppl. Table S4). [Hb] of girls and boys up to age of 5 years were similar at comparable altitude of residency, which is consistent with reports on low-altitude [Hb]^16^ indicating that in preschool children no sex-specific adjustment is required.

These findings have important clinical implications because applying a single set of values for adjusting [Hb] for altitude for worldwide and age-independent use as currently suggested by the WHO^12^ may result in erroneous diagnosis of abnormal [Hb], particularly in children from all world-regions and for adult females in the Asian regions. The situation is complicated by the observation that, when only [Hb] values from altitudes >1000 m were used for calculation, the Δ[Hb]/km decreased considerably in the Asian regions and in WCAfr, and the correlation lost statistical significance in some subgroups. In contrast, the Δ[Hb]/km became even somewhat higher in CAm, SAm, SAfr, and EAfr (Suppl. Table S3) compared to the analysis including low-altitude data shown in Tables 2. Because of the divergent values of Δ[Hb]/km the diagnosis of anemia and polycythemia in high-altitude residents remains challenging.

Most studies have not examined the Δ[Hb]/km across countries. Rather, studies have focused on [Hb] values at high altitudes from different countries for comparison.^13^ Such comparisons are limited by the fact that altitude regions are often not the same. Altitudes studied in Ethiopia were considerably lower than those in South America or in the Tibet. Given the magnitudes of Δ[Hb]/km in the various regions (Table 2), this might result in erroneous conclusions. The DHS dataset contains sufficient data at altitudes >3500 m only from SAm, EAfr, CWA, and SSEA from adult, not-pregnant women age >15 years but not from the other subgroups. Comparison (Figure 3) shows that [Hb] from EAfr was not different from SAm, but that [Hb] from SSEA and CWA was lower than in the other regions.

Genomic diversity might explain the differences in the Δ[Hb]/altitude and in mean [Hb] values at certain altitudes between world regions. Such genomic variations have been found in Tibetans, where polymorphisms in EPAS1 and EGLIN1 genes had been associated with low [Hb] pointing to a role of erythropoietin (EPO) and erythropoiesis.^24,29,30^

A different aspect had been put forward by Stembridge and colleagues,^9^ who showed that a smaller plasma volume at comparable total blood volume in South American Quechuas accounts for the difference in [Hb] to Nepalese Sherpas. On a first look, this result seems to speak against a major role of erythropoiesis in adjusting [Hb] in high altitude residents, unless the prolyl-hydroxylase/HIF-system and/or erythropoietin also control plasma volume. There is in fact some evidence that such interaction might exist: Treatment with EPO has been shown to decrease plasma volume,^31^ probably by affecting proximal tubular reabsorption.^32^ On the other hand, head-down tilt increased central blood volume and also elevated plasma EPO-levels.^33^ Therefore, Tibetans and genetic relatives having a hyporesponsive HIF-system^29,34^ due to a gain of function mutation of PHD2,^35^ have a lower or even missing EPO response to hypoxia, as seen by similarly low EPO-levels in high-altitude Sherpas and non-Sherpa,^36^ which would result in an increased plasma volume compared with Andeans carrying the fully responsive HIF-system and elevated EPO.^37^ If this link between EPO-levels and plasma volume, which are the main determinants of [Hb], is based on these known genetic variations, and if those variations existed throughout the regions we have analyzed, one might speculate that populations carrying a hyperresponsive HIF-system have migrated from Africa through Asia and on different routes to the Americas,^38^ where the high responsive HIF-system was preserved. A Denisovan-related introgression^39^ resulted in a hyporesponsive HIF-system restricted to Asian regions. We cannot define which of these two [Hb] and HIF-systems provides a selective advantage, and secondary adaptations might also have improved survival in the specific world regions. Such likely HIF-independent effects might explain the lower ΔHb/km in children, and the lower [Hb] in pregnant women caused mainly by a disproportionate increase in plasma volume despite increased total erythrocyte mass.^40^ Targeted genetic and functional analysis of the oxygen transport system are required to test the assumption on migration and adaptive advantages.

[Hb] is also affected by nutritional iron- and vitamin-supply essential for erythropoiesis.^4^ This is of particular importance and thus, the DHS focusses on low and middle-income countries, where malnutrition is likely to occur. It is well established that iron supplementation increases [Hb] in iron-deficient children and child-bearing women living at high altitude^37,41,42^ and prevents a [Hb]-decrease in non-anemic, SAm school children.^43^ Our dataset did not contain such information. Therefore, we analyzed the wealth status as a surrogate for general health and well-being, which might include those parameters. Unexpectedly, we did not observe a systematic effect of the wealth index on the magnitude of change in mean [Hb] values with altitude (Suppl. Figure S3). Comparison of our results with other studies on [Hb] in healthy high-altitude residents revealed comparable results on Δ[Hb]/altitude.^17,18^ In summary, these results indicate that the nutritional status seems not to be a major determinant of [Hb] in healthy high-altitude residents. Severe nutritional deficits, however, will certainly affect erythropoiesis and cause anemia.

The health status is another confounder affecting [Hb]. Chronic inflammation causes hypoferremia and anemia by upregulation of hepcidin.^44,45^ The health status of the studied children is quite well characterized, but records on adults are mostly based on interviews, and chronic inflammation was not recorded. Individuals with reported health problems, amongst others Malaria, were excluded from analysis, but diseases such as thalassemia, sickle cell disease, hookworm infections, and chronic mountain sickness were not surveyed.

Along with the wealth status, cooking and heating systems might differ. As such, people might be affected by exposure to elevated levels of carbon monoxide (CO) by open fire in closed environments. Also, tobacco smoking increases CO-exposure. CO binds to hemoglobin with high affinity and prevents binding of oxygen.^6^ CO-exposure is thus another confounder affecting [Hb] in an altitude-independent manner,^46^ and CO and altitude effects on [Hb] might be additive because elevated CO stimulates erythropoiesis^47^ and decreases plasma volume.^31^ Both kinds of exposure had been recorded in the DHS-surveys, but were not accounted for in our calculations due to low sample numbers, particularly in the very high-altitude regions.

### Defining “normal” [Hb] values for high-altitude residents

The profound difference in Δ[Hb]/altitude between world regions raises the question for appropriately defining normal [Hb] values for high altitude residents. Often, normal ranges are defined using the 95% RI or CI of [Hb] of a healthy population. We determined the 95% RI for all world regions and for the respective subgroups (age, sex, pregnancy) for 500 m altitude-increments (Suppl. Table S4), and also performed regression analysis (Table 2 and Suppl. Table 3) to acquire a Δ[Hb]/km of altitude to estimate adjustment factors specific for each world region. At very high altitudes these estimates differ considerably from the values suggested by the WHO for altitude adjustment of [Hb]^12^ (Figure 4 and Suppl. Figure 5), where the WHO-values are always higher than what we have found (Table 3). On the other hand, our correction factors derived from linear equations are higher than those from the WHO at altitudes between 500 m and approximately 1500 m. It is important to note that the WHO approach was based on data from SAm and thus does not account for differences between world regions.^12^ Because of these large deviations it is difficult to decide, which of these approaches provides the most accurate [Hb] normal ranges.

An important consequence of the model-dependent discrepant normal ranges for high altitude [Hb] is a false estimate of the prevalence of anemia and polycythemia, which might result in unnecessary treatment of large populations. The WHO model predicts a much higher prevalence of anemia than all others (Figure 5, Suppl. Table S5). Discrepancies between WHO- and data-analysis-based diagnosis of anemia in children have also been reported from Peru^17,18,48^ and Bolivia.^49^ The Bolivian study shows that iron deficiency contributed only marginally^49^ to [Hb] considered below-normal based on the WHO-guidelines indicating that those [Hb] may have been diagnosed false-low. By contrast, the WHO-model predicts a very low prevalence of polycythemia even in SAm; it is practically zero in SSEA (Suppl. Table S6). Polycythemia-prevalence is even lower than the prevalence of chronic mountain sickness, which was approximately 15% in residents of Cerro de Pasco (4300 m, Peru),^50^ which puts these low estimates into question. It is unclear, whether these discrepancies are due to the altitude adjustment factors, the thresholds for anemia and polycythemia for sea-level, or both.

### Limitations of the study

As mentioned above, we could not rule out effects of inflammation, iron and micronutrient deficiencies because the DHS datasets do not include respective biomarkers. Also, the health status was addressed as best as we could.

The data from different countries are grouped by world regions. However, the number of countries per world region is sometimes small (Table 1), and many countries with large populations living at altitudes > 1000 m (eg, China, Tibet) were not covered by the DHS-surveys. Data on females and males age 5 to 14 years were not available. Another limitation is the small number of individuals from extreme-altitude regions.

## SUMMARY AND CONCLUSION

Results from this study—complemented by other reports^1,13^—clearly demonstrate world region-specific differences in Δ[Hb]/altitude with highest value found in Africans and South Americans, and the smallest in Asians. Here, we show for the first time that in all world regions the Δ[Hb]/altitude is less in children than in adult women. Because of this diversity we generated look-up tables defining a normal range of Hb for each studied world-region and the respective subgroups.

This divergence in Δ[Hb]/altitude indicates profound problems in the diagnosis of anemia and polycythemia in high-altitude residents because until now their diagnosis was based on a single algorithm for altitude adjustment of [Hb] as presented by the WHO for world region-unspecific, and age-independent use.^12^ These discrepancies need to be overcome by re-defining altitude-dependent normal [Hb] ranges. The look-up tables and the regression coefficients might be useful tools. In that, the results of the present study contribute to current efforts of the WHO in generating improved tools to define thresholds for the diagnosis of anemia but also for polycythemia in high altitude residents. Furthermore, a more accurate definition of anemia might improve deciding on the necessity of costly iron and vitamin supplementation in different regions of the world.

## ACKNOWLEDGMENTS AND SOURCES OF FUNDING

RB’s participation in this research was supported by the United States Agency for International Development (USAID) through The DHS Program (#AIDOAA-C-13-00095). MG is supported by the Swiss National Science Foundation as well as by the Fondation Botnar. We want to thank Prof. Sant-Rayn Pasricha, WEHI, Parkville, Australia, and Dr. Sorrel Namaste, ICF, Rockville, MD, USA, for most valuable discussion of the manuscript. We thank the Bill and Melinda Gates Foundation (INV-008034) for supporting publication.

## AUTHOR CONTRIBUTIONS

MUM, MG, RB, and HM made substantial contributions to the conception or design of the work. MUM, MG, RB, SK, SS, and HM gave final approval for the manuscript to be published and agreed to be accountable for all aspects of the work in ensuring that questions related to the accuracy or integrity of any part of the work are appropriately investigated and resolved. SK, SS, and HM analyzed, MUM, MG, RB, SK, SS, and HM interpreted data for the article. MUM, MG, RB, SK, SS, and HM critically revised the manuscript for important intellectual content. HM drafted the manuscript and prepared the final draft. HM (corresponding author) had full access to all data and had the final responsibility for submission.

## DATA AVAILABILITY

Data are available in the manuscript and supplementary digital content. No additional datasets were created as a result of this study.

## DISCLOSURES

MUM is a HemaSphere editor. The authors have no conflicts of interest to disclose.

## Supplementary Material


