# Supplementary material for: The Increase in Hemoglobin Concentration With Altitude Differs Between World Regions and Is Less in Children Than in Adults
Source: Hemasphere. 2023 Apr 5;7(4):e854. doi: 10.1097/HS9.0000000000000854 (PMC10082317; doi:10.1097/HS9.0000000000000854)
Supplement: Supplementary file 1 [file hs9-7-e854-s001.docx]

**Supplementary Information for**

The Increase in Hemoglobin Concentration with Altitude Differs between World Regions and is Less in Children than Adults

HemaSphere-2022-0074

**Details to the regression analysis**

Linear regression analysis was performed to model the relation of Hb and altitude in each age-pregnancy group for each world region. The age-pregnancy grouping variable is a combination of age group and pregnancy status and consists of 6 categories: 6-24 months (6-24 mo), 25-59 months (25-59 mo), 15-20 years non-pregnant (15-20 y np), 15-20 years pregnant (15-20 y pr), > 20 years non-pregnant (> 20 y np), > 20 years pregnant (> 20 y pr). To allow flexible estimation of intercepts and slopes without assuming additivity of effects across age-pregnancy groups or region groups, linear regression was performed in each age-pregnancy-region group separately. There, hemoglobin was the dependent variable and altitude (numerical) the independent variable. This is equivalent to estimating the coefficients in a full model where altitude, age-pregnancy group, region group and all interaction terms between those three variables are included. Descriptive p-values for differences of intercepts and slopes between two age-pregnancy groups within the same region group were calculated by Wald tests. This was done by estimating a linear regression model including altitude, age-pregnancy group and the interaction between both as independent variables. Note that the categorical variable age-pregnancy group is dummy-coded for inclusion in the regression model, resulting in 5 binary variables (ap1, …, ap5). Thus, the model equation is

$$\mathrm{hemoglobin}=\beta_{0}+\beta_{1}\cdot\mathrm{altitude}+\beta_{0, \mathrm{ap}, 1}\cdot\mathrm{ap}1+\beta_{0, \mathrm{ap}, 2}\cdot\mathrm{ap}2+\ldots+\beta_{1, \mathrm{ap}, 1}\cdot\mathrm{ap}1\cdot\mathrm{altitude}+\ldots,$$

where e.g. ap2 is the binary dummy-variable indicating whether an individual is in age-pregnancy group 2 (reference group is coded with 0). This means that the model includes 5 interaction terms (one for every dummy-variable). For every coefficient, the p-value of the Wald test was calculated, relating to the comparison of the respective dummy-variable-category to the reference category. For the coefficients of the interaction terms, these tests can be viewed as tests for interaction. By applying this procedure with different reference groups, p-values for every pairwise comparison of categories were calculated. These p-values are given in Table S8 and are used as indicators whether non-random differences of intercepts or slopes between age-pregnancy groups occur. Analogously, descriptive p-values for differences of intercepts and slopes between two region groups within the same age-pregnancy group were calculated and given in Table S9.

**Supplemental figures**

**Figure S1A,B: Individual Hb concentrations from females from South/South-East Asia (A and South America (B)** in dependence of altitude, and outliers removed for further calculations. At sea-level, outliers were those [Hb] outside the range of 7.0 g/dL and 18.0 g/dL for non-pregnant women age >15, for pregnant women age >15 and the children of both sexes age <5 between 6.0 g/dL and 17.0 g/dL, and between 9.0 g/dL to 20.0 g/dL for men age > 20 ^1^. These cut-off values were corrected for altitude according to the WHO ^2^. Regression analysis was performed on both datasets, all data and those remaining after outlier-removal. Intercepts and regression coefficients are shown in the supplementary table S2). Symbols with orange edges: data that were omitted as described in the methods section; symbols with black edges: data within the pre-defined range (see methods section). Regression lines were calculated for all data (black and orange symbols) and after removal of outliers (black symbols only). Lines appear broken with intermittent coloring due to almost identical intercepts and regression coefficients; orange line: regression using all data; blue line: regression after excluding outliers).

**Figure S1A: South/South-East Asia (SSEA)**

*
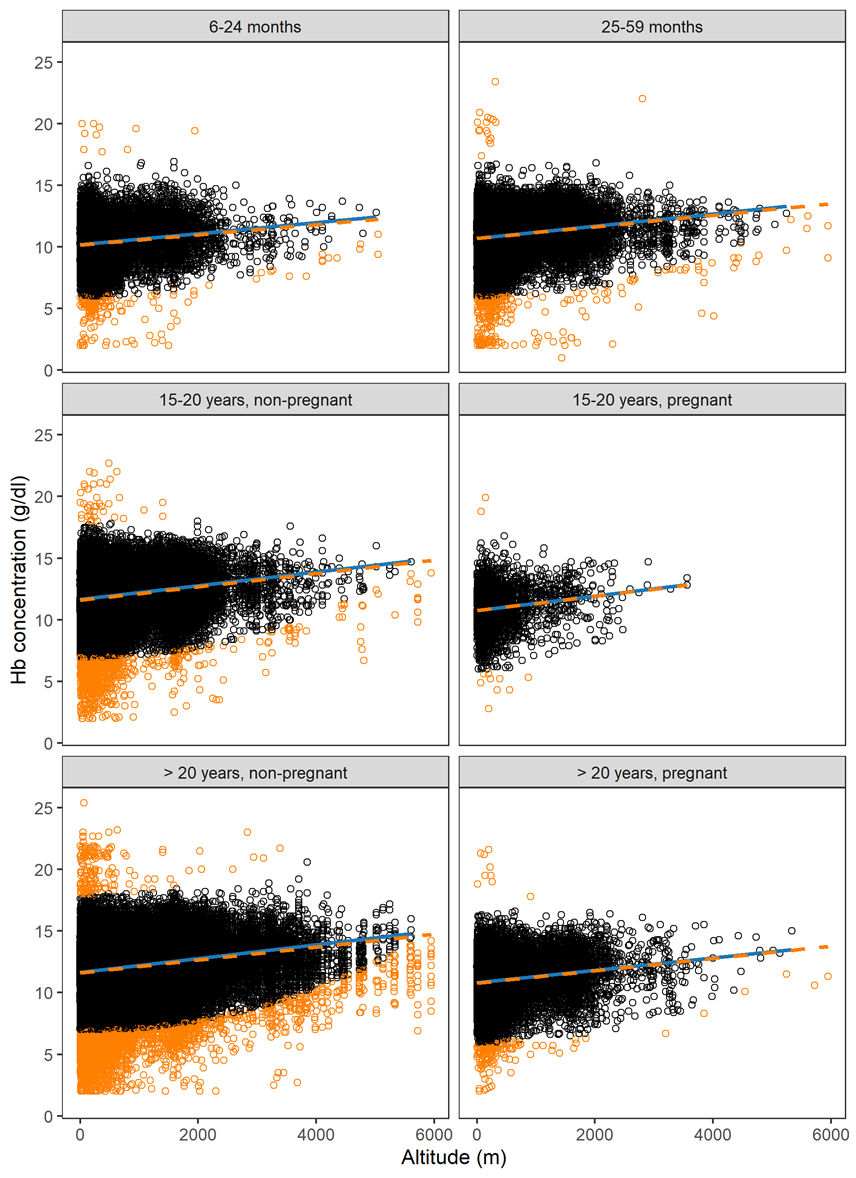
*

**Figure S1B: South America (SAm)**


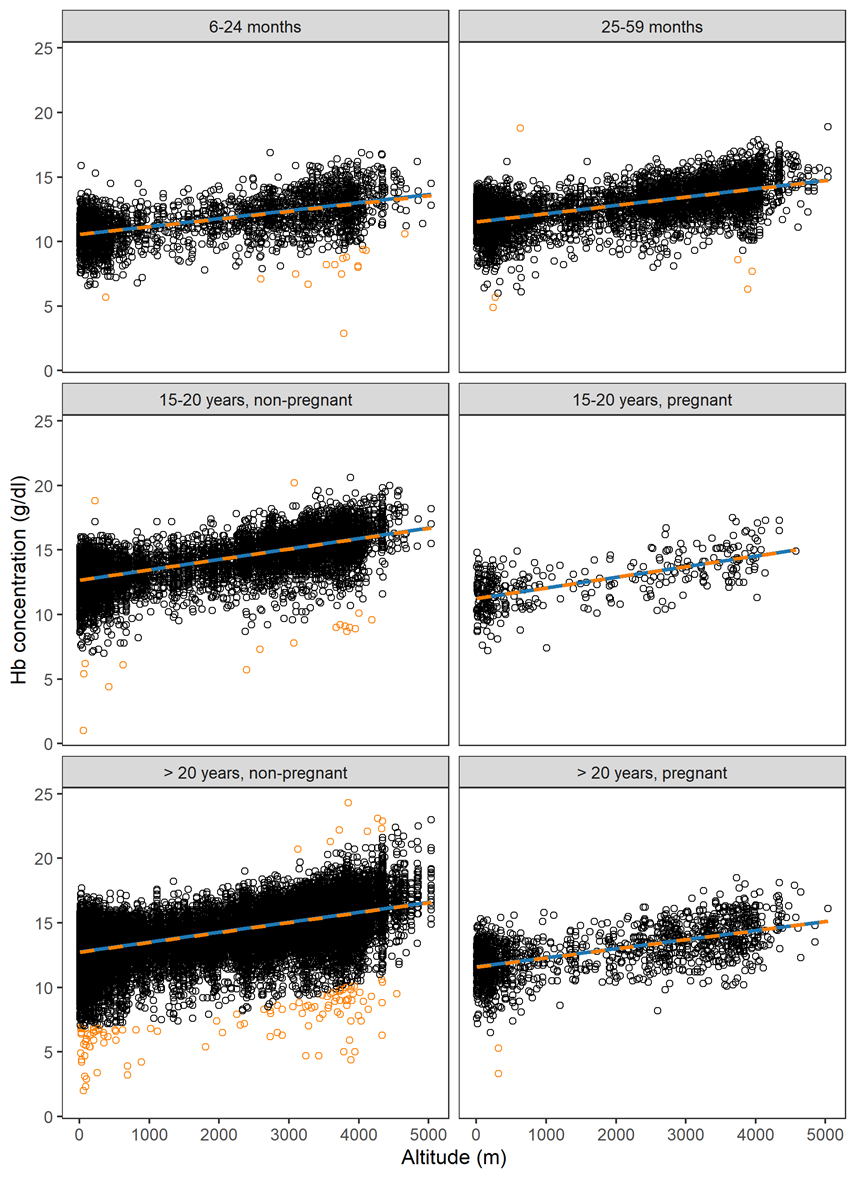


**Figure S2A,B: Individual Hb concentrations from females from South/South-East Asia (A and South America (B)**. Same as in figure S1AB, but sea-level cut-off values for outlier removal were corrected using the linear regression obtained from all data. Regression equations are shown in table S2.

**Figure S2A. South/South-East Asia (SSEA)**


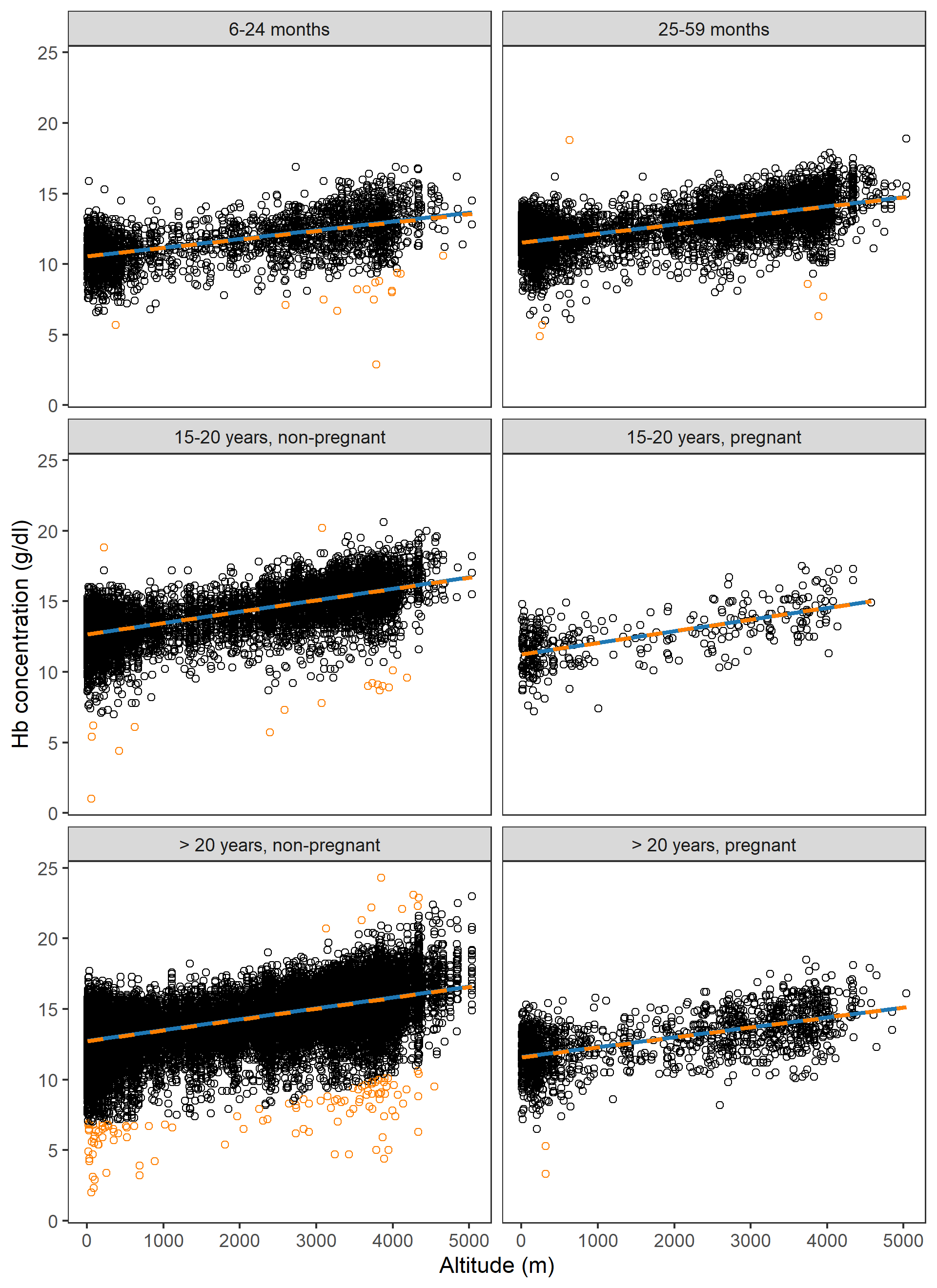


**Figure S2B: South America (SAm)**


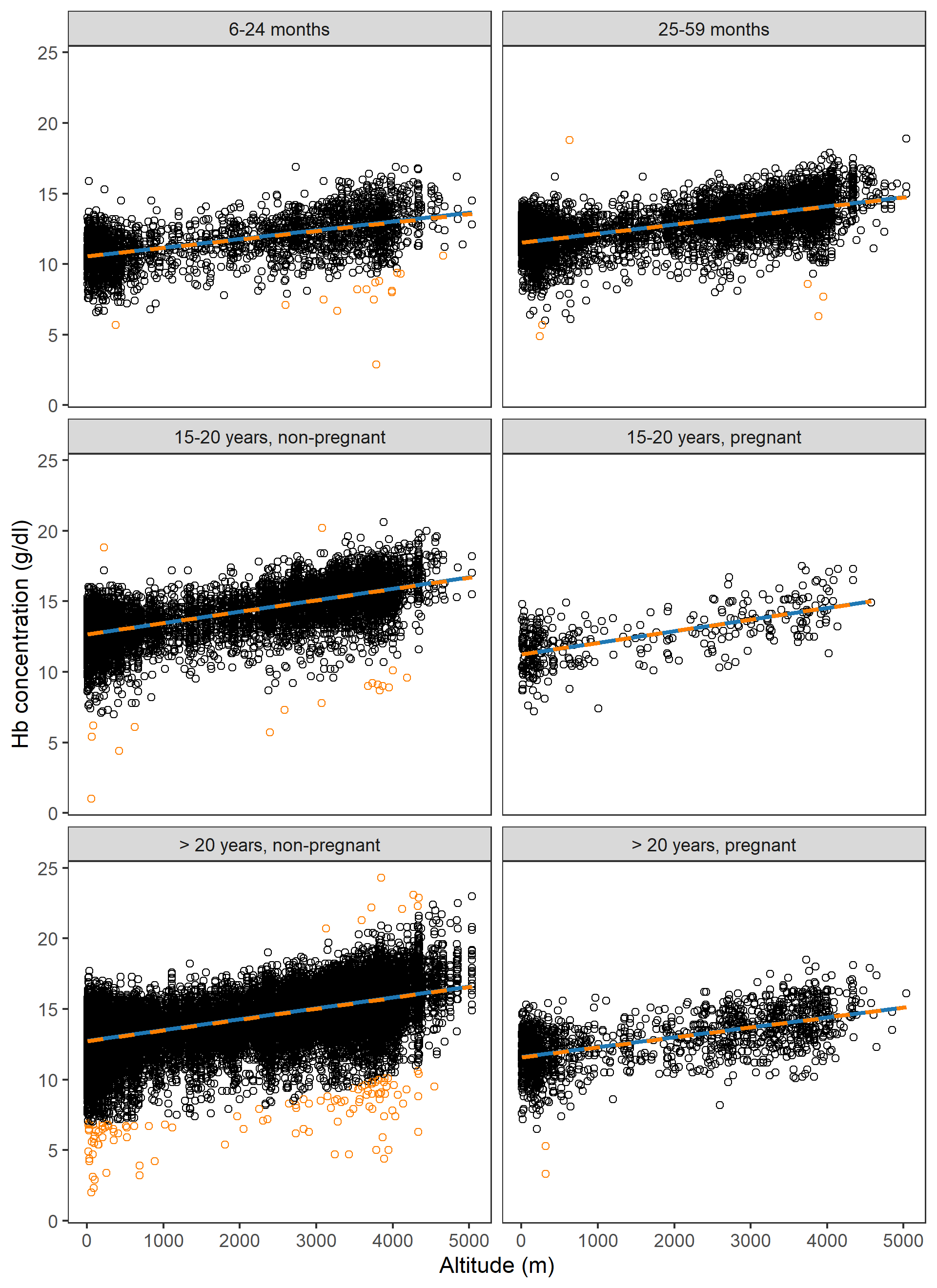


**Figure S3A. Central America: Subgroup-analysis on wealth-index (females)**. Mean values of [Hb] at different altitudes and wealth index (orange = 1 = lowest, yellow = 4, blue = 3, light-green = 4, green = 5 = highest).


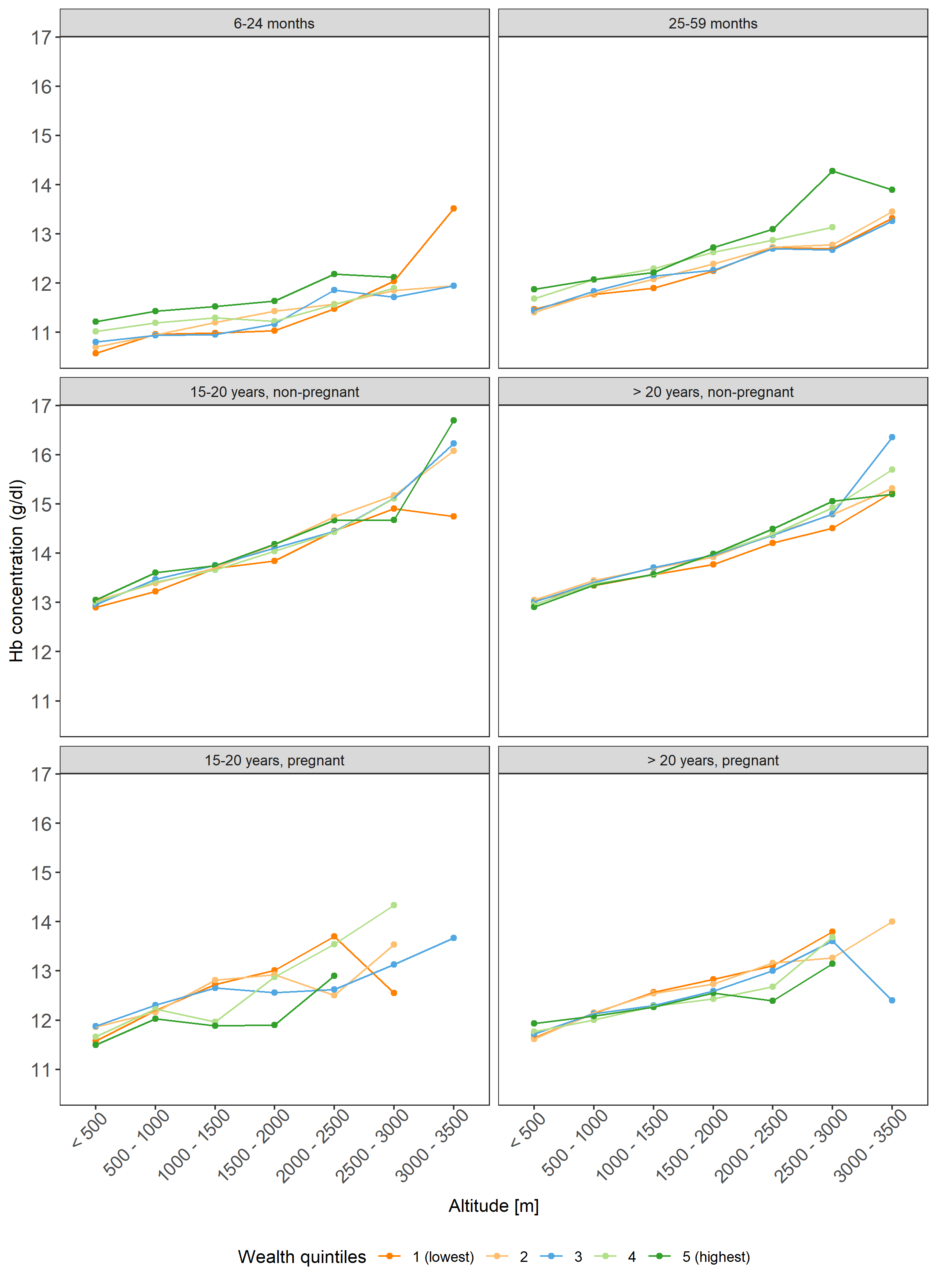


**Figure S3B. South America: Subgroup-analysis on wealth-index (females)**. Mean values of [Hb] at different altitudes and wealth index (orange = 1 = lowest, yellow = 4, blue = 3, light-green = 4, green = 5 = highest).

***
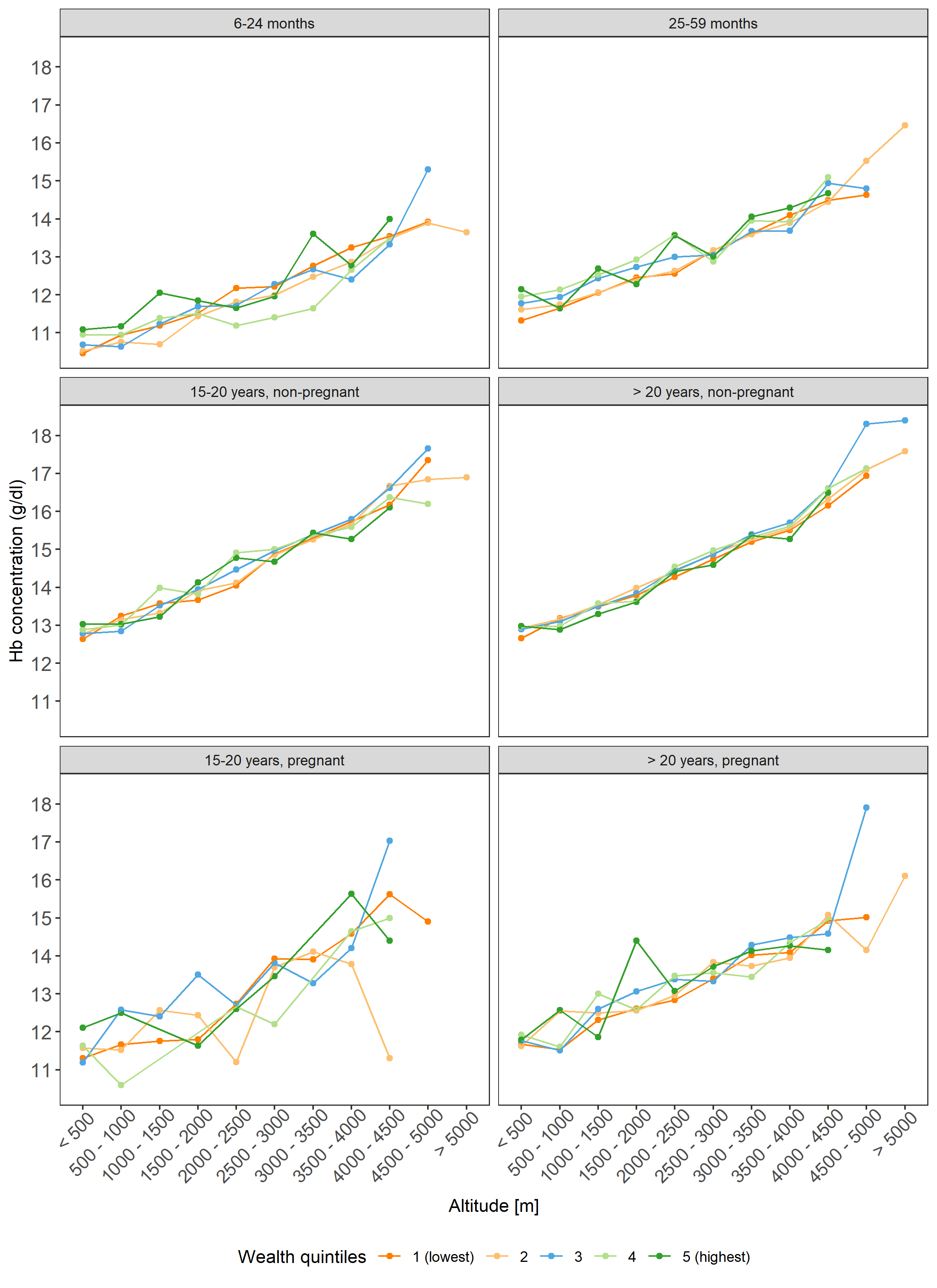
***

**Figure S3C. Western and central Africa: Subgroup-analysis on wealth-index (females)**. Mean values of [Hb] at different altitudes and wealth index (orange = 1 = lowest, yellow = 4, blue = 3, light-green = 4, green = 5 = highest).

***
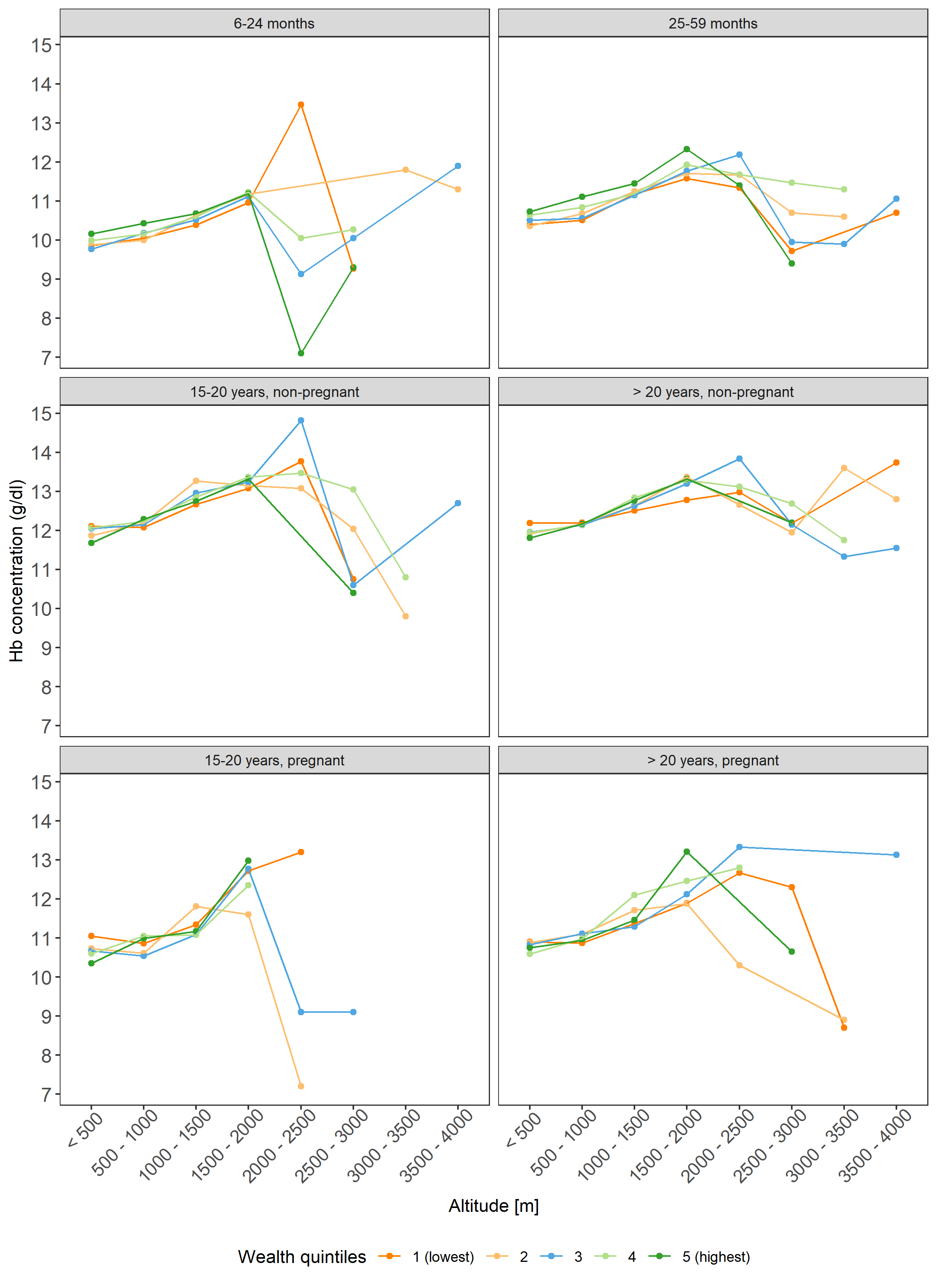
***

**Figure S3D. Southern Africa: Subgroup-analysis on wealth-index (females)**. Mean values of [Hb] at different altitudes and wealth index (orange = 1 = lowest, yellow = 4, blue = 3, light-green = 4, green = 5 = highest).

***
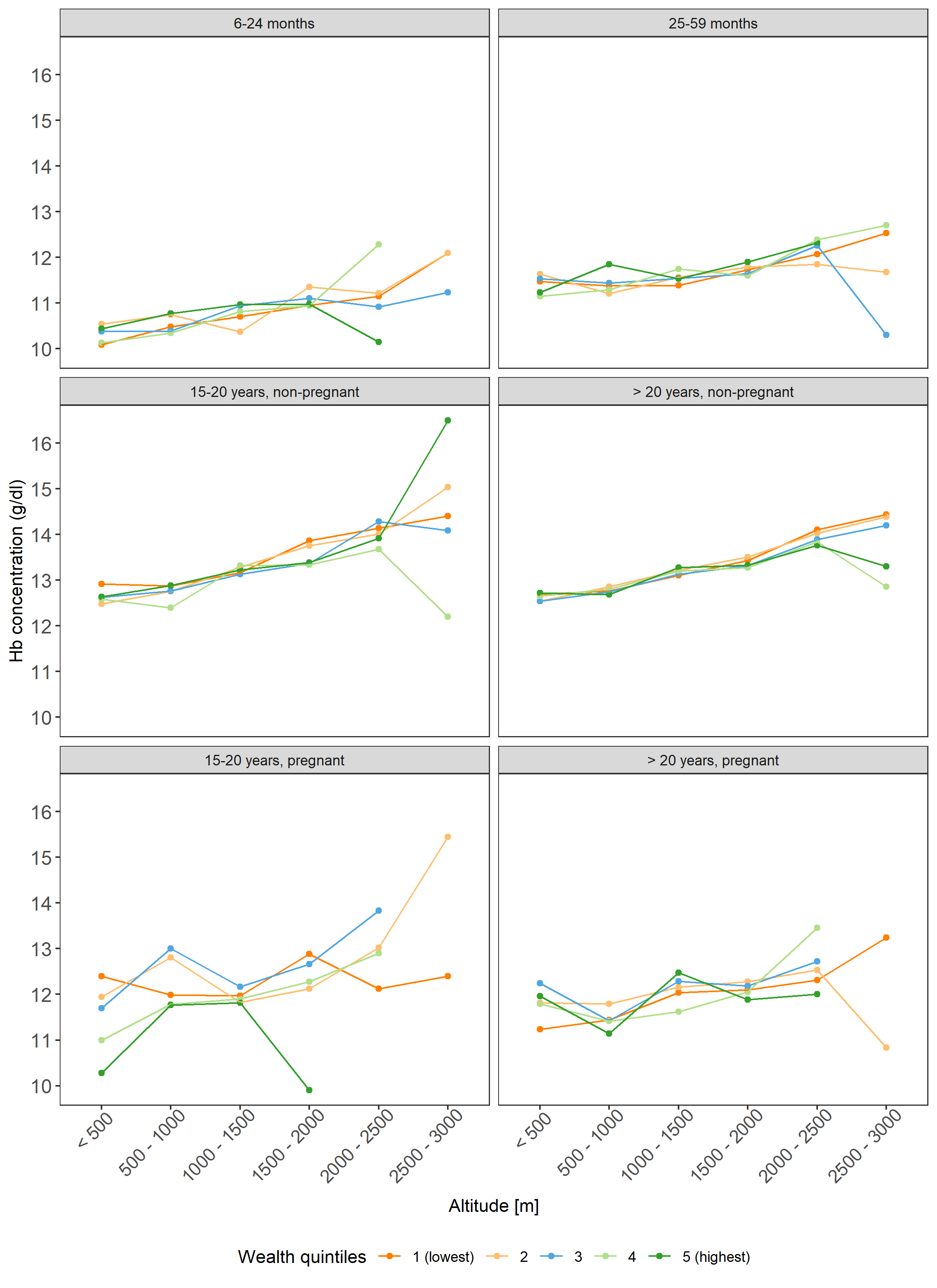
***

**Figure S3E. Eastern Africa: Subgroup-analysis on wealth-index (females)**. Mean values of [Hb] at different altitudes and wealth index (orange = 1 = lowest, yellow = 4, blue = 3, light-green = 4, green = 5 = highest).

***
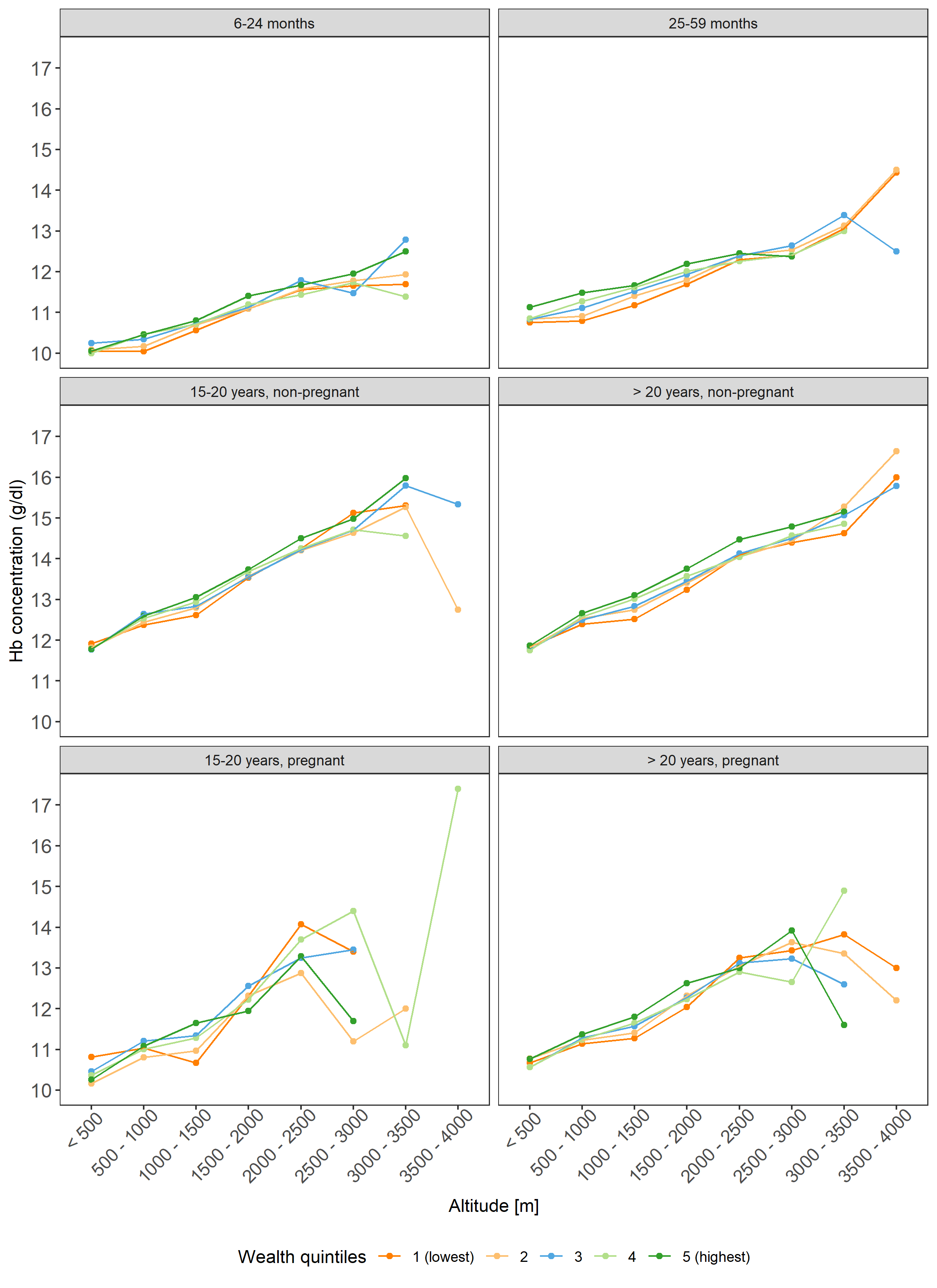
***

**Figure S3F. Middle East: Subgroup-analysis on wealth-index (females)**. Mean values of [Hb] at different altitudes and wealth index (orange = 1 = lowest, yellow = 4, blue = 3, light-green = 4, green = 5 = highest).

***
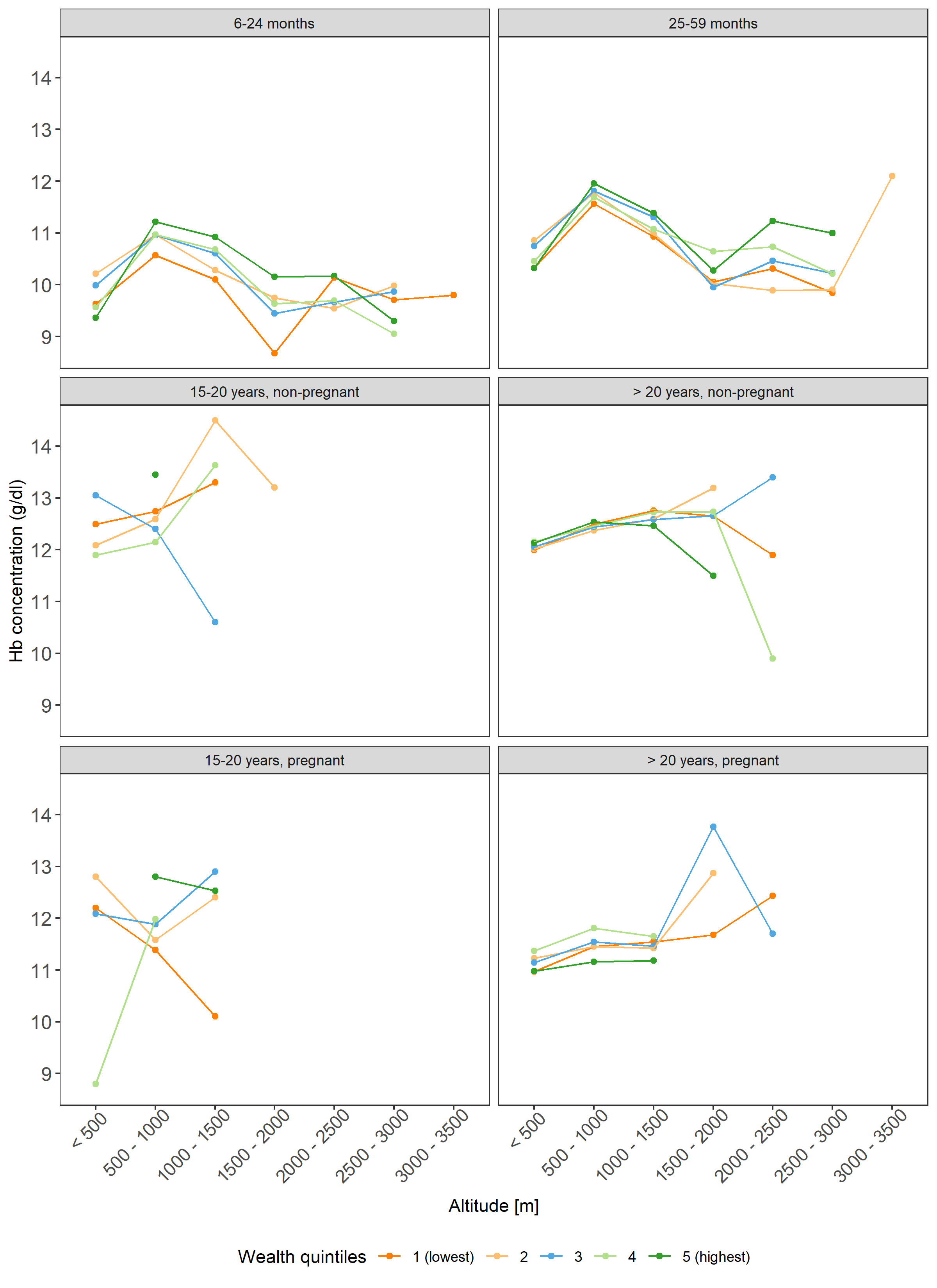
***

**Figure S3G. Central and Western Asia: Subgroup-analysis on wealth-index (females)**. Mean values of [Hb] at different altitudes and wealth index (orange = 1 = lowest, yellow = 4, blue = 3, light-green = 4, green = 5 = highest).

***
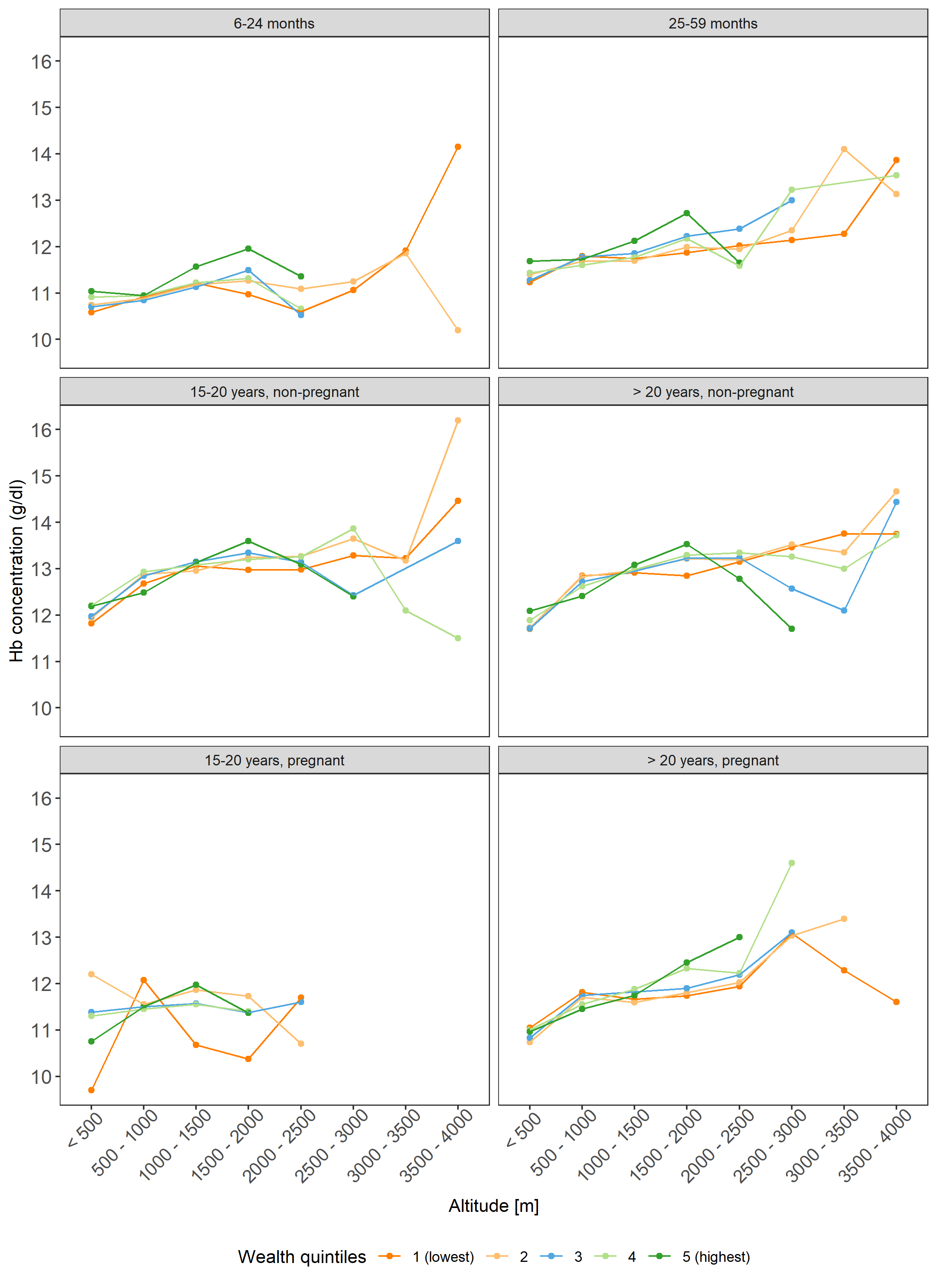
***

**Figure S3H. South/South-East Asia: Subgroup-analysis on wealth-index (females)**. Mean values of [Hb] at different altitudes and wealth index (orange = 1 = lowest, yellow = 4, blue = 3, light-green = 4, green = 5 = highest).

***
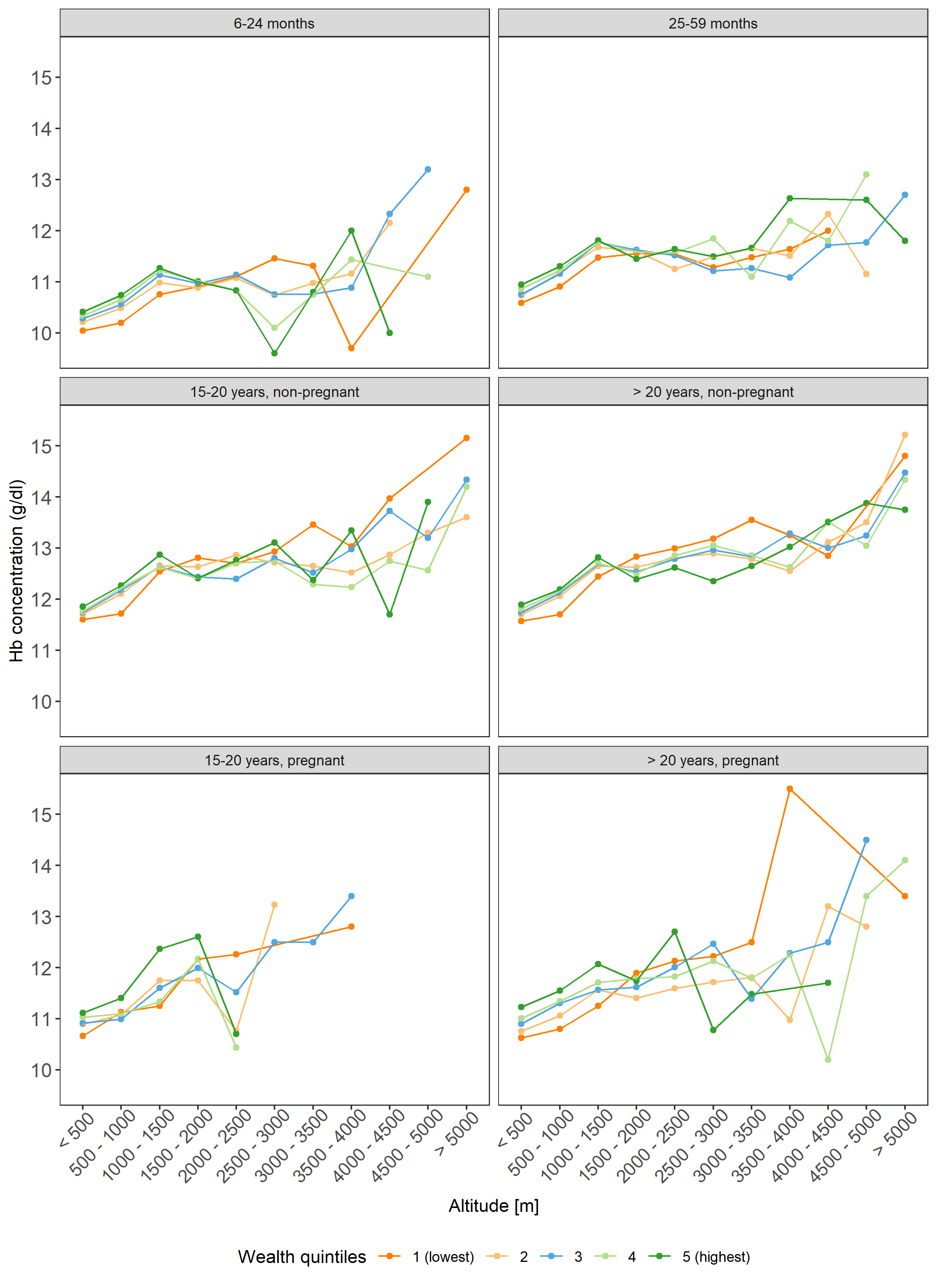
***

**Figure S4. Dependency of differences in the Hb concentration of females at altitude on the region of residence within South/South-East Asia.** The “high regions” of South/South-East Asia represent Nepal and the Indian states of Jammu and Cashmere, Himachal Pradesh, Uttarakhand, Sikkim, and Arunachal Pradesh (orange), which are lined up along the Himalayan mountain-range, and are compared with mean values of Hb from all other regions within South/South-East Asia (blue). Left panel: Dependency of the Hb concentration on altitude in the different age- and pregnancy groups. Right panel: Relative contribution of the number of samples within the high region and the rest of South/South-East Asia in the different altitude-ranges.


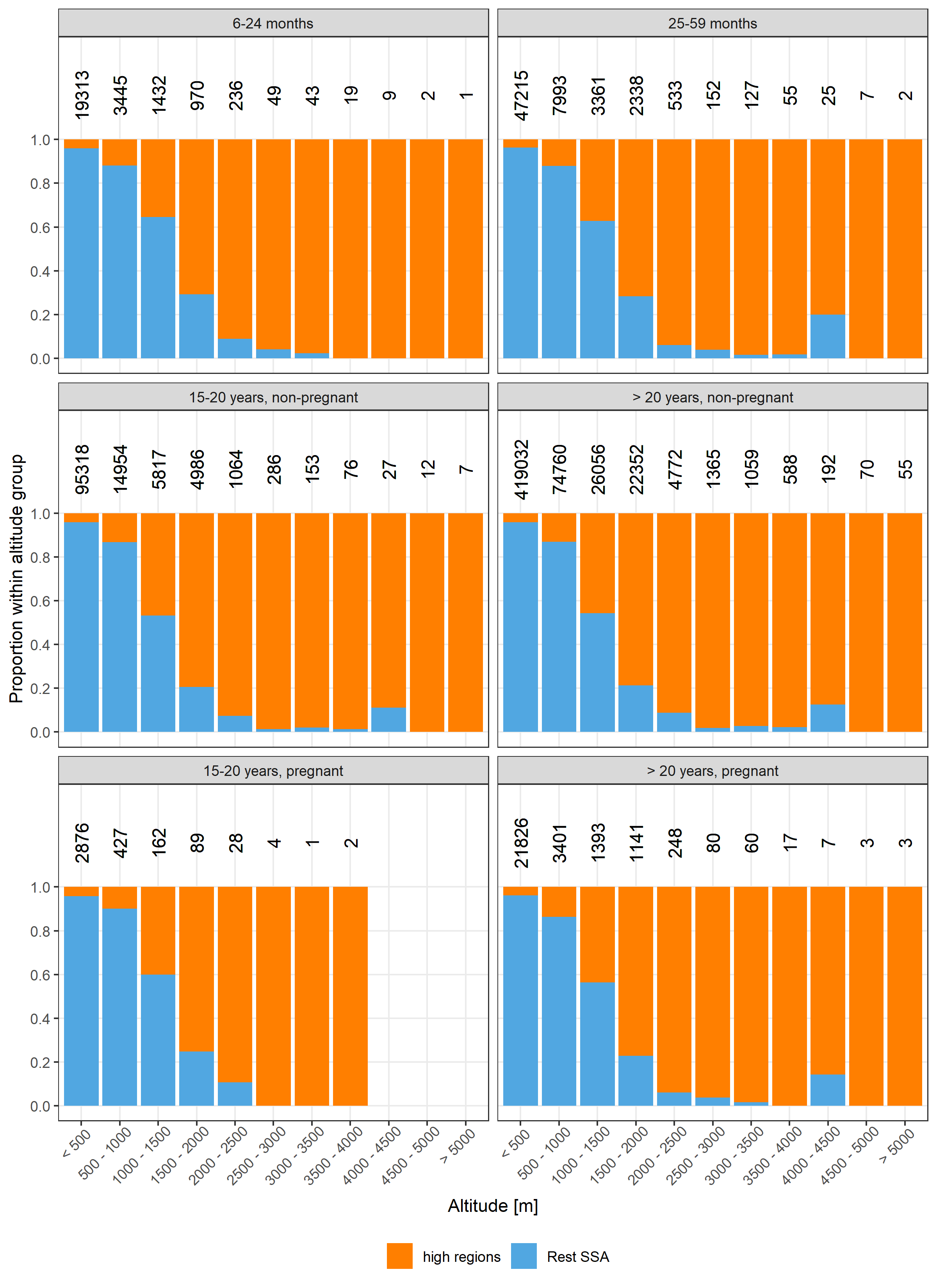

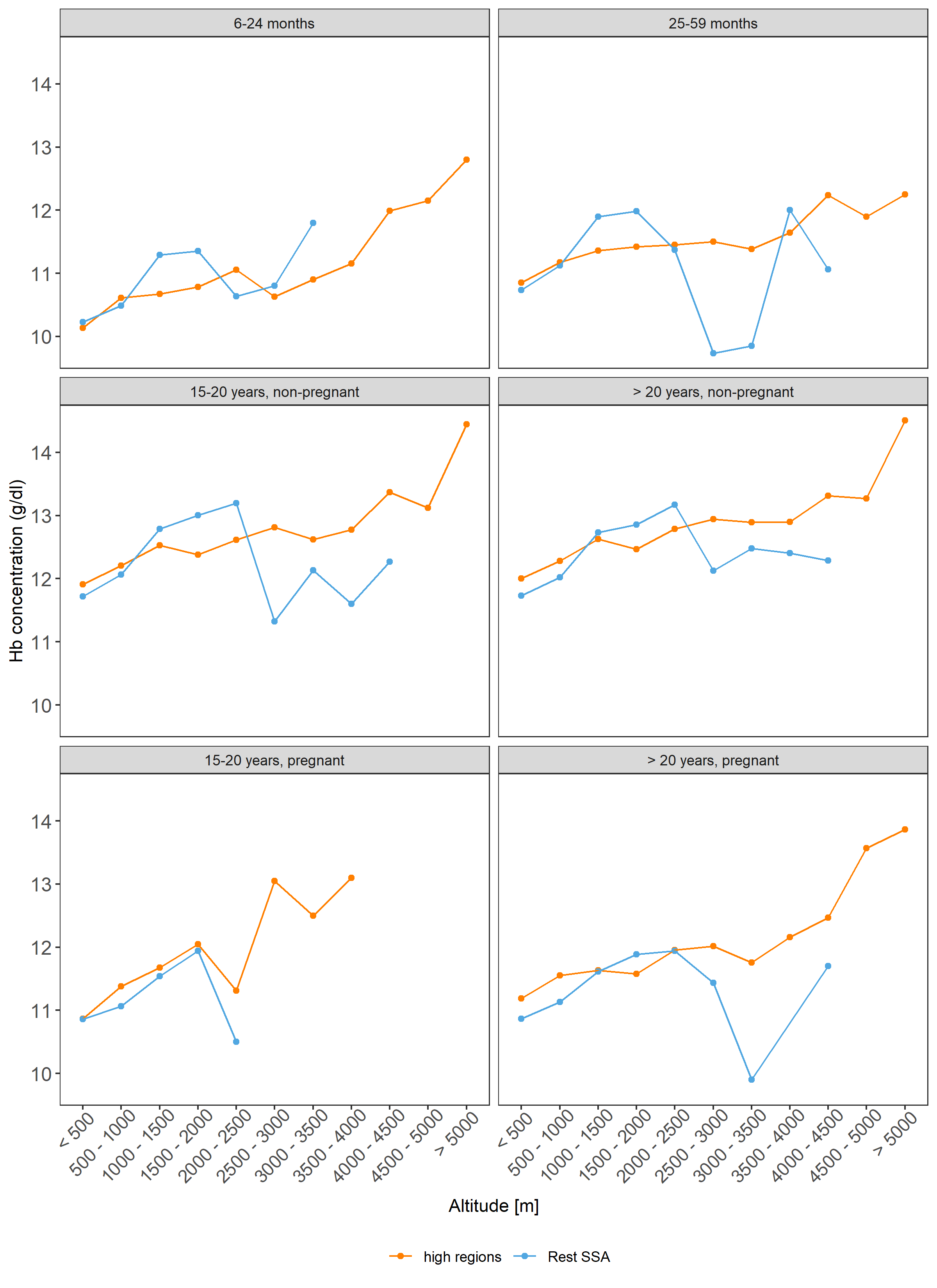


**Figure S5. Comparison of normal ranges for Hb-values for females from South/South-East Asia and South America obtained with different models**.

Each bar represents a reference interval obtained with a different approach: black bars: cut-off values for anemia and polycythemia suggested by the WHO for sea-level and altitude adjustment according to the WHO. Light grey bars: cut-off values for anemia and polycythemia suggested by the WHO for sea-level and altitude adjustment with the delta-Hb/km from our regression analysis. Dark grey bars: lower and upper limit of the reference interval calculated for sea level (shown in table S4) and its altitude adjustment with our linear regressions.

**Figure S5A. Normal ranges for Hb-values for girls age 6 to 59 months from South/South-East Asia**.

**
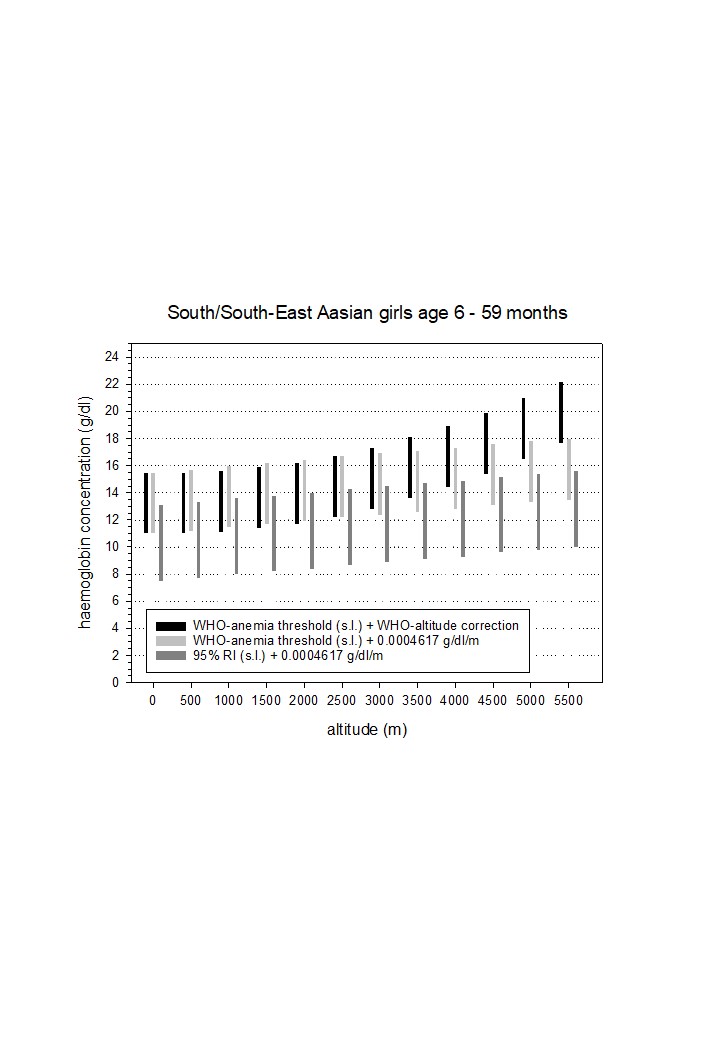
**

**Figure S5B. Normal ranges for Hb-values for girls age 6 to 59 months from South America**.

**
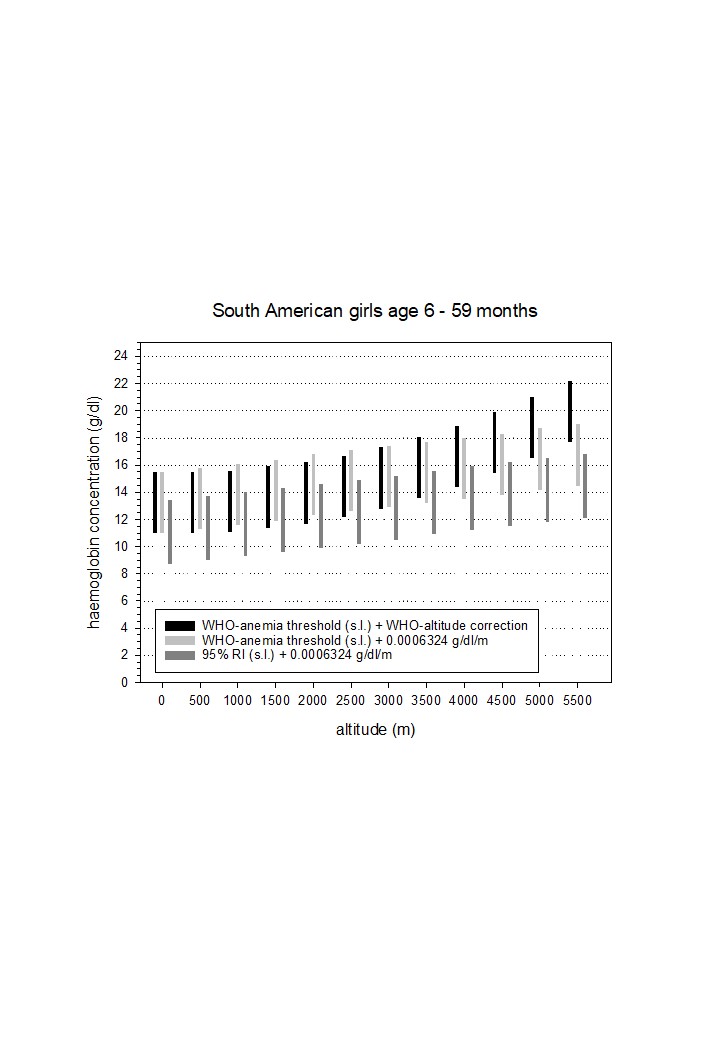
**

**Figure S5C. Normal ranges for Hb-values for pregnant women age > 15 years from South/South-East Asia**.

**
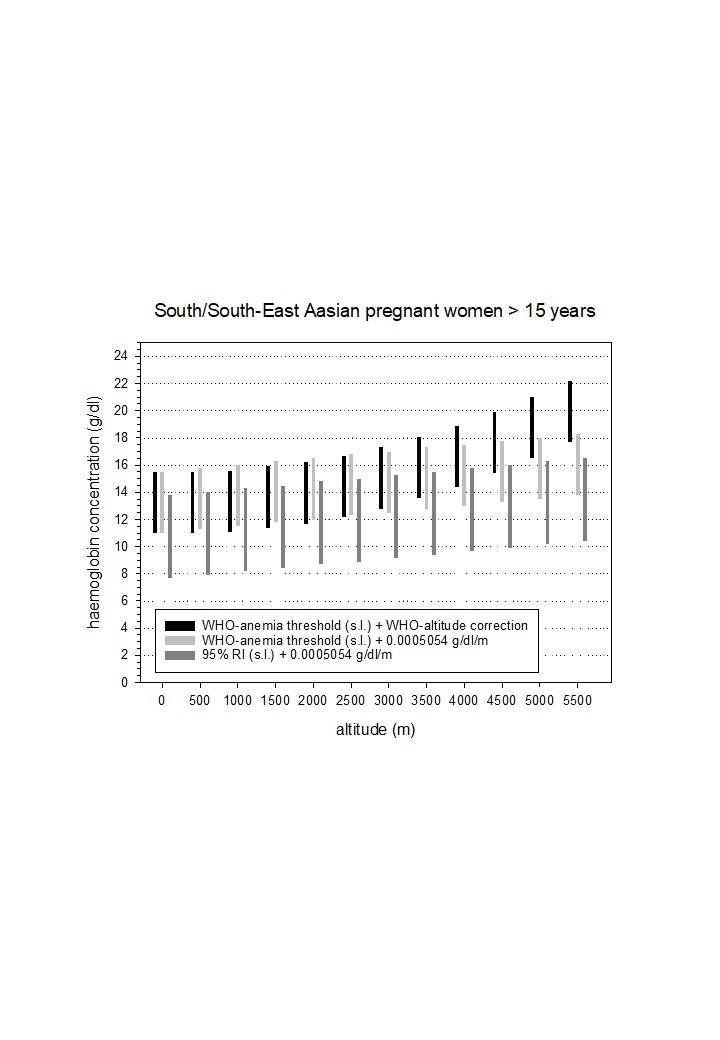
**

**Figure S5D. Normal ranges for Hb-values for pregnant women age > 15 years from South Amerika**

**
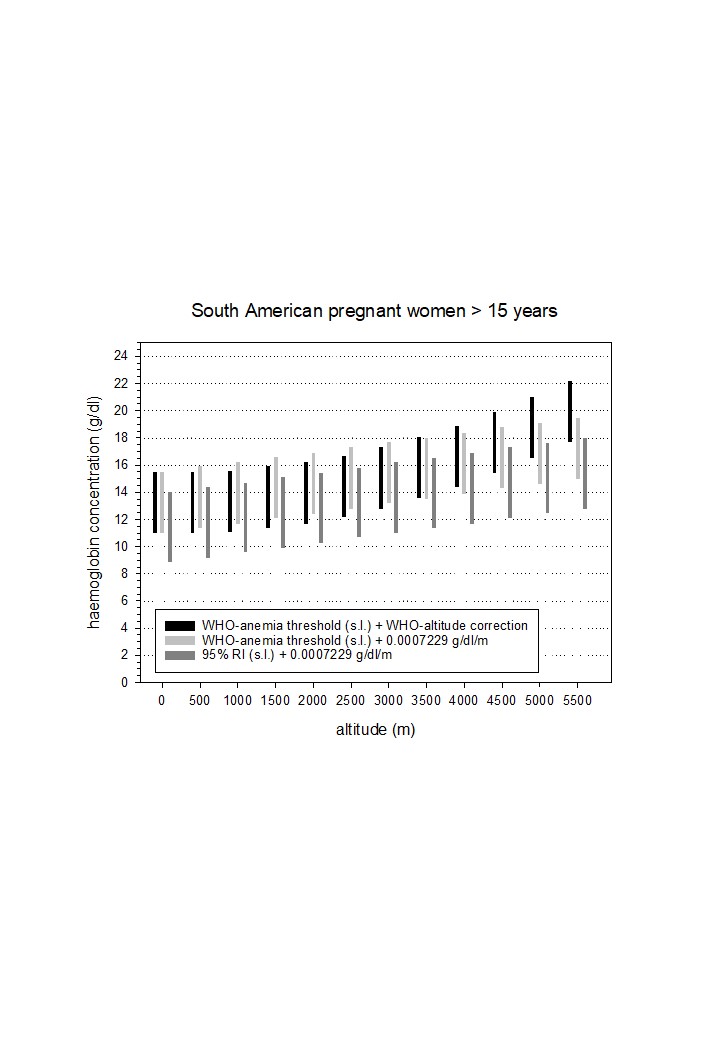
**

**Table S1. Characteristics of sample by world region**

Number of studied individuals in each world region and the percentage within each region by age (both sexes), sex (all ages), pregnancy (percentage relates to the number of all women age >15 years), number of individuals living above 1000 m, and by wealth index.

**Central South Western/Central Southern Eastern Africa Middle East Central/Western South/South- Total**

**America America Africa Africa Asia East Asia**

**n % n % n % n % n % n % n % n % n %**

**Age**

6-24 months 9490 10 5461 8 10114 15 2644 7 28814 12 4206 18 4032 10 52693 5 117454 8

25-59 months 19610 21 12900 19 18834 28 5989 15 58392 24 8586 37 8323 20 127155 13 259789 17

15-20 years 14690 16 9731 14 8303 13 7899 20 35350 15 224 1 5087 12 145857 15 227141 15

>20 years 50163 53 41560 60 29120 44 23508 59 119699 49 9953 43 24851 59 660851 67 959705 61

Total 93953 69652 66371 40040 242255 22969 42293 986556 1564089

**Sex**

male 15018 16 9317 13 21650 33 17272 43 81824 34 6578 29 6394 15 194460 20 352513 23

female 78935 84 60335 87 44721 67 22768 57 160431 66 16391 71 35899 85 792096 80 1211576 77

**Pregnancy** 3607 6 2098 4 3536 12 867 5 9539 8 1151 11 1579 5 31768 5 54145 5

**Altitude >1000 m** 35636 38 33054 48 15548 23 27581 69 163601 68 6301 38 20027 47 103028 10 404776 26

**Wealth index**

1 (lowest) 22533 24 16106 23 13997 21 7943 20 50445 21 6367 28 8260 20 199307 20 324958 21

2 20674 22 16708 24 13586 20 8111 20 43190 18 5987 26 8403 20 213107 22 329766 21

3 18634 20 15126 22 13934 21 8437 21 42596 18 4920 21 8468 20 205747 21 317862 20

4 17255 18 12551 18 13050 20 8244 21 46600 19 3732 16 8199 19 191158 19 300789 19

5 (highest) 14857 16 9161 13 11804 18 7305 18 59424 25 1963 9 8963 21 177237 18 290714 19

**Table S2. Proportion of sample excluded due to abnormally high and low Hb concentration**. n = total number of samples, n-low and n-high, and % low and % high indicate the number and percentage, respectively, of samples, where the Hb concentration was below 7.0 g/dL or above 18.0 g/dL for non-pregnant women, below 6.0 g/dL and 17.0 g/dL for pregnant women and children, and below 9.0 g/dL and 20.0 g/dL for men residing at sea level ^1^, respectively for all world-regions combined. These cut-off values were adjusted to the altitude of residence according to WHO guidance ^3^ to account for the increase in Hb concentration with increasing altitude of residence ^4^. This selection was chosen to exclude samples with implausibly low and high Hb values. Remaining data were used to calculate the linear regressions shown in tables 2 and S?3, and for generating the look-up tables S4.

**Age sex pregn total low high % low % high**

**Males**

6-24 mo m - 60371 535 9 0.89 0.01

25-59 mo m - 133271 652 44 0.49 0.03

15-20 y m - 34884 327 13 0.94 0.04

>20 y m - 126782 1127 88 0.89 0.07

**Females**

6-24 mo f - 58097 450 20 0.77 0.03

25-59 mo f - 127876 627 35 0.49 0.03

15-20 y f - 186852 1465 57 0.78 0.03

15-20 y f p 7293 22 4 0.30 0.05

>20 y f - 794360 6879 252 0.87 0.03

>20 y f p 47159 236 14 0.50 0.03

**Table S3A,B. Regression coefficients describing the change in [Hb] with the residential altitude in children and in non-pregnant and pregnant women (f) from all world regions from all samples obtained at altitudes >1000 m**: Shown are regression coefficients (slope) ± standard error (SE), and the coefficient of determination, R^2^. Table S3B shows the intercepts (int) ± SE, which represent the calculated Hb concentration at sea level. Both were calculated from all data obtained from sea-level up to the extreme altitudes within each region. [Hb] values from boys and girls (bg) were combined because there is no sex-difference in Hb up to an age of ~15 years ^5-7^.Age is shown in months (mo) for the children (boys and girls (bg) combined) and in years (y) for the adult, not-pregnant (np) or pregnant (pr) females (f). The slope (g/dl/km) indicates the change in [Hb] with altitude of residency. R^2^ shown in italic indicate a P-value for the slope of >0.05.

**Table 3A. Change in Hb concentration with altitude (slope; ΔHb/altitude; g/dl/km).**

**World region: CAm* SAm* WCAfr* SAfr* EAfr ME CWA SSEA**

age sex prg slope SE R^2^ slope SE R^2^ slope SE R^2^ slope SE R^2^ slope SE R^2^ slope SE R^2^ slope SE R^2^ slope SE R^2^

6-24 mo bg - 0.492 0.067 0.035 0.758 0.044 0.168 0.752 0.136 0.024 0.540 0.126 0.033 0.894 0.028 0.062 -0.594 0.097 0.025 0.172 0.077 0.003 -0.030 0.041 *0.0001*

25-59 mo bg - 0.598 0.046 0.058 0.801 0.029 0.226 0.643 0.090 0.020 0.444 0.083 0.023 0.860 0.019 0.061 -0.629 0.067 0.025 0.294 0.054 0.010 -0.102 0.026 0.002

15-20 y f np 0.877 0.038 0.109 0.943 0.025 0.265 0.459 0.129 0.015 0.799 0.077 0.022 1.443 0.027 0.151 0.569 3.55 *0.0027* 0.190 0.066 0.004 0.035 0.029 *0.0001*

>20 y f np 0.767 0.021 0.066 0.930 0.012 0.235 0.552 0.072 0.014 0.762 0.042 0.017 1.317 0.015 0.111 0.656 0.253 0.005 0.281 0.030 0.007 0.145 0.012 0.002

15-20 y f pr 0.579 0.151 0.043 1.142 0.131 0.331 0.588 0.462 n.s. 0.799 0.312 0.051 2.102 0.151 0.169 6.695 8.412 *0.265* -0.054 0.582 *0.0002* 0.307 0.218 *0.007*

>20 y f pr 0.646 0.083 0.053 0.817 0.062 0.186 0.610 0.169 0.017 0.396 0.181 0.013 1.486 0.051 0.122 1.626 0.602 *0.049* 0.442 0.122 0.019 0.226 0.056 0.005

**Table 3B. Intercept of regression line representing the Hb concentration at sea level (g/dl).**

**World region: CAm SAm WCAfr SAfr EAfr ME CWA SSEA**

age sex prg int SE int SE int SE int SE int SE int SE int SE int SE

6-24 mo bg - 10.48 0.11 10.10 0.14 9.70 0.19 10.10 0.20 9.50 0.04 10.98 0.16 10.85 0.13 11.02 0.07

25-59 mo bg - 11.35 0.08 11.02 0.09 10.51 0.13 11.00 0.14 10.38 0.03 11.72 0.11 11.48 0.09 11.80 0.04

15-20 y f np 12.61 0.06 12.24 0.08 12.35 0.19 12.20 0.13 11.09 0.05 12.69 4.06 12.84 0.11 12.55 0.05

>20 y f np 12.65 0.03 12.23 0.04 12.07 0.10 12.18 0.07 11.23 0.03 11.87 0.31 12.64 0.05 12.42 0.02

15-20 y f pr 11.83 0.25 10.22 0.41 10.73 0.64 11.01 0.53 8.58 0.22 4.50 9.71 11.52 0.88 11.26 0.34

>20 y f pr 11.61 0.14 11.24 0.19 10.97 0.25 11.54 0.29 9.70 0.08 9.56 0.76 11.21 0.20 11.32 0.10

**Table S4. Look-up tables for Hb concentrations in males and females of different ages and at different altitudes in different regions of the world.** Samples were grouped to fit the respective altitude range; a-mean and a-SD are the mean altitude and standard deviation from the sampling locations, respectively. Hb mean and Hb SD are the mean Hb concentration and SD of the hemoglobin concentration (g/dL) for the respective altitude range. 95% RI lo and RI up are the lower and the upper limit of the 95% reference interval (g/dL), respectively. Preg… pregnancy; mo… months; y… years; n… number of samples; na… not available. Italic print indicates a sample size smaller than 100.

**Central America**

**sex age/preg altitude range a-mean a-SD Hb mean Hb SD 95% RI lo 95% RI up n**

**Male 6-24 mo** <500 m 159 152 10.6 1.2 8.1 12,9 1766

500-1000 m 765 143 10.8 1.3 7.9 13.2 1221

1000-1500 m 1224 145 11.1 1.3 8.3 13.4 954

1500-2000 m 1720 151 11.3 1.3 8.6 13.7 570

2000-2500 m 2228 153 11.7 1.4 8.3 14.1 225

2500-3000 m 2656 109 11.8 1.4 8.8 14.7 108

*3000-3500 m 3228 103 12.4 1.7 9.9 15.5 15*

**Male 25-59 mo** <500 m 160 148 11.5 1.2 9.0 13.8 3754

500-1000 m 762 142 11.8 1.2 9.4 13.9 2567

1000-1500 m 1231 146 12.1 1.1 9.7 14.2 1906

1500-2000 m 1727 151 12.3 1.2 9.9 14.5 1178

2000-2500 m 2239 154 12.8 1.1 10.5 15.0 507

2500-3000 m 2650 113 12.9 1.2 10.6 15.2 219

*3000-3500 m 3207 103 13.1 1.5 9.9 14.9 28*

**Female 6-24 mo** <500 m 163 151 10.8 1.2 8.1 13.1 1732

500-1000 m 764 141 11.0 1.2 8.3 13.3 1162

1000-1500 m 1228 144 11.1 1.2 8.5 13.2 847

1500-2000 m 1731 150 11.2 1.3 8.5 13.5 551

2000-2500 m 2251 151 11.6 1.3 8.9 14.1 234

*2500-3000 m 2657 107 11.9 1.3 9.5 14.0 94*

*3000-3500 m 3190 95 12.7 1.3 11.6 15.1 11*

**Female 25-59 mo** <500 m 161 150 11.6 1.1 9.0 13.6 3506

500-1000 m 758 141 11.8 1.1 9.5 13.9 2335

1000-1500 m 1231 146 12.1 1.1 9.7 14.2 1786

1500-2000 m 1729 150 12.4 1.2 10.1 14.5 1095

2000-2500 m 2229 150 12.8 1.2 10.1 15.1 478

2500-3000 m 2648 99 12.8 1.3 10.1 15.0 229

*3000-3500 m 3192 101 13.4 1.3 10.3 15.2 22*

**Female np 15-20 y** <500 m 154 147 13.0 1.2 10.4 15.3 5417

500-1000 m 767 140 13.4 1.3 10.7 15.7 3301

1000-1500 m 1236 146 13.7 1.2 11.1 16.0 2563

1500-2000 m 1719 149 14.0 1.2 11.6 16.3 1414

2000-2500 m 2250 156 14.5 1.3 11.9 16.9 748

2500-3000 m 2652 104 15.1 1.3 12.2 17.3 349

*3000-3500 m 3222 99 15.9 1.3 13.1 17.7 26*

**Female preg 15-20 y** <500 m 150 144 11.7 1.3 9.3 14.4 356

500-1000 m 757 142 12.2 1.3 9.5 14.5 200

1000-1500 m 1227 136 12.6 1.3 10.3 15.1 165

*1500-2000 m 1694 146 12.8 1.3 10.2 14.9 90*

*2000-2500 m 2235 143 13.0 1.8 9.3 15.8 36*

*2500-3000 m 2669 121 13.5 1.3 9.5 15.7 22*

*3000-3500 m 3176 107 13.7 1.0 10.3 14.5 3*

**Female np >20 y** <500 m 156 149 13.0 1.4 10.0 15.4 18084

500-1000 m 766 141 13.4 1.4 10.4 15.8 11258

1000-1500 m 1232 149 13.6 1.4 10.5 16.1 9340

1500-2000 m 1710 151 13.9 1.4 10.9 16.4 5197

2000-2500 m 2245 155 14.4 1.5 11.0 16.9 2402

2500-3000 m 2651 109 14.8 1.5 11.6 17.4 1051

*3000-3500 m 3217 98 15.7 1.6 12.5 18.3 96*

**Female preg >20 y** <500 m 156 149 11.7 1.3 9.0 14.2 992

500-1000 m 759 142 12.1 1.3 9.5 14.7 666

1000-1500 m 1232 146 12.4 1.3 9.8 14.8 548

1500-2000 m 1731 148 12.7 1.4 9.8 15.4 314

2000-2500 m 2246 149 13.0 1.4 10.0 15.6 133

*2500-3000 m 2656 106 13.5 1.1 11.5 15.6 79*

*3000-3500 m 3247 58 13.5 1.2 12.4 14.6 3*

**South America, children**

**sex age/preg altitude range a-mean a-SD Hb mean Hb SD 95% RI lo 95% RI up n**

**Male 6-24 mo** <500 m 174 128 10.5 1.3 7.7 12.9 1244

500 - 1000 m 689 136 10.7 1.3 8.3 13.3 179

*1000 - 1500 m 1301 133 11.0 1.5 7.8 13.4 81*

*1500 - 2000 m 1789 134 11.2 1.4 8.3 13.5 98*

2000 - 2500 m 2283 149 11.7 1.6 8.6 14.6 157

2500 - 3000 m 2753 128 11.9 1.4 9.0 14.6 234

3000 - 3500 m 3257 136 12.4 1.4 9.4 14.8 289

3500 - 4000 m 3770 130 12.6 1.4 9.5 10.0 377

4000 - 4500 m 4177 141 13.4 1.5 11.0 16.0 113

*4500 - 5000 m 4615 83 13.7 1.5 12.4 15.9 7*

*>5000 m 5037 na 12.7 na na na 1*

**Male 24-59 mo** <500 m 173 128 11.6 1.2 9.1 13.7 2780

500 - 1000 m 684 140 11.8 1.2 9.3 14.0 409

1000 - 1500 m 1273 143 12.0 1.3 9.5 14.3 214

1500 - 2000 m 1769 142 12.5 1.1 10.1 14.4 236

2000 - 2500 m 2278 146 12.7 1.2 10.1 15.1 395

2500 - 3000 m 2749 124 13.0 1.3 10.0 15.2 588

3000 - 3500 m 3261 136 13.5 1.2 11.1 15.7 685

3500 - 4000 m 3760 135 13.9 1.3 11.0 16.2 953

4000 - 4500 m 4158 133 14.6 1.4 11.2 17.1 245

*4500 - 5000 m 4663 119 15.1 1.3 13.1 17.8 29*

*>5000 m 5037 0 16.1 1.6 14.4 17.3 3*

**Female 6-24 mo** <500 m 179 131 10.7 1.2 8.1 13.0 1153

500 - 1000 m 695 142 10.9 1.3 8.2 13.0 183

*1000 - 1500 m 1266 143 11.2 1.2 8.9 13.4 80*

1500 - 2000 m 1786 149 11.6 1.2 8.9 13.9 107

2000 - 2500 m 2307 143 11.8 1.2 9.2 14.3 139

2500 - 3000 m 2740 128 12.0 1.3 9.4 14.1 221

3000 - 3500 m 3260 135 12.6 1.4 9.8 15.3 262

3500 - 4000 m 3768 133 12.9 1.4 10.0 15.6 401

4000 - 4500 m 4171 127 13.5 1.5 10.3 16.7 111

*4500 - 5000 m 4621 131 14.0 1.3 11.3 15.8 22*

*>5000 m 5037 0 13.7 1.2 12.8 14.5 2*

**Female 25-59 mo** <500 m 175 126 11.7 1.1 9.2 13.7 2694

500 - 1000 m 692 140 11.8 1.2 8.9 13.8 406

1000 - 1500 m 1289 136 12.2 1.2 9.8 14.2 230

1500 - 2000 m 1791 155 12.5 1.1 10.0 14.7 216

2000 - 2500 m 2294 142 12.9 1.2 9.9 15.1 381

2500 - 3000 m 2747 133 13.1 1.3 10.3 15.4 574

3000 - 3500 m 3267 135 13.6 1.1 11.1 15.7 694

3500 - 4000 m 3762 134 13.9 1.3 11.0 16.1 866

4000 - 4500 m 4158 135 14.7 1.4 11.8 17.0 261

*4500 - 5000 m 4646 116 14.9 1.2 12.2 16.7 38*

*>5000 m 5037 0 16.5 2.1 15.0 18.7 3*

**South America, adult women**

**sex age/preg altitude range a-mean a-SD Hb mean Hb SD 95% RI lo 95% RI up n**

**Female np 15-20 y** <500 m 148 123 12.8 1.2 10.2 14.9 4201

500 - 1000 m 689 143 13.0 1.2 10.3 15.2 623

1000 - 1500 m 1293 140 13.5 1.2 10.8 15.9 309

1500 - 2000 m 1778 146 13.9 1.2 11.3 16.1 339

2000 - 2500 m 2318 132 14.4 1.3 11.4 16.6 586

2500 - 3000 m 2762 119 14.9 1.3 12.1 17.2 758

3000 - 3500 m 3272 128 15.3 1.3 12.3 17.5 1064

3500 - 4000 m 3747 132 15.7 1.3 12.7 18.0 1123

4000 - 4500 m 4196 140 16.4 1.4 12.9 19.0 325

*4500 - 5000 m 4600 90 17.2 1.4 15.1 19.5 27*

*>5000 m 5037 0 16.9 1.4 15.6 18.1 3*

**Female preg 15-20 y** <500 m 161 115 11.4 1.4 8.7 13.9 178

*500 - 1000 m 709 131 11.9 1.2 10.3 14.0 39*

*1000 - 1500 m 1240 172 12.1 1.9 8.2 13.8 9*

*1500 - 2000 m 1764 161 12.1 1.3 10.2 14.5 20*

*2000 - 2500 m 2356 132 12.5 1.0 11.0 13.9 14*

*2500 - 3000 m 2755 134 13.6 1.6 10.4 16.3 32*

*3000 - 3500 m 3251 148 13.9 1.0 12.4 15.7 26*

*3500 - 4000 m 3754 134 14.4 1.3 12.5 17.2 43*

*4000 - 4500 m 4109 125 15.3 1.9 11.8 17.3 11*

*4500 - 5000 m 4574 na 14.9 na na na 1*

**Female np >20 y** <500 m 146 124 12.9 1.3 9.9 15.1 18575

500 - 1000 m 681 138 13.1 1.4 9.8 15.4 2942

1000 - 1500 m 1286 139 13.5 1.4 10.1 15.9 1381

1500 - 2000 m 1776 149 13.8 1.4 10.6 16.3 1376

2000 - 2500 m 2312 131 14.4 1.4 11.3 16.8 2481

2500 - 3000 m 2758 123 14.8 1.3 11.9 17.1 2903

3000 - 3500 m 3272 129 15.3 1.3 12.3 17.5 4215

3500 - 4000 m 3756 129 15.5 1.5 12.1 18.2 4290

4000 - 4500 m 4212 136 16.4 1.7 12.5 19.7 1438

4500 - 5000 m 4644 112 17.1 1.8 13.6 21.3 210

*>5000 m 5037 0 17.9 1.9 15.1 21.7 24*

**Female preg >20 y** <500 m 155 120 11.7 1.3 9.0 14.1 881

500 - 1000 m 698 143 11.9 1.6 8.7 14.4 111

*1000 - 1500 m 1294 137 12.4 1.3 10.5 14.7 52*

*1500 - 2000 m 1805 153 12.7 1.3 10.4 15.0 47*

*2000 - 2500 m 2296 141 13.1 1.4 10.6 15.5 87*

2500 - 3000 m 2760 121 13.5 1.4 11.1 15.7 137

3000 - 3500 m 3275 138 14.0 1.4 11.2 16.6 154

3500 - 4000 m 3755 134 14.2 1.6 10.6 16.8 194

*4000 - 4500 m 4182 148 14.9 1.5 12.0 17.3 53*

*4500 - 5000 m 4664 121 15.2 1.9 12.5 17.8 8*

*>5000 m 5037 na 16.1 na na na 1*

**West/Central Africa**

**sex age/preg altitude range a-mean a-SD Hb mean Hb SD 95% RI lo 95% RI up n**

**Male 6-24 mo** <500 m 228 167 9.7 1.4 6.8 12.5 2020

500-1000 m 708 129 10.0 1.5 6.9 13.0 1642

1000-1500 m 1193 124 10.4 1.4 7.4 13.0 951

1500-2000 m 1697 111 10.9 1.5 7.8 13.7 443

*2000-2500 m 2105 120 11.3 1.4 8.9 13.7 31*

*2500-3000 m 2667 227 9.9 1.1 8.7 11.5 6*

*3000-3500 m 3463 na 8.6 na na na 1*

*3500-4000 m 3601 0 10.5 0.4 10.1 10.9 4*

**Male 25-59 mo** <500 m 226 168 10.4 1.4 7.3 12.9 3673

500-1000 m 706 129 10.5 1.5 7.2 13.3 2938

1000-1500 m 1193 124 11.2 1.4 8.3 13.6 1800

1500-2000 m 1684 113 11.6 1.4 8.7 14.2 881

*2000-2500 m 2122 120 12.0 1.4 9.2 14.2 37*

*2500-3000 m 2700 224 10.0 1.1 8.0 11.6 17*

*3000-3500 m 3463 0 10.1 0.3 9.6 10.3 4*

*3500-4000 m 3601 0 10.2 1.2 9.0 11.1 3*

**Male 15-20 y** <500 m 362 79 13.3 1.6 10.1 16.4 664

500-1000 m 694 133 13.3 1.7 9.9 16.9 675

1000-1500 m 1200 129 14.1 1.8 11.0 17.7 173

1500-2000 m 1722 142 15.1 1.7 11.7 18.1 124

*2000-2500 m 2029 11 15.4 1.2 14.3 16.9 7*

**Male > 20 y** <500 m 363 78 14.3 1.7 10.8 17.3 2351

500-1000 m 691 126 14.2 1.8 10.5 17.5 2309

1000-1500 m 1206 128 15.0 1.8 11.3 18.1 522

1500-2000 m 1697 140 15.7 1.6 12.2 18.3 366

*2000-2500 m 2026 14 16.0 1.7 12.8 17.3 8*

**Female 6-24 mo** <500 m 236 166 9.9 1.4 7.0 12.4 2027

500-1000 m 707 131 10.1 1.5 7.0 12.8 1590

1000-1500 m 1188 121 10.5 1.4 7.5 13.1 932

1500-2000 m 1689 117 11.1 1.4 8.1 14.0 445

*2000-2500 m 2083 76 10.6 3.0 7.2 15.1 9*

*2500-3000 m 2652 213 9.7 1.3 8.0 11.6 10*

*3000-3500 m 3463 na 11.8 na na na 1*

*3500-4000 m 3601 0 11.6 0.4 11.3 11.9 2*

**Female 25-59 mo** <500 m 231 166 10.5 1.4 7.4 13.0 3768

500-1000 m 711 129 10.7 1.5 7.5 13.4 2888

1000-1500 m 1191 124 11.2 1.4 8.2 13.7 1840

1500-2000 m 1698 109 11.8 1.4 8.7 14.4 907

*2000-2500 m 2087 92 11.8 1.7 8.9 14.8 53*

*2500-3000 m 2754 227 10.2 1.3 8.7 12.9 15*

*3000-3500 m 3463 0 10.6 0.7 9.9 11.3 3*

*3500-4000 m 3601 0 11.0 1.1 9.3 12.3 7*

**Female np 15-20 y** <500 m 262 162 11.9 1.6 8.5 14.8 2441

500-1000 m 702 123 12.2 1.5 8.8 15.1 2283

1000-1500 m 1219 137 12.9 1.5 9.3 15.7 856

1500-2000 m 1690 137 13.2 1.6 9.4 16.0 397

*2000-2500 m 2109 111 13.6 1.5 10.6 16.2 38*

*2500-3000 m 2772 225 11.4 1.3 9.9 13.3 14*

*3000-3500 m 3463 0 10.3 0.7 9.8 10.8 2*

*3500-4000 m 3601 na 12.7 na na na 1*

**Female preg 15-20 y** <500 m 302 138 10.7 1.7 7.6 13.9 239

500-1000 m 700 123 10.8 1.7 7.6 14.0 280

*1000-1500 m 1177 124 11.2 1.9 7.6 14.4 77*

*1500-2000 m 1663 145 12.5 1.7 9.2 14.7 28*

*2000-2500 m 2153 99 9.8 3.1 7.3 13.0 3*

*2500-3000 m 2960 na 9.1 na na na 1*

**Female np >20 y** <500 m 267 159 12.0 1.6 8.5 14.8 8848

500-1000 m 698 121 12.2 1.6 8.7 15.2 7860

1000-1500 m 1205 134 12.7 1.6 9.1 15.6 2580

1500-2000 m 1693 139 13.2 1.7 9.6 16.2 1205

2000-2500 m 2105 97 13.2 1.7 10.2 16.5 101

*2500-3000 m 2726 222 12.3 1.6 9.2 15.2 49*

*3000-3500 m 3463 0 11.8 1.1 10.3 13.4 7*

*3500-4000 m 3601 0 12.9 1.1 11.4 14.0 6*

**Female preg >20 y** <500 m 284 150 10.8 1.6 7.6 13.9 1184

500-1000 m 691 123 11.0 1.7 7.6 14.2 1143

1000-1500 m 1187 133 11.6 1.8 8.0 15.2 362

1500-2000 m 1699 146 12.2 1.7 8.8 15.2 191

*2000-2500 m 2123 123 12.3 1.6 9.6 14.4 18*

*2500-3000 m 2695 242 11.6 1.3 9.8 13.1 5*

*3000-3500 m 3463 0 8.8 0.1 8.7 8.9 2*

*3500-4000 m 3601 0 13.1 1.0 12.1 13.8 3*

**Southern Africa**

**sex age/preg altitude range a-mean a-SD Hb mean Hb SD 95% RI lo 95% RI up n**

**Male 6-24 mo** <500 m 297 148 10.2 1.5 7.2 12.8 222

500-1000 m 779 145 10.4 1.5 7.3 13.3 219

1000-1500 m 1205 140 10.6 1.4 7.6 13.1 408

1500-2000 m 1698 129 11.2 1.6 7.9 14.5 286

2500-3000 m 2262 125 11.4 1.4 8.5 14.1 128

*2500-3000 m 2622 112 11.2 1.6 8.9 13.4 22*

**Male 25-59 mo** <500 m 313 147 11.3 1.5 8.1 14.1 430

500-1000 m 790 149 11.4 1.3 8.3 13.6 480

1000-1500 m 1197 134 11.4 1.4 8.5 14.0 849

1500-2000 m 1699 121 11.7 1.5 8.6 14.4 791

2000-2500 m 2270 132 12.0 1.5 9.2 14.7 365

*2500-3000 m 2625 96 12.2 1.4 9.3 14.9 75*

**Male 15-20 y** <500 m 312 139 14.5 1.6 11.2 17.4 613

500-1000 m 776 146 14.6 1.5 11.6 17.2 622

1000-1500 m 1193 140 14.7 1.7 11.3 17.7 1044

1500-2000 m 1693 120 15.2 1.6 11.7 18.1 1122

2000-2500 m 2243 125 15.3 1.6 12.1 18.4 332

*2500-3000 m 2620 110 15.6 1.5 12.7 17.8 51*

**Male > 20 y** <500 m 236 155 15.0 1.6 11.6 18.0 1484

500-1000 m 784 141 15.1 1.6 11.8 18.0 1307

1000-1500 m 1215 140 15.3 1.7 11.6 18.3 2723

1500-2000 m 1677 116 15.8 1.7 11.8 18.8 2729

2000-2500 m 2264 124 15.9 1.7 11.9 19.0 829

2500-3000 m 2622 106 16.1 1.4 13.2 18.6 141

**Female 6-24 mo** <500 m 309 128 10.3 1.4 7.3 12.8 223

500-1000 m 783 143 10.5 1.4 8.0 13.3 213

1000-1500 m 1198 138 10.7 1.5 8.0 13.5 427

1500-2000 m 1695 122 11.1 1.6 7.5 13.9 345

2000-2500 m 2270 140 11.2 1.6 7.9 14.2 129

*2500-3000 m 2609 104 12.0 1.6 9.5 15.0 22*

**Female 25-59 mo** <500 m 309 143 11.4 1.4 8.4 14.2 409

500-1000 m 789 148 11.4 1.4 8.1 14.0 556

1000-1500 m 1197 134 11.5 1.3 8.7 14.0 844

1500-2000 m 1701 120 11.7 1.5 8.6 14.4 802

2000-2500 m 2266 130 12.0 1.5 9.0 14.7 326

*2500-3000 m 2606 106 12.3 1.4 9.3 15.0 62*

**Female np 15-20 y** <500 m 301 149 12.7 1.5 9.0 15.2 598

500-1000 m 769 143 12.7 1.6 9.0 15.5 632

1000-1500 m 1197 140 13.2 1.6 9.7 16.0 996

1500-2000 m 1693 120 13.5 1.6 9.7 16.3 1221

2000-2500 m 2259 124 14.1 1.6 10.3 16.6 416

*2500-3000 m 2622 114 14.4 1.6 10.4 17.0 55*

**Female preg 15-20 y** *<500 m 339 142 11.8 1.7 8.3 14.2 31*

*500-1000 m 790 148 12.3 1.5 9.8 15.4 32*

*1000-1500 m 1204 134 12.0 1.6 9.4 16.0 59*

*1500-2000 m 1698 128 12.4 1.5 10.0 14.9 44*

*2000-2500 m 2259 140 12.7 1.6 10.3 15.3 27*

*2500-3000 m 2569 50 13.9 1.8 12.0 15.5 4*

**Female np >20 y** <500 m 254 162 12.6 1.7 8.7 15.5 2045

500-1000 m 771 143 12.8 1.7 8.8 15.8 2113

1000-1500 m 1214 143 13.2 1.7 9.3 16.1 3743

1500-2000 m 1677 119 13.3 1.8 9.2 16.3 4187

2000-2500 m 2263 128 14.0 1.6 10.5 16.8 1276

2500-3000 m 2622 103 14.4 1.5 11.2 16.9 230

**Female preg >20 y** <500 m 266 165 11.8 1.5 9.0 15.0 122

500-1000 m 769 154 11.5 1.9 8.1 15.2 108

1000-1500 m 1204 138 12.1 1.4 9.1 14.7 214

1500-2000 m 1679 117 12.1 1.4 9.3 14.6 186

*2000-2500 m 2270 122 12.6 1.4 10.2 15.5 59*

*2500-3000 m 2667 111 12.6 1.5 9.9 14.4 12*

**Eastern Africa males**

**sex age/preg altitude range a-mean a-SD Hb mean Hb SD 95% RI lo 95% RI up n**

**Male 6-24 mo** <500 m 170 166 9.9 1.4 6.9 12.6 3166

500-1000 m 774 141 10.1 1.4 7.0 12.7 2127

1000-1500 m 1245 137 10.5 1.4 7.4 13.2 4949

1500-2000 m 1718 136 11.0 1.4 7.9 13.5 2890

2000-2500 m 2213 149 11.5 1.4 8.4 14.3 982

2500-3000 m 2656 122 11.5 1.6 8.4 14.1 190

*3000-3500 m 3149 132 12.1 1.6 9.3 14.4 33*

**Male 25-59 mo** <500 m 169 164 10.8 1.4 7.7 13.3 6744

500-1000 m 774 144 11.0 1.5 7.7 13.6 4441

1000-1500 m 1245 137 11.4 1.4 8.3 14.0 9870

1500-2000 m 1721 137 11.8 1.4 8.6 14.3 5850

2000-2500 m 2217 150 12.3 1.4 9.1 14.8 2064

2500-3000 m 2662 119 12.6 1.5 9.0 15.2 468

*3000-3500 m 3161 129 13.2 1.3 10.9 15.7 72*

*3500-4000 m 3563 0 13.6 0.6 13.1 14.0 2*

**Male 15-20 y** <500 m 405 74 13.8 1.6 10.5 16.8 542

500-1000 m 819 136 14.0 1.7 10.5 17.2 1400

1000-1500 m 1228 136 14.5 1.7 10.9 17.6 4068

1500-2000 m 1747 147 15.1 1.7 11.5 18.1 1729

2000-2500 m 2262 155 15.7 1.7 12.3 18.7 1335

2500-3000 m 2662 115 15.7 1.7 12.2 18.8 400

*3000-3500 m 3151 115 15.9 1.5 13.2 18.6 69*

*3500-4000 m 3563 0 15.9 0.9 15.0 17.3 6*

**Male > 20 y** <500 m 404 79 14.7 1.7 10.8 17.7 1627

500-1000 m 796 146 14.7 1.7 10.9 17.8 3471

1000-1500 m 1245 139 15.2 1.7 11.5 18.3 11380

1500-2000 m 1749 145 15.7 1.7 11.7 18.8 5804

2000-2500 m 2263 152 16.3 1.7 12.6 19.3 4637

2500-3000 m 2657 121 16.5 1.7 13.0 19.5 1294

3000-3500 m 3182 137 16.4 1.6 13.3 19.2 196

*3500-4000 m 3563 0 17.1 1.5 14.0 18.9 18*

**Eastern Africa females**

**Female 6-24 mo** <500 m 161 164 10.1 1.3 7.2 12.5 3262

500-1000 m 774 143 10.3 1.4 7.1 12.9 2095

1000-1500 m 1243 137 10.7 1.4 7.9 13.2 5088

1500-2000 m 1716 136 11.2 1.4 8.1 13.6 2819

2000-2500 m 2218 150 11.6 1.4 8.6 14.2 974

2500-3000 m 2678 137 11.7 1.5 8.2 14.2 207

*3000-3500 m 3140 112 11.9 1.6 9.1 14.0 32*

**Female 25-59 mo** <500 m 170 165 10.9 1.4 7.8 13.3 6388

500-1000 m 773 143 11.1 1.5 7.7 13.6 4197

1000-1500 m 1244 137 11.5 1.4 8.3 14.0 9886

1500-2000 m 1720 138 11.9 1.4 8.7 14.4 5816

2000-2500 m 2216 152 12.4 1.4 9.3 14.8 2103

2500-3000 m 2680 127 12.5 1.5 9.1 14.9 422

*3000-3500 m 3175 150 13.1 1.5 10.0 16.0 64*

*3500-4000 m 3563 0 14.1 0.9 12.6 14.8 5*

**Female np 15-20 y** <500 m 159 173 11.8 1.6 8.3 14.8 4674

500-1000 m 763 145 12.5 1.6 9.0 15.4 3247

1000-1500 m 1255 138 12.9 1.6 9.5 15.7 8084

1500-2000 m 1720 139 13.6 1.5 10.3 16.3 5155

2000-2500 m 2246 151 14.3 1.4 11.1 16.8 2601

2500-3000 m 2655 116 14.9 1.5 11.4 17.5 564

*3000-3500 m 3201 147 15.3 1.4 12.2 17.7 65*

*3500-4000 m 3563 0 14.3 2.8 10.6 17.2 5*

**Female preg 15-20 y** <500 m 178 166 10.4 1.6 6.8 13.4 346

500-1000 m 739 145 11.0 1.8 7.5 14.7 300

1000-1500 m 1233 130 11.1 1.7 7.7 14.1 518

1500-2000 m 1698 130 12.3 1.8 8.8 15.1 176

*2000-2500 m 2202 144 13.5 1.6 9.6 15.6 55*

*2500-3000 m 2678 136 13.2 1.5 11.3 15.1 8*

*3000-3500 m 3042 16 11.6 0.6 11.1 12.0 2*

*3500-4000 m 3563 na 17.4 na na na 1*

**Female np >20 y** <500 m 169 174 11.8 1.6 8.2 14.8 16352

500-1000 m 764 147 12.5 1.7 8.8 15.5 11403

1000-1500 m 1260 140 12.9 1.7 9.1 15.9 27789

1500-2000 m 1717 139 13.5 1.6 9.7 16.3 17619

2000-2500 m 2243 151 14.2 1.4 11.0 16.8 8005

2500-3000 m 2650 119 14.6 1.5 11.2 17.2 1717

3000-3500 m 3188 145 15.0 1.4 12.3 17.3 236

*3500-4000 m 3563 0 16.1 1.4 13.6 18.7 18*

**Female preg >20 y** <500 m 175 172 10.7 1.6 7.3 13.5 1591

500-1000 m 751 150 11.2 1.6 7.9 14.3 1281

1000-1500 m 1254 138 11.5 1.7 8.0 14.5 2830

1500-2000 m 1710 135 12.3 1.7 8.6 15.3 1667

2000-2500 m 2221 155 13.0 1.5 10.0 15.9 620

2500-3000 m 2651 112 13.4 1.5 10.1 16.0 122

*3000-3500 m 3150 123 13.6 1.5 11.2 16.1 20*

*3500-4000 m 3563 0 12.6 0.6 12.2 13.0 2*

**Middle East**

**sex age/preg altitude range a-mean a-SD Hb mean Hb SD 95% RI lo 95% RI up n**

**Male 6-24 mo** <500 m 68 253 9.8 1.7 6.3 12.8 303

500-1000 m 747 129 10.9 1.5 7.6 13.4 1130

1000-1500 m 1207 138 10.3 1.9 6.6 13.6 440

1500-2000 m 1748 172 9.3 1.5 6.8 12.4 139

2000-2500 m 2232 146 9.9 1.7 7.2 13.5 156

*2500-3000 m 2598 79 9.7 1.7 7.3 12.5 13*

*3000-3500 m 3091 na 9.9 na na na 1*

**Male 25-59 mo** <500 m 101 243 10.5 1.8 6.7 13.5 715

500-1000 m 743 126 11.7 1.5 8.0 14.1 2194

1000-1500 m 1197 136 11.0 1.9 7.0 14.2 812

1500-2000 m 1741 175 10.2 1.9 7.1 13.7 313

2000-2500 m 2241 133 10.5 1.6 7.6 13.4 305

*2500-3000 m 2605 64 10.9 1.7 7.9 13.9 51*

*3000-3500 m 3091 0 10.0 1.4 8.7 12.2 6*

**Female 6-24 mo** <500 m 98 229 9.8 1.8 6.5 12.9 313

500-1000 m 737 129 10.9 1.4 7.5 13.2 1019

1000-1500 m 1190 137 10.4 1.8 7.0 13.3 393

1500-2000 m 1739 175 9.5 1.6 6.9 13.0 143

2000-2500 m 2223 134 9.8 1.6 7.1 12.9 126

*2500-3000 m 2601 72 9.7 1.5 7.8 12.9 29*

*3000-3500 m 3091 na 9.8 na na na 1*

**Female 25-59 mo** <500 m 99 236 10.5 1.8 6.7 13.6 655

500-1000 m 739 127 11.7 1.5 8.1 14.0 2118

1000-1500 m 1200 136 11.1 2.0 6.9 14.4 815

1500-2000 m 1754 176 10.1 1.7 7.1 13.4 287

2000-2500 m 2211 135 10.6 1.8 7.2 13.6 261

*2500-3000 m 2620 80 10.1 1.5 7.7 13.2 53*

*3000-3500 m 3091 na 12.1 na na na 1*

**Female np 15-20 y** *<500 m 71 268 12.5 1.0 10.8 14.4 24*

500-1000 m 728 122 12.6 1.2 10.4 14.7 119

*1000-1500 m 1111 61 13.3 1.4 10.8 15.2 15*

*1500-2000 m 1564 na 13.2 na na na 1*

**Female preg 15-20 y** *<500 m 240 215 11.8 1.7 8.9 13.8 9*

500-1000 m 722 112 11.7 1.2 9.4 13.7 49

*1000-1500 m 1149 85 12.2 1.1 10.3 13.3 7*

**Female np >20 y** <500 m 66 265 12.1 1.5 8.9 14.8 1288

500-1000 m 742 130 12.4 1.5 8.9 15.0 5858

1000-1500 m 1176 132 12.6 1.5 9.1 15.2 1593

1500-2000 m 1541 32 12.8 1.5 9.1 15.3 122

*2000-2500 m 2002 0 12.1 2.1 10.0 15.4 6*

**Female preg >20 y** <500 m 70 283 11.1 1.3 8.8 13.6 160

500-1000 m 736 126 11.5 1.4 8.6 14.1 714

1000-1500 m 1193 137 11.5 1.5 8.6 14.2 186

*1500-2000 m 1552 42 12.3 1.2 10.0 14.1 22*

*2000-2500 m 2002 0 12.3 0.8 11.7 13.3 4*

**Central/Western Asia**

**sex age/preg altitude range a-mean a-SD Hb mean Hb SD 95% RI lo 95% RI up n**

**Male 6-24 mo** <500 m 233 182 10.5 1.3 7.7 12.7 454

500-1000 m 802 120 11.1 1.4 8.0 13.6 799

1000-1500 m 1234 139 10.9 1.4 7.8 13.4 434

1500-2000 m 1738 132 11.2 1.4 8.4 13.6 283

2000-2500 m 2150 130 11.0 1.8 7.3 14.1 111

*2500-3000 m 2804 165 11.0 1.4 9.1 13.1 18*

*3000-3500 m 3252 127 11.9 1.3 10.2 13.7 9*

*3500-4000 m 3790 123 11.8 1.6 10.0 13.3 4*

**Male 25-59 mo** <500 m 232 182 11.3 1.3 8.5 13.6 933

500-1000 m 797 118 11.7 1.2 9.0 13.8 1606

1000-1500 m 1231 140 11.8 1.4 8.5 14.2 863

1500-2000 m 1716 130 12.0 1.4 9.0 14.5 604

2000-2500 m 2140 123 12.0 1.5 8.6 14.6 211

*2500-3000 m 2784 124 11.8 1.1 9.3 13.2 40*

*3000-3500 m 3219 111 12.6 1.2 10.3 14.3 15*

*3500-4000 m 3757 122 12.5 1.4 10.4 14.2 10*

**Female 6-24 mo** <500 233 178 10.8 1.3 7.9 13.2 437

500-1000 m 796 115 10.9 1.3 7.9 13.0 714

1000-1500 m 1229 147 11.2 1.4 8.3 13.7 393

1500-2000 m 1727 133 11.3 1.5 7.8 14.0 259

*2000-2500 m 2147 126 10.8 1.8 7.6 13.4 80*

*2500-3000 m 2800 106 11.1 1.1 9.4 13.1 20*

*3000-3500 m 3274 115 11.9 1.1 9.6 13.5 14*

*3500-4000 m 3788 150 12.8 2.4 10.4 14.8 3*

**Female 25-59 mo** <500 m 259 176 11.4 1.2 8.8 13.6 858

500-1000 m 796 122 11.7 1.3 8.9 14.1 1523

1000-1500 m 1225 142 11.8 1.3 8.9 14.0 834

1500-2000 m 1735 132 12.1 1.3 9.0 14.5 549

2000-2500 m 2137 125 12.0 1.6 8.3 14.7 191

*2500-3000 m 2767 133 12.3 1.3 9.9 14.7 65*

*3000-3500 m 3248 98 12.5 1.4 10.4 14.2 9*

*3500-4000 m 3706 110 13.6 1.5 11.1 15.4 12*

**Female np 15-20 y** <500 m 405 39 12.1 1.3 8.9 14.3 463

500-1000 m 807 119 12.7 1.3 9.8 15.2 2047

1000-1500 m 1230 140 13.1 1.3 9.8 15.3 1248

1500-2000 m 1734 136 13.2 1.4 10.2 15.9 798

2000-2500 m 2139 110 13.1 1.5 9.4 15.7 315

*2500-3000 m 2722 141 13.3 1.3 11.3 16.1 47*

*3000-3500 m 3249 103 13.2 1.3 11.0 15.3 20*

*3500-4000 m 3713 103 14.4 1.7 11.7 16.7 12*

**Female preg 15-20 y** *<500 m 397 39 11.1 1.3 8.3 12.3 14*

*500-1000 m 778 135 11.6 1.5 8.4 14.1 68*

*1000-1500 m 1221 141 11.6 1.1 9.1 13.1 31*

*1500-2000 m 1738 117 11.3 1.3 8.7 13.0 19*

*2000-2500 m 2141 62 11.3 1.3 10.0 13.2 5*

**Female np >20 y** <500 m 400 39 11.9 1.4 8.7 14.4 2162

500-1000 m 814 114 12.6 1.5 9.2 15.2 9382

1000-1500 m 1227 141 13.0 1.5 9.6 15.5 5869

1500-2000 m 1733 140 13.2 1.5 9.3 15.7 4091

2000-2500 m 2130 109 13.1 1.6 9.3 16.0 1523

2500-3000 m 2749 136 13.4 1.6 10.1 16.1 232

*3000-3500 m 3185 100 13.7 1.3 11.2 16.0 92*

*3500-4000 m 3707 109 13.9 2.1 10.1 17.4 58*

**Female preg >20 y** <500 m 403 41 10.9 1.4 8.1 13.6 189

500-1000 m 800 124 11.6 1.4 8.7 14.3 617

1000-1500 m 1225 140 11.7 1.5 8.8 14.3 342

1500-2000 m 1727 132 12.0 1.5 8.1 14.8 201

*2000-2500 m 2151 145 12.1 1.7 8.9 15.3 60*

*2500-3000 m 2799 148 13.1 1.0 11.3 14.5 20*

*3000-3500 m 3191 102 12.4 1.1 10.7 14.3 12*

*3500-4000 m 3796 na 11.6 na na na 1*

**South/South-East Asia, males**

**sex age/preg altitude range a-mean a-SD Hb mean Hb SD 95% RI lo 95% RI up n**

**Male 6-24 mo** <500 m 184 131 10.1 1.4 7.2 12,9 20605

500-1000 m 688 135 10.4 1.5 7.3 13.2 3639

1000-1500 m 1243 146 10.9 1.5 8.0 13.6 1502

1500-2000 m 1689 134 10.9 1.5 8.0 13.9 1039

2000-2500 m 2192 143 11.0 1.6 7.9 14.1 220

*2500-3000 m 2775 149 10.6 1.5 8.3 13.7 69*

*3000-3500 m 3231 97 10.6 1.3 8.3 13.2 57*

*3500-4000 m 3704 156 11.1 1.4 9.2 13.9 32*

*4000-4500 m 4288 150 11.0 0.7 10.19.6 12.2 10*

*4500-5000 m 4741 na 12.6 na na na 1*

**Male 24-59 mo** <500 m 185 131 10.8 1.4 7.8 13.3 50256

500-1000 m 686 133 11.1 1.4 8.2 13.6 8426

1000-1500 m 1245 145 11.7 1.3 8.8 14.2 3388

1500-2000 m 1687 128 11.6 1.5 8.5 14.4 2399

2000-2500 m 2183 134 11.6 1.5 8.3 14.5 502

2500-3000 m 2778 140 11.5 1.4 8.3 13.8 152

3000-3500 m 3224 102 11.3 1.4 8.9 14.0 119

*3500-4000 m 3715 153 11.5 1.5 9.1 14.5 65*

*4000-4500 m 4188 139 12.1 1.4 9.9 14.7 29*

*4500-5000 m 4736 111 12.1 0.9 11.2 13.5 5*

*>5000 m 5097 126 12.3 0.4 11.7 12.6 6*

**Male 15-20 y** <500 m 187 131 13.7 1.6 10.4 16.7 14635

500-1000 m 676 134 14.0 1.6 10.6 17.0 2452

1000-1500 m 1245 157 14.4 1.6 11.0 17.5 1090

1500-2000 m 1695 126 14.5 1.7 11.0 17.5 1021

2000-2500 m 2180 140 14.6 1.6 11.4 17.6 222

*2500-3000 m 2754 144 14.6 1.6 12.0 17.7 71*

*3000-3500 m 3219 103 14.9 1.6 11.6 17.4 40*

*3500-4000 m 3673 118 14.7 1.7 12.4 17.5 18*

*4000-4500 m 4342 158 15.0 1.2 13.7 17.4 16*

*4500-5000 m 4617 123 14.8 0.9 14.2 15.7 3*

**Male >20 y** <500 m 187 133 14.2 1.6 10.5 17.2 62428

500-1000 m 676 130 14.4 1.7 10.8 17.5 10527

1000-1500 m 1238 153 14.8 1.6 11.2 17.8 3653

1500-2000 m 1687 123 15.0 1.7 11.3 18.1 4145

2000-2500 m 2162 127 15.1 1.7 11.5 18.2 863

2500-3000 m 2759 143 15.4 1.6 12.0 18.3 312

3000-3500 m 3244 112 15.7 1.6 12.8 18.9 219

3500-4000 m 3706 133 15.6 1.5 13.1 18.9 121

*4000-4500 m 4199 165 16.2 1.6 13.6 19.4 36*

*4500-5000 m 4725 87 15.5 0.9 14.2 17.0 29*

*>5000 m 5218 193 16.6 1.1 15.1 18.9 38*

**South/South-East Asia, females**

**sex age/preg altitude range a-mean a-SD Hb mean Hb SD 95% RI lo 95% RI up n**

**Female 6-24 mo** <500 m 184 131 10.2 1.4 7.3 13.0 19313

500-1000 m 690 134 10.5 1.4 7.7 13.2 3445

1000-1500 m 1241 144 11.1 1.5 8.0 14.0 1432

1500-2000 m 1688 132 11.0 1.5 8.0 13.9 970

2000-2500 m 2184 136 11.0 1.4 8.4 14.2 236

*2500-3000 m 2785 139 10.6 1.5 8.0 13.5 49*

*3000-3500 m 3231 107 10.9 1.2 8.9 13.3 43*

*3500-4000 m 3686 158 11.2 1.5 9.2 13.7 19*

*4000-4500 m 4182 129 12.0 1.2 10.1 13.6 9*

*4500-5000 m 4762 42 12.2 1.5 11.2 13.1 2*

*>5000 m 5020 NA 12.8 na na na 1*

**Female 25-59 mo** <500 m 185 132 10.7 1.4 7.8 13.2 47215

500-1000 m 688 133 11.1 1.3 8.2 13.6 7993

1000-1500 m 1245 144 11.7 1.3 8.8 14.1 3361

1500-2000 m 1688 129 11.6 1.4 8.4 14.2 2338

2000-2500 m 2179 136 11.4 1.5 8.3 14.3 533

2500-3000 m 2791 138 11.4 1.5 8.6 14.1 152

3000-3500 m 3230 112 11.4 1.3 9.1 13.9 127

*3500-4000 m 3657 104 11.7 1.2 9.2 13.6 55*

*4000-4500 m 4308 141 12.0 1.0 10.4 13.6 25*

*4500-5000 m 4693 102 11.9 0.8 11.1 13.0 7*

*>5000 m 5149 142 12.3 0.6 11.8 12.7 2*

**Female np 15-20 y** <500 m 184 128 11.7 1.5 8.5 14.3 95318

500-1000 m 684 132 12.1 1.5 8.8 14.8 14954

1000-1500 m 1250 149 12.7 1.5 9.4 15.4 5817

1500-2000 m 1686 129 12.5 1.6 9.0 15.4 4986

2000-2500 m 2177 136 12.7 1.6 9.2 15.6 1064

2500-3000 m 2796 146 12.8 1.7 9.2 15.8 286

3000-3500 m 3232 113 12.6 1.6 9.4 15.2 153

*3500-4000 m 3699 149 12.8 1.5 10.2 15.6 76*

*4000-4500 m 4223 148 13.2 1.4 11.4 15.9 27*

*4500-5000 m 4731 90 13.1 0.7 12.3 14.3 12*

*>5000 m 5248 201 14.4 0.8 13.6 15.8 7*

**Female preg 15-20 y** <500 m 175 133 10.9 1.5 7.7 13.8 2876

500-1000 m 673 129 11.1 1.5 7.8 13.8 427

1000-1500 m 1236 147 11.6 1.5 8.3 14.1 162

*1500-2000 m 1676 136 12.0 1.5 8.8 14.6 89*

*2000-2500 m 2219 127 11.2 2.0 8.3 14.5 28*

*2500-3000 m 2784 145 13.1 1.1 12.2 14.6 4*

*3000-3500 m 3282 0 12.5 na na na 1*

*3500-4000 m 3563 0 13.1 0.4 12.8 13.4 2*

**Female np >20 y** <500 m 184 131 11.7 1.5 8.4 14.4 419032

500-1000 m 687 132 12.1 1.6 8.6 14.8 74760

1000-1500 m 1246 148 12.7 1.6 9.1 15.4 26056

1500-2000 m 1684 127 12.5 1.7 8.8 15.6 22352

2000-2500 m 2180 139 12.8 1.6 9.4 15.9 4772

2500-3000 m 2783 146 12.9 1.7 9.3 16.2 1365

3000-3500 m 3246 117 12.9 1.7 9.6 16.3 1059

3500-4000 m 3711 145 12.9 1.6 10.2 16.2 588

4000-4500 m 4253 165 13.2 1.4 11.1 16.6 192

*4500-5000 m 4726 94 13.3 1.0 11.9 15.5 70*

*>5000 m 5207 178 14.5 1.1 12.7 16.8 55*

**Female preg >20 y** <500 m 186 128 10.9 1.5 7.6 13.7 21826

500-1000 m 686 134 11.2 1.6 7.9 14.1 3401

1000-1500 m 1247 144 11.6 1.6 8.3 14.4 1393

1500-2000 m 1692 128 11.6 1.7 8.1 14.6 1141

2000-2500 m 2175 133 12.0 1.8 87.9 15.1 248

*2500-3000 m 2789 152 12.0 1.6 8.4 15.2 80*

*3000-3500 m 3250 111 11.7 1.9 8.5 15.0 60*

*3500-4000 m 3700 152 12.2 1.8 9.2 15.5 17*

*4000-4500 m 4206 206 12.4 1.9 10.2 14.6 7*

*4500-5000 m 4778 41 13.6 0.9 12.8 14.4 3*

*>5000 m 5165 161 13.9 1.0 13.2 14.9 3*

**Table S5**. **Prevalence of anemia in boys and girls age 6 to 59 month, and in not pregnant and pregnant women older than 15 years in different world regions; comparison of methods of evaluation**. The table shows the total number of individuals (N), the number of anemic individuals (n), and percent-anemics. cut-off s.l. is the threshold for anemia suggested by the WHO for sea level (boys, girls and pregnant women: 11 g/dl; non-pregnant women >15 years: 12 g/dl) ^2^, and the lower level of the 95% confidence interval (95% CI-lo for residents living below 500 m above sea level. Δ altitude indicates the method used for altitude-adjustment: WHO: altitude correction by the method suggested by the WHO ^2^; regression: slope of the linear regression fitting the respective set of datapoints for the respective group. Age-groups according to the WHO ^2^ rather than more detailed grouping shown in figure 2 because of low numbers in some age-subgroups. Values are shown for N > 50.

**6 - 59 mo (boys and girls) women > 15 y, not pregnant women > 15 y, pregnant**

cut-off, s.l.: **WHO WHO <95% CI-lo WHO WHO <95% CI-lo WHO WHO <95% CI-lo**

Δ altitude: **WHO regression regression WHO regression regression WHO regression regression**

**N n/% n/% n/% N n/% n/% n/% N n/% n/% n/%**

**Anemia in Central American people (CAm)** (no data on males >15y)

<500 10761 3971/36.9 5041/37.4 367/3.4 23523 4559/19.4 6709/28.5 908/3.9 1349 360/26.7 493/36.6 73/5.4

500-999 7261 2374/32.7 3286/45.3 254/3.5 14511 2143/14.8 3803/26.2 463/3.2 862 164/19.0 297/34.5 41/4.8

1000-1499 5488 1908/34.8 2571/46.9 214/3.9 11902 1765/14.8 3564/29.9 421/3.5 713 134/18.8 253/35.5 33/4.6

1500-1999 3400 1256/36.9 1594/46.9 126/3.7 6623 1020/15.4 2025/30.6 234/3,5 405 87/21.5 142/35.1 25/6.2

2000-2499 1450 574/39.5 608/41.9 48/3.3 3161 518/16.4 907/28.7 128/4,1 170 46/27.1 66/38.8 16/9.4

2500-2999 650 349/53.7 292/44.9 20/3.1 1404 283/20.2 358/25.5 51/3,6 101 24/23.8 28/27.7 3/3.0

3000-3499 76 47/61.8 31/40.8 5/6.6 122 30/24.6 22/18.0 3/2,5

**Anemia in South American people (SAm)** (no data on males >15y)

<500 7880 2719/34.5 3709/47.1 317/4.0 22816 4536/19.9 6761/29.6 802/3.5 1061 300/28.3 399/37.6 33/3.1

500-999 1178 396/33.6 611/51.8 57/4.8 3574 694/19.4 1317/36.9 186/5.2 150 45/30.0 59/39.3 9/6.0

1000-1499 606 186/30.7 312/51.5 30/5.0 1692 291/17.2 592/35.0 86/5.1 61 12/19.7 22/36.1 2/3.3

1500-1999 654 204/31.2 328/50.2 30/4.6 1717 312/18.2 645/37.6 97/5.7 67 23/34.3 34/50.8 1/1.5

2000-2499 1074 387/36.0 507/47.2 51/4.8 3072 452/14.7 1000/32.6 128/4.2 101 27/26.7 44/43.6 2/2.0

2500-2999 1621 814/50.2 814/50.2 100/6.2 3670 686/18.7 1208/32.9 141/3.8 169 51/30.2 62/36.7 6/3.6

3000-3499 1934 1106/57.2 816/42.2 67/3.5 5295 1304/24.6 1567/29.6 181/3.4 180 70/38.9 64/35.6 7/3.9

3500-3999 2632 1828/69.5 1170/44.5 180/6.8 5453 2244/41.2 1924/35.3 296/5.4 237 130/54.9 101/42.6 9/3.8

4000-4499 735 541/73.6 262/35.7 28/3.8 1778 818/46.0 491/27.6 89/5.0 64 36/56.3 18/43.0 2/3.1

4500-4999 98 91/92.9 30/30.6 5/5.1 238 141/59.2 49/20.6 8/3.4

**6 - 59 mo (boys and girls) women > 15 y, not pregnant women > 15 y, pregnant**

cut-off, s.l.: **WHO WHO <95% CI-lo WHO WHO <95% CI-lo WHO WHO <95% CI-lo**

Δ altitude: **WHO regression regression WHO regression regression WHO regression regression**

**N n/% n/% n/% N n/% n/% n/% N n/% n/% n/%**

**Anemia in Central and Western African people (CWAfr)**

<500 11585 7845/67.7 8974/77.5 442/3.8 12809 6538/51.0 7829/61.1 437/3,4 1428 771/54.0 893/62.5 54/3.8

500-999 9155 5973/65.5 7287/79.6 469/5.1 11633 5628/48.4 7702/66.2 459/4,0 1429 736/51.5 1000/70.0 102/7.1

1000-1499 5575 3323/59.6 4372/78.4 202/3.6 3894 1573/40.4 2356/60.5 155/4,0 442 202/45.7 283/64.0 29/6.7

1500-1999 2697 1426/52.9 1955/72.5 81/3.0 1836 691/37.6 1094/59.6 71/3,9 221 78/35.3 121/54.8 12/5.4

2000-2499 131 78/59.5 94/71.8 7/5.3 163 79/48.5 108/66.3 7/4,3

2500-2999 56 55/98.2 55/98.2 19/3.9 70 62/88.6 64/91.4 13/18,6

**Anemia in Southern African people (SAfr)**

<500 1291 594/46.0 710/55.0 40/3.1 2657 779/29.3 995/37.5 92/3.5 153 41/26.8 54/35.3 8/5.3

500-999 1467 684/46.6 840/57.3 38/2.6 2757 822/29.8 1205/43.7 115/4.2 140 52/37.1 61/43.6 15/10.7

1000-1499 2535 1316/51.9 1544/60.9 57/2.3 4763 1295/27.2 1945/40.8 162/3.4 274 88/32.1 107/39.1 13/4.7

1500-1999 2236 1142/51.1 1250/55.9 73/3.3 5448 1640/30.1 2476/45.5 267/4.9 230 76/33.0 89/38.7 14/6.1

2000-2499 956 555/58.1 529/55.3 21/2.2 1698 449/26.4 624/36.8 39/2.3 86 33/38.4 31/36.1 0/0.0

2500-2999 181 116/64.1 94/51.9 3/1.7 285 97/34.0 109/38.3 3/1.1

**Anemia in East African people (EAfr)**

<500 19633 11516/58.7 13760/70.1 684/3.5 21290 10975/51.6 14329/67.3 884/4.0 1961 1104/56.3 1398/71.3 82/4.2

500-999 12908 7159/55.5 9241/71.6 604/4.7 14717 5346/36.3 9343/63.5 479/3.3 1588 723/45.5 1158/72.9 61/3.8

1000-1499 29946 15782/52.7 21612/72.2 1230/4.1 36160 12713/35.2 25003/69.2 1476/4.1 3371 1555/46.1 2559/75.9 197/5.8

1500-1999 17512 8502/48.6 12084/69.0 705/4.0 22873 5974/26.1 15729/68.8 801/3.5 1858 609/32.8 1313/70.7 88/4.7

2000-2499 6171 3105/50.3 4143/67.1 212/3.4 10645 2129/20.0 7171/67.4 219/2.1 677 181/26.7 474/70.0 15/2.2

2500-2999 1303 792/60.8 882/67.7 89/6.8 2297 574/25.0 1659/72.2 69/3.0 130 35/26.9 100/76.9 4/3.1

3000-3499 207 145/70.1 137/66.2 14/6.8 302 96/31.8 228/75.5 5/1.7

**Anemia in Middle East people (ME)**

<500 2018 1214/60.2 1179/58.4 34/1.7 1313 568/43.3 686/52.3 45/3.4 168 74/44.1 83/49.4 10/6.0

500-999 6456 2296/35.6 1725/26.7 17/0.3 5967 2132/35.7 2927/49.1 245/4.1 763 274/35.9 354/46.4 40/5.2

1000-1499 2501 1429/57.1 1021/40.8 12/.05 1601 612/38.2 796/49.7 73/4.6 190 80/42.1 99/55.1 17/9.0

1500-1999 933 764/81.9 590/63.2 2/0.2 124 46/37.1 60/48.4 5/4.0

2000-2499 893 769/86.1 420/47.0 1/0.1

2500-2999 163 148/90.8 77/47.2 0/0.0

**6 - 59 mo (boys and girls) women > 15 y, not pregnant women > 15 y, pregnant**

cut-off, s.l.: **WHO WHO <95% CI-lo WHO WHO <95% CI-lo WHO WHO <95% CI-lo**

Δ altitude: **WHO regression regression WHO regression regression WHO regression regression**

**N n/% n/% n/% N n/% n/% n/% N n/% n/% n/%**

**Anemia in Central and West Asian people (CWA)**

<500 2659 1098/41.3 1277/48.0 82/3.1 2644 1260/47.7 1532/57.9 89/3.4 203 95/46.8 117/57.6 7/3.5

500-999 4646 1577/33.9 2039/43.9 118/2.5 11448 3355/29.3 5053/44.1 255/2.2 683 219/32.1 297/43.5 16/2.3

1000-1499 2528 1054/41.7 1179/46.6 109/4.3 7135 2089/29.3 3026/42.4 181/2.5 374 136/36.4 184/49.2 14/3.7

1500-1999 1707 713/41.8 713/41.8 62/3.6 4935 1609/32.6 2244/45.5 195/4.0 221 89/40.3 125/56.6 15/6.8

2000-2499 600 356/59.3 301/50.2 60/10.0 1867 857/45.9 1026/55.0 101/5.4 65 34/52.3 38/58.5 4/6.2

2500-2999 144 112/77.8 66/45.8 4/2.8 283 169/59.7 169/59.7 11/3.9

3000-3499 113 82/72.6 72/63.7 3/2.7

3500-3999 73 53/72.6 40/54.8 8/11.0

**Anemia in South-South/East Asian people (SSEA)**

<500 133236 78406/58.9 90620/68.0 4551/3.4 508660 271746/53.4 316056/62.1 15397/3.0 24207 12338/51.0 14313/59.1 772/3.2

500-999 21936 11361/51.8 14053/64.1 589/2.7 86978 40883/47.0 51276/59.0 2726/3.1 3680 1685/45.8 2055/55.8 102/2.8

1000-1499 8213 3656/44.5 4284/52.2 173/2.1 28510 11301/39.6 14217/49.9 660/2.3 1378 594/43.1 715/51.9 32/2.3

1500-1999 6048 3426/56.7 3733/61.7 200/3.3 26161 13577/51.9 15368/58.7 1082/4.1 1138 553/48.6 629/55.3 43/3.8

2000-2499 1292 929/71.9 891/69.0 59/4.6 5566 3293/59.2 3443/61.9 218/3.9 250 140/56.0 142/56.8 18/7.2

2500-2999 400 344/86.0 310/77.5 28/7.0 1610 1115/69.3 1022/63.5 83/5.2 81 56/69.1 51/63.0 4/4.9

3000-3499 355 340/95.8 297/83.7 29/8.2 1259 1088/86.4 908/72.1 102/8.1 61 50/82.0 44/72.1 8/13.1

3500-3999 193 189/97.9 159/82.4 24/12.4 745 698/93.7 594/79.3 79/10.6

4000-4499 102 102/100.0 87/85.3 18/17.7 272 265/97.4 223/82.0 30/11.0

4500-4999 124 124/100.0 114/91.9 17/13.7

>5000 161 161/100.0 153/95.0 25/15.3

**Table S6. Prevalence of polycythemia in boys and girls age 6 to 59 month and in not pregnant and pregnant women older than 15 years;** **comparison of methods of evaluation**. The table shows the total number of individuals (N), the number of anemic individuals (n), and percent-polycythemia. cut-off s.l. is the threshold for polycythemia suggested by the WHO for sea level (boys, girls and pregnant women: 15.5 g/dl; non-pregnant women >15 years: 16.5 g/dl) ^2^, and 95% CI-up indicates the upper limit of the 95% confidence interval for residents living lower than 500 m above sea level. Δ altitude indicates the method for adjustment for altitude; WHO: altitude correction by the method suggested by the WHO ^2^; regression: slope of the linear regression. Age-groups according to the WHO ^2^ rather than the more detailed grouping shown in figure 2 because of low numbers in some subgroups. Values are only shown when the sample size was > 50 per group.

**6 - 59 mo (boys and girls) women > 15 y, not pregnant women > 15 y, pregnant**

cut-off, s.l.: **WHO WHO >95% CI-up WHO WHO >95% CI-up WHO WHO >95% CI-up**

Δ altitude: **WHO regression regression WHO regression regression WHO regression regression**

**N n/% n/% n/% N n/% n/% n/% N n/% n/% n/%**

**Polycythemia in Central America (CAm)** (no data on males >15y)

<500 10761 2/<0.1 2/<0.1 145/1.4 23523 41/0.2 20/0.1 275/1.2 1349 2/0.2 1/0.1 12/0.9

500-999 7261 7/0.1 4/<0.1 87/1.2 14511 66/0.5 26/0.2 236/1.6 862 3/0.4 0/0.0 14/1.6

1000-1499 5488 4/<0.1 3/<0.1 80/1.5 11902 48/0.4 8/0.1 128/1.1 713 1/0.1 1/0.1 6/0.8

1500-1999 3400 0/0.0 0/0.0 40/1.2 6623 22/0.3 7/0.1 77/1.2 405 1/0.3 0/0.0 4/1.0

2000-2499 1450 3/0.2 3/0.2 42/2.9 3161 17/0.5 11/0.4 61/1.9 170 1/0.6 1/0.6 3/1.8

2500-2999 650 0/0.0 0/0.0 10/1.5 1404 3/0.2 3/0.2 29/2.1 101 0/0.0 0/0.0 0/0.0

3000-3499 76 0/0.0 0/0.0 2/2.6 122 1/0.8 1/0.8 10/8.2

**Polycythemia in South America (SAm)** (no data on males >15y)

<500 7880 3/<0.1 3/<0.1 87/1.1 22816 22/0.1 12/<0.1 239/1.1 1061 0/0.0 0/0.0 15/1.4

500-999 1178 1/0.1 1/0.1 14/1.2 3574 4/0.1 1/<0.1 24/0.7 150 1/0.7 0/0.0 4/2.7

1000-1499 606 1/0.2 1/0.2 10/1.7 1692 9/0.5 1/<0.1 24/1.4 61 0/0.0 0/0.0 1/1.6

1500-1999 654 0/0.0 0/0.0 6/0.9 1717 4/0.2 0/0.0 19/1.1 67 0/0.0 0/0.0 0/0.0

2000-2499 1074 2/0.2 0/0.0 22/2.1 3072 10/0.3 1/<013 47/1.5 101 0/0.0 0/0.0 2/2.0

2500-2999 1621 0/0.0 0/0.0 21/1.3 3670 6/0.2 0/0.0 45/1.2 169 0/0.0 0/0.0 1/0.6

3000-3499 1934 0/0.0 0/0.0 37/1.9 5295 6/0.1 4/0.1 64/1.2 180 0/0.0 0/0.0 4/2.2

3500-3999 2632 0/0.0 2/0.1 71/2.7 5453 13/0.2 19/0.4 142/2.6 237 0/0.0 0/0.0 6/2.5

4000-4499 735 0/0.0 1/0.1 48/6.5 1778 8/0.5 26/1.5 145/8.2 64 0/0.0 0/0.0 2/3.1

4500-4999 98 0/0.0 1/1.0 3/3.1 238 2/0.8 12/5.0 27/11.3

**6 - 59 mo (boys and girls) women > 15 y, not pregnant women > 15 y, pregnant**

cut-off, s.l.: **WHO WHO >95% CI-up WHO WHO >95% CI-up WHO WHO >95% CI-up**

Δ altitude: **WHO regression regression WHO regression regression WHO regression regression**

**N n/% n/% n/% N n/% n/% n/% N n/% n/% n/%**

**Polycythemia in Central and Western Africa (CWAfr)**

<500 11585 5/<0.1 5/<0.1 176/1.5 12809 21/0.2 14/0.1 183/1.4 1428 2/0.1 2/0.1 24/1.7

500-999 9155 5/<0.1 5/<0.1 149/1.6 11633 26/0.2 15/0.1 140/1.2 1429 2/0.1 1/<0.1 11/0.8

1000-1499 5575 2/<0.1 0/0.0 76/1.4 3894 8/0.2 1/<0.1 59/1.5 442 3/0.7 0/0.0 12/2.7

1500-1999 2697 1/<0.1 1/<0.1 71/2.6 1836 11/0.7 3/0.2 35/1.9 221 1/0.5 0/0.0 4/1.8

2000-2499 131 0/0.0 0/0.0 6/4.6 163 2/1.2 0/0.0 4/2.5

2500-2999 56 0/0.0 0/0.0 0/0.0 70 0/0.0 0/0.0 0/0.0

**Polycythemia in Southern Africa (SAfr)**

<500 1291 2/0.2 0/0.0 22/1.7 2657 4/0.4 6/0.2 37/1.4 153 1/0.7 1/0.7 2/1.3

500-999 1467 1/<0.1 0/0.0 10/0.7 2757 11/0.4 4/0.2 39/1.4 140 3/2.1 2/1.4 3/2.4

1000-1499 2535 3/0.1 3/0.1 16/0.6 4763 27/0.6 11/0.2 68/1.4 274 3/1.1 2/0.7 4/1.5

1500-1999 2236 8/0.4 7/0.3 39/1.7 5448 40/0.7 13/0.2 62/1.4 230 0/0.0 0/0.0 1/0.4

2000-2499 956 2/0.2 2/0.2 10/1.1 1698 11/0.7 2/0.1 27/1.6 86 0/0.0 0/0.0 1/1.2

2500-2999 181 0/0.0 0/0.0 1/0.6 285 0/0.0 0/0.0 2/0.7

**Polycythemia in East Africa (EAfr)**

<500 19633 6/<0.1 4/<0.1 224/1.1 21290 21/0.1 9/<0.1 213/1.0 1961 1/<0.1 1/<0.1 25/1.3

500-999 12908 9/<0.1 4/<0.1 132/1.0 14717 52/0.4 7/<0.1 172/1.2 1588 7/0.4 1/<0.1 25/1.6

1000-1499 29946 26/0.1 19/<0.1 329/1.1 36160 104/0.3 13/<0.1 224/0.6 3371 14/0.4 3/0.1 28/0.8

1500-1999 17512 11/0.1 9/<0.1 175/1.0 22873 93/0.4 4/<0.1 121/0.5 1858 9/0.5 1/<0.1 19/1.0

2000-2499 6171 5/0.1 4/<0.1 87/1.4 10645 38/0.4 0/0.0 38/0.4 677 3/0.4 0/0.0 6/0.9

2500-2999 1303 0/0.0 0/0.0 13/1.0 2297 5/0.2 0/0.0 5/0.2 130 1/0.8 0/0.0 1/0.8

3000-3499 207 0/0.0 0/0.0 7/3.4 302 2/0.7 0/0.0 2/0.7

**Polycythemia in Middle East (ME)**

<500 2018 0/0.0 1/<0.1 69/3.4 1313 0/0.0 0/0.0 22/1.7 168 0/0.0 0/0.0 4/2.4

500-999 6456 1/<0.1 12/0.2 761/11.8 5967 7/0.1 5/0.1 115/1.9 763 0/0.0 0/0.0 21/2.8

1000-1499 2501 4</0.1 13/0.5 367/14.7 1601 1/<0.1 1/<0.1 25/1.6 190 0/0.0 0/0.0 3/1.6

1500-1999 933 1/<0.1 3/0.3 62/6.7 124 0/0.0 0/0.0 0/0.0

2000-2499 893 1/0.1 6/0.7 95/10.6

2500-2999 163 0/0.0 1/0.6 18/11.4

**6 - 59 mo (boys and girls) women > 15 y, not pregnant women > 15 y, pregnant**

cut-off, s.l.: **WHO WHO >95% CI-up WHO WHO >95% CI-up WHO WHO >95% CI-up**

Δ altitude: **WHO regression regression WHO regression regression WHO regression regression**

**N n/% n/% n/% N n/% n/% n/% N n/% n/% n/%**

**Polycythemia in West and Central Asian people (WCA)**

<500 2659 3/0.1 3/0.1 55/2.1 2644 2/0.1 1/<0.1 33/1.3 203 0/0.0 0/0.0 4/2.0

500-999 4646 4/0.1 3/<0.1 140/3.0 11448 14/0.1 7/<0.1 469/4.1 683 3/0.4 2/0.0 28/4.1

1000-1499 2528 3/0.1 3/0.1 80/3.2 7135 9/0.1 5/<0.1 273/3.8 374 0/0.0 0/0.0 9/2.4

1500-1999 1707 1/<0.1 1/<0.1 73/4.2 4935 4/0.1 3/<0.1 162/3.3 221 0/0.0 0/0.0 6/2.7

2000-2499 600 1/0.2 1/0.2 28/4.7 1867 1/<0.1 1/<0.1 59/3.2 65 0/0.0 0/0.0 3/4.6

2500-2999 144 0/0.0 0/0.0 3/2.1 283 0/0.0 0/0.0 6/2.1

3000-3499 113 0/0.0 0/0.0 1/0.90

3500-3999 73 1/1.4 1/1.4 5/6.9

**Polycythemia in South-South/East Asian people (SSEA)**

<500 133236 84/<0.1 72/<0.1 2015/1.5 508660 356/<0.1 291/<0.1 8329/1.6 24207 36/0.2 27/0.1 403/1.7

500-999 21936 9/<0.1 7/<0.1 436/2.0 86978 83/0.1 47/<0.1 1591/1.8 3680 8/0.2 5/0.1 84/2.3

1000-1499 8213 9/0.1 8/0.1 306/3.7 28510 35/0.1 22/0.1 1077/3.8 1378 2/0.2 1/<0.1 32/2.3

1500-1999 6048 9/0.2 9/0.2 202/3.3 26161 32/0.1 18/<0.1 727/2.8 1138 1/0.1 0/0.0 26/2.3

2000-2499 1292 2/0.2 2/0.2 35/2.7 5566 5/0.1 5/0.1 205/3.7 250 0/0.0 0/0.0 9/3.6

2500-2999 400 1/0.3 1/0.3 6/1.5 1610 4/0.3 5/0.3 57/3.5 81 0/0.0 0/0.0 3/3.7

3000-3499 355 0/0.0 0/0.0 2/0.6 1259 1/0.1 3/0.2 34/2.7 61 0/0.0 0/0.0 1/1.6

3500-3499 193 0/0.0 0/0.0 2/1.0 745 1/0.1 2/0.3 13/1.7

4000-4599 102 0/0.0 0/0.0 0/0.0 272 0/0.0 0/0.0 5/1.8

4500-4999 124 0/0.0 0/0.0 0/0.0

>5000 161 0/0.0 0/0.0 1/0.6

**Table S7 Results from analysis of variance to determine the difference in mean [Hb] between the altitude-ranges shown in the look-up tables S4 for each world region and age- and pregnancy subgroups.** Altitude ranges: 1 .. 0-499 m, 2 .. 500-999 m, 3 .. 1000- 1499 m, 4 .. 1500-1999 m, 5 .. 2000-2499 m, 6 .. 2500-2999 m, 7 .. 3000-3499 m, 8 .. 3500-3999 m, 9 .. 4000-4499 m, 10 .. 4500-4999 m, 11 .. >4999 m. Contrast indicates the compared altitude-ranges. Difference is the difference between the mean [Hb] values (g/dl) of the indicated altitude ranges ([Hb] left altitude minus [Hb] right altitude range), P shows the level of significance from Bonferroni – multiple pairwise comparisons with a confidence interval of 95%. Lo (95%) and Up (95%) are the lower and upper limit of the 95 % confidence interval of the difference (g/dl), respectively. Number of individuals in table S4.

**Central America, children 6 to 24 months vs. altitude range**

Contrast Difference P Significant Lo (95%) Up (95%)

1 vs 7 -1.778 <0.0001 Yes -2.539 -1.017

1 vs 6 -1.126 <0.0001 Yes -1.406 -0.846

1 vs 5 -0.944 <0.0001 Yes -1.135 -0.752

1 vs 4 -0.544 <0.0001 Yes -0.677 -0.411

1 vs 3 -0.383 <0.0001 Yes -0.495 -0.271

1 vs 2 -0.179 <0.0001 Yes -0.282 -0.076

2 vs 7 -1.599 <0.0001 Yes -2.361 -0.837

2 vs 6 -0.947 <0.0001 Yes -1.230 -0.664

2 vs 5 -0.765 <0.0001 Yes -0.962 -0.568

2 vs 4 -0.365 <0.0001 Yes -0.505 -0.225

2 vs 3 -0.204 <0.0001 Yes -0.324 -0.083

3 vs 7 -1.395 <0.0001 Yes -2.159 -0.632

3 vs 6 -0.743 <0.0001 Yes -1.030 -0.457

3 vs 5 -0.561 <0.0001 Yes -0.763 -0.359

3 vs 4 -0.161 0.001 Yes -0.308 -0.014

4 vs 7 -1.234 <0.0001 Yes -2.001 -0.467

4 vs 6 -0.582 <0.0001 Yes -0.877 -0.287

4 vs 5 -0.399 <0.0001 Yes -0.614 -0.185

5 vs 7 -0.835 0.001 Yes -1.614 -0.055

5 vs 6 -0.182 0.089 No -0.509 0.144

6 vs 7 -0.652 0.014 No -1.457 0.153

**Central America, children 25 to 59 months vs. altitude range**

Contrast Difference P Significant Lo (95%) Up (95%)

1 vs 7 -1.698 <0.0001 Yes -2.196 -1.199

1 vs 6 -1.321 <0.0001 Yes -1.492 -1.150

1 vs 5 -1.235 <0.0001 Yes -1.354 -1.116

1 vs 4 -0.795 <0.0001 Yes -0.880 -0.711

1 vs 3 -0.522 <0.0001 Yes -0.593 -0.451

1 vs 2 -0.290 <0.0001 Yes -0.355 -0.225

2 vs 7 -1.408 <0.0001 Yes -1.907 -0.908

2 vs 6 -1.031 <0.0001 Yes -1.204 -0.857

2 vs 5 -0.945 <0.0001 Yes -1.067 -0.822

2 vs 4 -0.505 <0.0001 Yes -0.594 -0.416

2 vs 3 -0.232 <0.0001 Yes -0.309 -0.156

3 vs 7 -1.176 <0.0001 Yes -1.676 -0.675

3 vs 6 -0.799 <0.0001 Yes -0.974 -0.623

3 vs 5 -0.712 <0.0001 Yes -0.838 -0.586

3 vs 4 -0.273 <0.0001 Yes -0.367 -0.179

4 vs 7 -0.903 <0.0001 Yes -1.405 -0.400

4 vs 6 -0.526 <0.0001 Yes -0.707 -0.344

4 vs 5 -0.440 <0.0001 Yes -0.574 -0.306

5 vs 7 -0.463 0.006 No -0.972 0.046

5 vs 6 -0.086 0.191 No -0.286 0.114

6 vs 7 -0.377 0.029 No -0.901 0.147

**Central America, not-pregnant women age 15 to 20 years vs altitude range**

Contrast Difference P Significant Lo (95%) Up (95%)

1 vs 7 -2.868 <0.0001 Yes -3.613 -2.122

1 vs 6 -2.084 <0.0001 Yes -2.293 -1.874

1 vs 5 -1.552 <0.0001 Yes -1.700 -1.405

1 vs 4 -1.056 <0.0001 Yes -1.169 -0.943

1 vs 3 -0.721 <0.0001 Yes -0.812 -0.630

1 vs 2 -0.405 <0.0001 Yes -0.488 -0.321

2 vs 7 -2.463 <0.0001 Yes -3.209 -1.717

2 vs 6 -1.679 <0.0001 Yes -1.892 -1.466

2 vs 5 -1.148 <0.0001 Yes -1.301 -0.994

2 vs 4 -0.651 <0.0001 Yes -0.772 -0.531

2 vs 3 -0.316 <0.0001 Yes -0.416 -0.217

3 vs 7 -2.147 <0.0001 Yes -2.894 -1.399

3 vs 6 -1.363 <0.0001 Yes -1.579 -1.146

3 vs 5 -0.831 <0.0001 Yes -0.989 -0.674

3 vs 4 -0.335 <0.0001 Yes -0.461 -0.209

4 vs 7 -1.812 <0.0001 Yes -2.562 -1.061

4 vs 6 -1.028 <0.0001 Yes -1.254 -0.801

4 vs 5 -0.496 <0.0001 Yes -0.668 -0.325

5 vs 7 -1.315 <0.0001 Yes -2.072 -0.559

5 vs 6 -0.531 <0.0001 Yes -0.777 -0.286

6 vs 7 -0.784 0.002 Yes -1.555 -0.013

**Central America, not-pregnant women age > 20 years vs altitude range**

Contrast Difference P Significant Lo (95%) Up (95%)

1 vs 7 -2.685 <0.0001 Yes -3.111 -2.259

1 vs 6 -1.789 <0.0001 Yes -1.921 -1.657

1 vs 5 -1.387 <0.0001 Yes -1.478 -1.297

1 vs 4 -0.928 <0.0001 Yes -0.993 -0.862

1 vs 3 -0.636 <0.0001 Yes -0.689 -0.583

1 vs 2 -0.411 <0.0001 Yes -0.461 -0.361

2 vs 7 -2.274 <0.0001 Yes -2.701 -1.847

2 vs 6 -1.379 <0.0001 Yes -1.513 -1.244

2 vs 5 -0.977 <0.0001 Yes -1.070 -0.883

2 vs 4 -0.517 <0.0001 Yes -0.587 -0.447

2 vs 3 -0.225 <0.0001 Yes -0.283 -0.167

3 vs 7 -2.049 <0.0001 Yes -2.477 -1.622

3 vs 6 -1.153 <0.0001 Yes -1.289 -1.018

3 vs 5 -0.752 <0.0001 Yes -0.847 -0.656

3 vs 4 -0.292 <0.0001 Yes -0.364 -0.220

4 vs 7 -1.757 <0.0001 Yes -2.186 -1.328

4 vs 6 -0.861 <0.0001 Yes -1.002 -0.720

4 vs 5 -0.460 <0.0001 Yes -0.563 -0.357

5 vs 7 -1.298 <0.0001 Yes -1.731 -0.864

5 vs 6 -0.402 <0.0001 Yes -0.556 -0.248

6 vs 7 -0.896 <0.0001 Yes -1.340 -0.452

**Central America, pregnant women age 15 to 20 years vs altitude range**

Contrast Difference P Significant Lo (95%) Up (95%)

1 vs 7 -1.923 <0.0001 Yes -3.329 -0.516

1 vs 6 -1.756 0.000 Yes -3.162 -0.350

1 vs 5 -1.217 0.009 No -2.623 0.189

1 vs 4 -1.058 0.022 No -2.464 0.348

1 vs 3 -0.821 0.075 No -2.228 0.585

1 vs 2 -0.453 0.326 No -1.860 0.953

2 vs 7 -1.469 0.002 Yes -2.876 -0.063

2 vs 6 -1.303 0.005 No -2.709 0.104

2 vs 5 -0.764 0.098 No -2.170 0.643

2 vs 4 -0.605 0.190 No -2.011 0.802

2 vs 3 -0.368 0.426 No -1.774 1.038

3 vs 7 -1.101 0.017 No -2.508 0.305

3 vs 6 -0.935 0.043 No -2.341 0.472

3 vs 5 -0.396 0.392 No -1.802 1.011

3 vs 4 -0.237 0.608 No -1.643 1.170

4 vs 7 -0.864 0.061 No -2.271 0.542

4 vs 6 -0.698 0.131 No -2.104 0.709

4 vs 5 -0.159 0.731 No -1.565 1.247

5 vs 7 -0.706 0.127 No -2.112 0.701

5 vs 6 -0.539 0.243 No -1.945 0.867

6 vs 7 -0.167 0.718 No -1.573 1.240

|  |  |  |  |
| --- | --- | --- | --- |

**Central America, pregnant women age > 20 years vs altitude range**

Contrast Difference P Significant Lo (95%) Up (95%)

1 vs 7 -1.735 <0.0001 Yes -3.018 -0.452

1 vs 6 -1.809 <0.0001 Yes -3.092 -0.526

1 vs 5 -1.226 0.004 No -2.509 0.057

1 vs 4 -0.957 0.023 No -2.240 0.326

1 vs 3 -0.696 0.099 No -1.979 0.587

1 vs 2 -0.387 0.359 No -1.670 0.896

2 vs 7 -1.348 0.001 Yes -2.632 -0.065

2 vs 6 -1.422 0.001 Yes -2.705 -0.139

2 vs 5 -0.839 0.047 No -2.122 0.444

2 vs 4 -0.570 0.177 No -1.853 0.713

2 vs 3 -0.310 0.463 No -1.593 0.973

3 vs 6 -1.113 0.008 No -2.396 0.170

3 vs 7 -1.039 0.014 No -2.322 0.244

3 vs 5 -0.529 0.210 No -1.812 0.754

3 vs 4 -0.261 0.537 No -1.544 1.022

4 vs 6 -0.852 0.044 No -2.135 0.431

4 vs 7 -0.778 0.065 No -2.061 0.505

4 vs 5 -0.269 0.524 No -1.552 1.014

5 vs 6 -0.583 0.167 No -1.866 0.700

5 vs 7 -0.510 0.227 No -1.793 0.773

7 vs 6 -0.074 0.861 No -1.357 1.209

**South America. children 6 to 24 months vs. altitude range**

Contrast Difference P Significant Lo (95%) Up (95%)

1 vs 10 -3.292 <0.0001 Yes -4.490 -2.093

1 vs 9 -2.831 <0.0001 Yes -4.030 -1.633

1 vs 11 -2.708 <0.0001 Yes -3.906 -1.510

1 vs 8 -2.095 <0.0001 Yes -3.294 -0.897

1 vs 7 -1.905 <0.0001 Yes -3.104 -0.707

1 vs 6 -1.341 0.000 Yes -2.539 -0.142

1 vs 5 -1.158 0.001 No -2.356 0.041

1 vs 4 -0.742 0.040 No -1.940 0.456

1 vs 3 -0.472 0.192 No -1.670 0.727

1 vs 2 -0.151 0.675 No -1.350 1.047

2 vs 10 -3.140 <0.0001 Yes -4.339 -1.942

2 vs 9 -2.680 <0.0001 Yes -3.878 -1.481

2 vs 11 -2.557 <0.0001 Yes -3.755 -1.358

2 vs 8 -1.944 <0.0001 Yes -3.142 -0.745

2 vs 7 -1.754 <0.0001 Yes -2.952 -0.555

2 vs 6 -1.189 0.001 No -2.388 0.009

2 vs 5 -1.006 0.005 No -2.205 0.192

2 vs 4 -0.591 0.102 No -1.789 0.608

2 vs 3 -0.320 0.375 No -1.519 0.878

3 vs 10 -2.820 <0.0001 Yes -4.019 -1.622

3 vs 9 -2.360 <0.0001 Yes -3.558 -1.161

3 vs 11 -2.236 <0.0001 Yes -3.435 -1.038

3 vs 8 -1.624 <0.0001 Yes -2.822 -0.425

3 vs 7 -1.434 <0.0001 Yes -2.632 -0.235

3 vs 6 -0.869 0.016 No -2.068 0.329

3 vs 5 -0.686 0.057 No -1.885 0.512

3 vs 4 -0.270 0.454 No -1.469 0.928

4 vs 10 -2.550 <0.0001 Yes -3.748 -1.351

4 vs 9 -2.089 <0.0001 Yes -3.288 -0.891

4 vs 11 -1.966 <0.0001 Yes -3.164 -0.768

4 vs 8 -1.353 0.000 Yes -2.552 -0.155

4 vs 7 -1.163 0.001 No -2.362 0.035

4 vs 6 -0.599 0.097 No -1.797 0.600

4 vs 5 -0.416 0.250 No -1.614 0.783

5 vs 10 -2.134 <0.0001 Yes -3.333 -0.936

5 vs 9 -1.674 <0.0001 Yes -2.872 -0.475

5 vs 11 -1.550 <0.0001 Yes -2.749 -0.352

5 vs 8 -0.937 0.009 No -2.136 0.261

5 vs 7 -0.748 0.038 No -1.946 0.451

5 vs 6 -0.183 0.612 No -1.381 1.015

6 vs 10 -1.951 <0.0001 Yes -3.150 -0.753

6 vs 9 -1.491 <0.0001 Yes -2.689 -0.292

6 vs 11 -1.367 0.000 Yes -2.566 -0.169

6 vs 8 -0.754 0.037 No -1.953 0.444

6 vs 7 -0.565 0.118 No -1.763 0.634

7 vs 10 -1.387 0.000 Yes -2.585 -0.188

7 vs 9 -0.926 0.010 No -2.124 0.272

7 vs 11 -0.803 0.026 No -2.001 0.396

7 vs 8 -0.190 0.599 No -1.388 1.009

8 vs 10 -1.197 0.001 No -2.395 0.002

8 vs 9 -0.736 0.042 No -1.935 0.462

8 vs 11 -0.613 0.090 No -1.811 0.586

11 vs 10 -0.584 0.106 No -1.782 0.615

11 vs 9 -0.123 0.733 No -1.322 1.075

9 vs 10 -0.461 0.202 No -1.659 0.738

**South America. children 25 to 59 months vs. altitude range**

Contrast Difference P Significant Lo (95%) Up (95%)

1 vs 11 -4.635 <0.0001 Yes -6.264 -3.006

1 vs 10 -3.353 <0.0001 Yes -3.843 -2.863

1 vs 9 -2.990 <0.0001 Yes -3.175 -2.805

1 vs 8 -2.247 <0.0001 Yes -2.355 -2.139

1 vs 7 -1.921 <0.0001 Yes -2.041 -1.801

1 vs 6 -1.386 <0.0001 Yes -1.515 -1.257

1 vs 5 -1.139 <0.0001 Yes -1.292 -0.986

1 vs 4 -0.833 <0.0001 Yes -1.028 -0.637

1 vs 3 -0.453 <0.0001 Yes -0.650 -0.256

1 vs 2 -0.130 0.004 No -0.280 0.020

2 vs 11 -4.505 <0.0001 Yes -6.140 -2.871

2 vs 10 -3.223 <0.0001 Yes -3.730 -2.716

2 vs 9 -2.860 <0.0001 Yes -3.086 -2.634

2 vs 8 -2.117 <0.0001 Yes -2.285 -1.949

2 vs 7 -1.791 <0.0001 Yes -1.967 -1.615

2 vs 6 -1.256 <0.0001 Yes -1.438 -1.074

2 vs 5 -1.010 <0.0001 Yes -1.210 -0.809

2 vs 4 -0.703 <0.0001 Yes -0.937 -0.469

2 vs 3 -0.323 <0.0001 Yes -0.558 -0.088

3 vs 11 -4.182 <0.0001 Yes -5.821 -2.543

3 vs 10 -2.900 <0.0001 Yes -3.423 -2.377

3 vs 9 -2.537 <0.0001 Yes -2.797 -2.278

3 vs 8 -1.794 <0.0001 Yes -2.005 -1.583

3 vs 7 -1.468 <0.0001 Yes -1.685 -1.250

3 vs 6 -0.933 <0.0001 Yes -1.156 -0.711

3 vs 5 -0.686 <0.0001 Yes -0.924 -0.449

3 vs 4 -0.380 <0.0001 Yes -0.646 -0.113

4 vs 11 -3.802 <0.0001 Yes -5.442 -2.163

4 vs 10 -2.520 <0.0001 Yes -3.042 -1.998

4 vs 9 -2.157 <0.0001 Yes -2.416 -1.899

4 vs 8 -1.414 <0.0001 Yes -1.624 -1.204

4 vs 7 -1.088 <0.0001 Yes -1.304 -0.872

4 vs 6 -0.553 <0.0001 Yes -0.774 -0.332

4 vs 5 -0.307 <0.0001 Yes -0.543 -0.071

5 vs 11 -3.496 <0.0001 Yes -5.130 -1.861

5 vs 10 -2.214 <0.0001 Yes -2.722 -1.706

5 vs 9 -1.851 <0.0001 Yes -2.079 -1.623

5 vs 8 -1.107 <0.0001 Yes -1.278 -0.936

5 vs 7 -0.781 <0.0001 Yes -0.960 -0.602

5 vs 6 -0.247 <0.0001 Yes -0.432 -0.062

6 vs 11 -3.249 <0.0001 Yes -4.882 -1.617

6 vs 10 -1.967 <0.0001 Yes -2.468 -1.466

6 vs 9 -1.604 <0.0001 Yes -1.816 -1.392

6 vs 8 -0.861 <0.0001 Yes -1.010 -0.711

6 vs 7 -0.535 <0.0001 Yes -0.694 -0.376

7 vs 11 -2.714 <0.0001 Yes -4.346 -1.083

7 vs 10 -1.432 <0.0001 Yes -1.931 -0.933

7 vs 9 -1.069 <0.0001 Yes -1.277 -0.862

7 vs 8 -0.326 <0.0001 Yes -0.468 -0.184

8 vs 11 -2.388 <0.0001 Yes -4.019 -0.757

8 vs 10 -1.106 <0.0001 Yes -1.602 -0.610

8 vs 9 -0.743 <0.0001 Yes -0.944 -0.543

9 vs 11 -1.645 0.001 Yes -3.283 -0.007

9 vs 10 -0.363 0.020 No -0.882 0.156

10 vs 11 -1.282 0.012 No -2.982 0.418

**South America. not-pregnant women age 15 to 20 years vs altitude range**

Contrast Difference P Significant Lo (95%) Up (95%)

1 vs 10 -4.357 <0.0001 Yes -5.456 -3.258

1 vs 11 -4.060 <0.0001 Yes -5.159 -2.961

1 vs 9 -3.564 <0.0001 Yes -4.663 -2.465

1 vs 8 -2.828 <0.0001 Yes -3.927 -1.729

1 vs 7 -2.477 <0.0001 Yes -3.576 -1.378

1 vs 6 -2.043 <0.0001 Yes -3.142 -0.944

1 vs 5 -1.593 <0.0001 Yes -2.692 -0.494

1 vs 4 -1.027 0.002 No -2.126 0.072

1 vs 3 -0.697 0.035 No -1.796 0.402

1 vs 2 -0.206 0.533 No -1.305 0.893

2 vs 10 -4.150 <0.0001 Yes -5.249 -3.051

2 vs 11 -3.854 <0.0001 Yes -4.953 -2.755

2 vs 9 -3.358 <0.0001 Yes -4.457 -2.259

2 vs 8 -2.621 <0.0001 Yes -3.720 -1.522

2 vs 7 -2.270 <0.0001 Yes -3.369 -1.171

2 vs 6 -1.836 <0.0001 Yes -2.935 -0.737

2 vs 5 -1.387 <0.0001 Yes -2.486 -0.288

2 vs 4 -0.820 0.013 No -1.919 0.279

2 vs 3 -0.490 0.139 No -1.589 0.609

3 vs 10 -3.660 <0.0001 Yes -4.759 -2.561

3 vs 11 -3.364 <0.0001 Yes -4.463 -2.265

3 vs 9 -2.867 <0.0001 Yes -3.966 -1.768

3 vs 8 -2.131 <0.0001 Yes -3.230 -1.032

3 vs 7 -1.780 <0.0001 Yes -2.879 -0.681

3 vs 6 -1.346 <0.0001 Yes -2.445 -0.247

3 vs 5 -0.896 0.007 No -1.995 0.203

3 vs 4 -0.330 0.319 No -1.429 0.769

4 vs 10 -3.330 <0.0001 Yes -4.429 -2.231

4 vs 11 -3.034 <0.0001 Yes -4.133 -1.935

4 vs 9 -2.537 <0.0001 Yes -3.636 -1.438

4 vs 8 -1.801 <0.0001 Yes -2.900 -0.702

4 vs 7 -1.450 <0.0001 Yes -2.549 -0.351

4 vs 6 -1.016 0.002 No -2.115 0.083

4 vs 5 -0.566 0.087 No -1.665 0.533

5 vs 10 -2.764 <0.0001 Yes -3.863 -1.665

5 vs 11 -2.467 <0.0001 Yes -3.566 -1.368

5 vs 9 -1.971 <0.0001 Yes -3.070 -0.872

5 vs 8 -1.235 0.000 Yes -2.334 -0.136

5 vs 7 -0.884 0.008 No -1.983 0.215

5 vs 6 -0.450 0.174 No -1.549 0.649

6 vs 10 -2.314 <0.0001 Yes -3.413 -1.215

6 vs 11 -2.018 <0.0001 Yes -3.117 -0.919

6 vs 9 -1.521 <0.0001 Yes -2.620 -0.422

6 vs 8 -0.785 0.018 No -1.884 0.314

6 vs 7 -0.434 0.190 No -1.533 0.665

7 vs 10 -1.880 <0.0001 Yes -2.979 -0.781

7 vs 11 -1.583 <0.0001 Yes -2.682 -0.484

7 vs 9 -1.087 0.001 No -2.186 0.012

7 vs 8 -0.351 0.290 No -1.450 0.748

8 vs 10 -1.529 <0.0001 Yes -2.628 -0.430

8 vs 11 -1.233 0.000 Yes -2.332 -0.134

8 vs 9 -0.737 0.026 No -1.836 0.362

9 vs 10 -0.793 0.017 No -1.892 0.306

9 vs 11 -0.496 0.134 No -1.595 0.603

11 vs 10 -0.296 0.371 No -1.395 0.803

**South America. not-pregnant women age > 20 years vs altitude range**

Contrast Difference P Significant Lo (95%) Up (95%)

1 vs 11 -4.943 <0.0001 Yes -5.868 -4.018

1 vs 10 -4.223 <0.0001 Yes -4.537 -3.909

1 vs 9 -3.493 <0.0001 Yes -3.617 -3.369

1 vs 8 -2.637 <0.0001 Yes -2.714 -2.560

1 vs 7 -2.357 <0.0001 Yes -2.435 -2.280

1 vs 6 -1.908 <0.0001 Yes -1.998 -1.817

1 vs 5 -1.505 <0.0001 Yes -1.602 -1.408

1 vs 4 -0.891 <0.0001 Yes -1.018 -0.765

1 vs 3 -0.595 <0.0001 Yes -0.721 -0.469

1 vs 2 -0.159 <0.0001 Yes -0.249 -0.069

2 vs 11 -4.784 <0.0001 Yes -5.712 -3.856

2 vs 10 -4.064 <0.0001 Yes -4.387 -3.740

2 vs 9 -3.334 <0.0001 Yes -3.480 -3.188

2 vs 8 -2.478 <0.0001 Yes -2.586 -2.369

2 vs 7 -2.198 <0.0001 Yes -2.307 -2.089

2 vs 6 -1.749 <0.0001 Yes -1.867 -1.630

2 vs 5 -1.346 <0.0001 Yes -1.469 -1.222

2 vs 4 -0.732 <0.0001 Yes -0.880 -0.584

2 vs 3 -0.436 <0.0001 Yes -0.584 -0.288

3 vs 11 -4.348 <0.0001 Yes -5.280 -3.416

3 vs 10 -3.628 <0.0001 Yes -3.963 -3.292

3 vs 9 -2.898 <0.0001 Yes -3.069 -2.728

3 vs 8 -2.042 <0.0001 Yes -2.182 -1.902

3 vs 7 -1.762 <0.0001 Yes -1.903 -1.622

3 vs 6 -1.313 <0.0001 Yes -1.461 -1.165

3 vs 5 -0.910 <0.0001 Yes -1.062 -0.758

3 vs 4 -0.296 <0.0001 Yes -0.469 -0.124

4 vs 11 -4.052 <0.0001 Yes -4.984 -3.120

4 vs 10 -3.331 <0.0001 Yes -3.667 -2.996

4 vs 9 -2.602 <0.0001 Yes -2.773 -2.431

4 vs 8 -1.745 <0.0001 Yes -1.886 -1.605

4 vs 7 -1.466 <0.0001 Yes -1.607 -1.325

4 vs 6 -1.017 <0.0001 Yes -1.165 -0.868

4 vs 5 -0.613 <0.0001 Yes -0.766 -0.461

5 vs 11 -3.438 <0.0001 Yes -4.367 -2.510

5 vs 10 -2.718 <0.0001 Yes -3.043 -2.393

5 vs 9 -1.989 <0.0001 Yes -2.139 -1.838

5 vs 8 -1.132 <0.0001 Yes -1.246 -1.018

5 vs 7 -0.853 <0.0001 Yes -0.967 -0.738

5 vs 6 -0.403 <0.0001 Yes -0.527 -0.279

6 vs 11 -3.035 <0.0001 Yes -3.963 -2.107

6 vs 10 -2.315 <0.0001 Yes -2.639 -1.991

6 vs 9 -1.585 <0.0001 Yes -1.731 -1.439

6 vs 8 -0.729 <0.0001 Yes -0.838 -0.620

6 vs 7 -0.450 <0.0001 Yes -0.559 -0.340

7 vs 11 -2.586 <0.0001 Yes -3.513 -1.659

7 vs 10 -1.865 <0.0001 Yes -2.186 -1.545

7 vs 9 -1.136 <0.0001 Yes -1.274 -0.998

7 vs 8 -0.279 <0.0001 Yes -0.378 -0.181

8 vs 11 -2.306 <0.0001 Yes -3.233 -1.379

8 vs 10 -1.586 <0.0001 Yes -1.906 -1.266

8 vs 9 -0.857 <0.0001 Yes -0.994 -0.719

9 vs 11 -1.450 <0.0001 Yes -2.382 -0.518

9 vs 10 -0.729 <0.0001 Yes -1.064 -0.395

10 vs 11 -0.720 0.014 No -1.696 0.255

**South America. pregnant women age 15 to 20 years vs altitude range**

Contrast Difference P Significant Lo (95%) Up (95%)

1 vs 9 -3.903 <0.0001 Yes -6.346 -1.460

1 vs 10 -3.467 <0.0001 Yes -5.910 -1.024

1 vs 8 -2.962 <0.0001 Yes -5.405 -0.519

1 vs 7 -2.432 0.001 No -4.875 0.011

1 vs 6 -2.142 0.004 No -4.585 0.301

1 vs 5 -1.024 0.169 No -3.467 1.419

1 vs 4 -0.692 0.353 No -3.135 1.751

1 vs 3 -0.667 0.370 No -3.110 1.776

1 vs 2 -0.439 0.555 No -2.882 2.004

2 vs 9 -3.465 <0.0001 Yes -5.908 -1.022

2 vs 10 -3.028 <0.0001 Yes -5.471 -0.585

2 vs 8 -2.524 0.001 Yes -4.967 -0.081

2 vs 7 -1.994 0.008 No -4.437 0.449

2 vs 6 -1.703 0.022 No -4.146 0.740

2 vs 5 -0.585 0.431 No -3.028 1.858

2 vs 4 -0.253 0.734 No -2.696 2.190

2 vs 3 -0.228 0.759 No -2.671 2.215

3 vs 9 -3.236 <0.0001 Yes -5.679 -0.793

3 vs 10 -2.800 0.000 Yes -5.243 -0.357

3 vs 8 -2.295 0.002 No -4.738 0.148

3 vs 7 -1.765 0.018 No -4.208 0.678

3 vs 6 -1.475 0.048 No -3.918 0.968

3 vs 5 -0.357 0.631 No -2.800 2.086

3 vs 4 -0.025 0.973 No -2.468 2.418

4 vs 9 -3.211 <0.0001 Yes -5.654 -0.768

4 vs 10 -2.775 0.000 Yes -5.218 -0.332

4 vs 8 -2.270 0.002 No -4.713 0.173

4 vs 7 -1.740 0.020 No -4.183 0.703

4 vs 6 -1.450 0.052 No -3.893 0.993

4 vs 5 -0.332 0.655 No -2.775 2.111

5 vs 9 -2.879 0.000 Yes -5.322 -0.436

5 vs 10 -2.443 0.001 No -4.886 0.000

5 vs 8 -1.938 0.009 No -4.381 0.505

5 vs 7 -1.408 0.059 No -3.851 1.035

5 vs 6 -1.118 0.133 No -3.561 1.325

6 vs 9 -1.761 0.018 No -4.204 0.682

6 vs 10 -1.325 0.075 No -3.768 1.118

6 vs 8 -0.820 0.270 No -3.263 1.623

6 vs 7 -0.290 0.696 No -2.733 2.153

7 vs 9 -1.471 0.049 No -3.914 0.972

7 vs 10 -1.035 0.165 No -3.478 1.408

7 vs 8 -0.530 0.476 No -2.973 1.913

8 vs 9 -0.941 0.206 No -3.384 1.502

8 vs 10 -0.505 0.498 No -2.948 1.938

10 vs 9 -0.436 0.557 No -2.879 2.007

**South America. pregnant women age > 20 years vs altitude range**

Contrast Difference P Significant Lo (95%) Up (95%)

1 vs 11 -4.350 <0.0001 Yes -6.536 -2.165

1 vs 10 -3.413 <0.0001 Yes -5.598 -1.228

1 vs 9 -3.111 <0.0001 Yes -5.296 -0.925

1 vs 8 -2.411 0.000 Yes -4.596 -0.225

1 vs 7 -2.209 0.001 Yes -4.395 -0.024

1 vs 6 -1.774 0.007 No -3.959 0.412

1 vs 5 -1.362 0.039 No -3.547 0.823

1 vs 4 -0.946 0.150 No -3.131 1.239

1 vs 3 -0.627 0.340 No -2.812 1.558

1 vs 2 -0.143 0.828 No -2.328 2.042

2 vs 11 -4.207 <0.0001 Yes -6.392 -2.022

2 vs 10 -3.270 <0.0001 Yes -5.455 -1.084

2 vs 9 -2.968 <0.0001 Yes -5.153 -0.782

2 vs 8 -2.268 0.001 Yes -4.453 -0.082

2 vs 7 -2.066 0.002 No -4.252 0.119

2 vs 6 -1.631 0.013 No -3.816 0.555

2 vs 5 -1.219 0.064 No -3.404 0.967

2 vs 4 -0.803 0.222 No -2.988 1.382

2 vs 3 -0.484 0.462 No -2.669 1.701

3 vs 11 -3.723 <0.0001 Yes -5.908 -1.538

3 vs 10 -2.786 <0.0001 Yes -4.971 -0.600

3 vs 9 -2.483 0.000 Yes -4.669 -0.298

3 vs 8 -1.783 0.007 No -3.969 0.402

3 vs 7 -1.582 0.016 No -3.767 0.603

3 vs 6 -1.146 0.081 No -3.332 1.039

3 vs 5 -0.735 0.264 No -2.920 1.451

3 vs 4 -0.319 0.628 No -2.504 1.866

4 vs 11 -3.404 <0.0001 Yes -5.590 -1.219

4 vs 10 -2.467 0.000 Yes -4.652 -0.281

4 vs 9 -2.165 0.001 No -4.350 0.021

4 vs 8 -1.465 0.026 No -3.650 0.721

4 vs 7 -1.263 0.055 No -3.449 0.922

4 vs 6 -0.828 0.208 No -3.013 1.358

4 vs 5 -0.416 0.527 No -2.601 1.770

5 vs 11 -2.989 <0.0001 Yes -5.174 -0.803

5 vs 10 -2.051 0.002 No -4.236 0.134

5 vs 9 -1.749 0.008 No -3.934 0.436

5 vs 8 -1.049 0.111 No -3.234 1.136

5 vs 7 -0.848 0.198 No -3.033 1.338

5 vs 6 -0.412 0.531 No -2.597 1.773

6 vs 11 -2.577 <0.0001 Yes -4.762 -0.391

6 vs 10 -1.639 0.013 No -3.824 0.546

6 vs 9 -1.337 0.042 No -3.522 0.848

6 vs 8 -0.637 0.333 No -2.822 1.548

6 vs 7 -0.436 0.508 No -2.621 1.750

7 vs 11 -2.141 0.001 No -4.326 0.044

7 vs 10 -1.203 0.067 No -3.389 0.982

7 vs 9 -0.901 0.171 No -3.087 1.284

7 vs 8 -0.201 0.760 No -2.386 1.984

8 vs 11 -1.940 0.003 No -4.125 0.246

8 vs 10 -1.002 0.128 No -3.187 1.183

8 vs 9 -0.700 0.287 No -2.885 1.485

9 vs 11 -1.240 0.060 No -3.425 0.946

9 vs 10 -0.302 0.646 No -2.487 1.883

10 vs 11 -0.938 0.154 No -3.123 1.248

**West and Central Africa. children 6 to 24 months vs. altitude range**

Contrast Difference P Significant Lo (95%) Up (95%)

6 vs 5 -1.365 0.029 No -3.315 0.585

6 vs 4 -1.232 0.048 No -3.182 0.717

6 vs 8 -1.058 0.090 No -3.008 0.891

6 vs 3 -0.707 0.257 No -2.656 1.243

6 vs 7 -0.425 0.496 No -2.375 1.525

6 vs 2 -0.272 0.663 No -2.222 1.678

6 vs 1 -0.055 0.930 No -2.004 1.895

1 vs 5 -1.310 0.036 No -3.260 0.639

1 vs 4 -1.178 0.059 No -3.127 0.772

1 vs 8 -1.004 0.108 No -2.953 0.946

1 vs 3 -0.652 0.296 No -2.602 1.298

1 vs 7 -0.370 0.553 No -2.320 1.579

1 vs 2 -0.217 0.728 No -2.167 1.732

2 vs 5 -1.093 0.080 No -3.043 0.857

2 vs 4 -0.960 0.124 No -2.910 0.989

2 vs 8 -0.786 0.208 No -2.736 1.163

2 vs 3 -0.435 0.486 No -2.384 1.515

2 vs 7 -0.153 0.806 No -2.103 1.797

7 vs 5 -0.940 0.132 No -2.890 1.010

7 vs 4 -0.807 0.196 No -2.757 1.142

7 vs 8 -0.633 0.310 No -2.583 1.316

7 vs 3 -0.282 0.652 No -2.231 1.668

3 vs 5 -0.658 0.291 No -2.608 1.291

3 vs 4 -0.526 0.399 No -2.475 1.424

3 vs 8 -0.352 0.573 No -2.301 1.598

8 vs 5 -0.307 0.623 No -2.256 1.643

8 vs 4 -0.174 0.780 No -2.124 1.776

4 vs 5 -0.133 0.832 No -2.082 1.817

**West and Central Africa. children 25 to 59 months vs. altitude range**

Contrast Difference P Significant Lo (95%) Up (95%)

6 vs 5 -1.756 <0.0001 Yes -2.946 -0.567

6 vs 4 -1.608 <0.0001 Yes -2.798 -0.419

6 vs 3 -1.091 0.004 No -2.280 0.099

6 vs 8 -0.631 0.098 No -1.820 0.559

6 vs 2 -0.490 0.199 No -1.679 0.700

6 vs 1 -0.369 0.332 No -1.559 0.820

6 vs 7 -0.176 0.643 No -1.366 1.013

7 vs 5 -1.580 <0.0001 Yes -2.769 -0.390

7 vs 4 -1.432 0.000 Yes -2.622 -0.242

7 vs 3 -0.914 0.016 No -2.104 0.275

7 vs 8 -0.454 0.233 No -1.644 0.735

7 vs 2 -0.313 0.411 No -1.503 0.876

7 vs 1 -0.193 0.612 No -1.382 0.997

1 vs 5 -1.387 0.000 Yes -2.577 -0.197

1 vs 4 -1.239 0.001 Yes -2.429 -0.049

1 vs 3 -0.722 0.058 No -1.911 0.468

1 vs 8 -0.261 0.492 No -1.451 0.928

1 vs 2 -0.120 0.752 No -1.310 1.069

2 vs 5 -1.267 0.001 Yes -2.456 -0.077

2 vs 4 -1.119 0.003 No -2.308 0.071

2 vs 3 -0.601 0.114 No -1.791 0.588

2 vs 8 -0.141 0.711 No -1.331 1.049

8 vs 5 -1.126 0.003 No -2.315 0.064

8 vs 4 -0.978 0.010 No -2.167 0.212

8 vs 3 -0.460 0.227 No -1.650 0.729

3 vs 5 -0.665 0.081 No -1.855 0.524

3 vs 4 -0.517 0.174 No -1.707 0.672

4 vs 5 -0.148 0.698 No -1.338 1.042

**West and Central Africa. not-pregnant women age 15 to 20 years vs altitude range**

Contrast Difference P Significant Lo (95%) Up (95%)

7 vs 5 -3.308 0.001 Yes -6.370 -0.246

7 vs 4 -2.936 0.003 No -5.998 0.127

7 vs 3 -2.586 0.008 No -5.648 0.476

7 vs 8 -2.400 0.014 No -5.462 0.662

7 vs 2 -1.890 0.054 No -4.952 1.172

7 vs 1 -1.618 0.099 No -4.680 1.444

7 vs 6 -1.143 0.244 No -4.205 1.919

6 vs 5 -2.165 0.027 No -5.227 0.897

6 vs 4 -1.793 0.067 No -4.855 1.269

6 vs 3 -1.443 0.141 No -4.505 1.619

6 vs 8 -1.257 0.200 No -4.319 1.805

6 vs 2 -0.747 0.446 No -3.809 2.315

6 vs 1 -0.475 0.628 No -3.538 2.587

1 vs 5 -1.690 0.085 No -4.752 1.373

1 vs 4 -1.318 0.179 No -4.380 1.745

1 vs 3 -0.968 0.323 No -4.030 2.094

1 vs 8 -0.782 0.425 No -3.844 2.280

1 vs 2 -0.272 0.782 No -3.334 2.791

2 vs 5 -1.418 0.148 No -4.480 1.644

2 vs 4 -1.046 0.286 No -4.108 2.016

2 vs 3 -0.696 0.477 No -3.758 2.366

2 vs 8 -0.510 0.603 No -3.572 2.552

8 vs 5 -0.908 0.354 No -3.970 2.154

8 vs 4 -0.536 0.585 No -3.598 2.527

8 vs 3 -0.186 0.849 No -3.248 2.876

3 vs 5 -0.722 0.461 No -3.784 2.340

3 vs 4 -0.350 0.721 No -3.412 2.712

4 vs 5 -0.372 0.704 No -3.434 2.690

**West and Central Africa. not-pregnant women age > 20 years vs altitude range**

Contrast Difference P Significant Lo (95%) Up (95%)

7 vs 5 -1.463 0.002 No -2.927 0.000

7 vs 4 -1.412 0.003 No -2.875 0.052

7 vs 8 -1.079 0.021 No -2.542 0.385

7 vs 3 -0.920 0.050 No -2.383 0.544

7 vs 6 -0.533 0.256 No -1.996 0.931

7 vs 2 -0.393 0.402 No -1.856 1.071

7 vs 1 -0.183 0.696 No -1.646 1.281

1 vs 5 -1.280 0.006 No -2.744 0.183

1 vs 4 -1.229 0.009 No -2.693 0.234

1 vs 8 -0.896 0.056 No -2.359 0.568

1 vs 3 -0.737 0.116 No -2.201 0.726

1 vs 6 -0.350 0.455 No -1.813 1.114

1 vs 2 -0.210 0.654 No -1.674 1.253

2 vs 5 -1.070 0.022 No -2.534 0.393

2 vs 4 -1.019 0.030 No -2.483 0.444

2 vs 8 -0.686 0.143 No -2.149 0.778

2 vs 3 -0.527 0.261 No -1.991 0.936

2 vs 6 -0.140 0.765 No -1.603 1.324

6 vs 5 -0.931 0.047 No -2.394 0.533

6 vs 4 -0.879 0.061 No -2.343 0.584

6 vs 8 -0.546 0.244 No -2.009 0.918

6 vs 3 -0.387 0.408 No -1.851 1.076

3 vs 5 -0.543 0.246 No -2.007 0.920

3 vs 4 -0.492 0.294 No -1.955 0.972

3 vs 8 -0.159 0.735 No -1.622 1.305

8 vs 5 -0.385 0.412 No -1.848 1.079

8 vs 4 -0.333 0.477 No -1.797 1.130

4 vs 5 -0.051 0.913 No -1.515 1.412

**West and Central Africa. pregnant women age 15 to 20 years vs altitude range**

Contrast Difference P Significant Lo (95%) Up (95%)

6 vs 4 -3.446 0.003 Yes -6.867 -0.026

6 vs 3 -2.143 0.065 No -5.563 1.278

6 vs 2 -1.673 0.150 No -5.093 1.748

6 vs 1 -1.620 0.163 No -5.040 1.801

6 vs 5 -0.733 0.528 No -4.154 2.687

5 vs 4 -2.713 0.020 No -6.133 0.707

5 vs 3 -1.410 0.225 No -4.830 2.011

5 vs 2 -0.940 0.419 No -4.360 2.481

5 vs 1 -0.886 0.445 No -4.307 2.534

1 vs 4 -1.827 0.116 No -5.247 1.594

1 vs 3 -0.523 0.652 No -3.944 2.897

1 vs 2 -0.053 0.963 No -3.474 3.367

2 vs 4 -1.774 0.127 No -5.194 1.647

2 vs 3 -0.470 0.686 No -3.890 2.950

3 vs 4 -1.304 0.262 No -4.724 2.117

**West and Central Africa. pregnant women age > 20 years vs altitude range**

Contrast Difference P Significant Lo (95%) Up (95%)

7 vs 8 -4.333 <0.0001 Yes -7.072 -1.595

7 vs 5 -3.489 <0.0001 Yes -6.228 -0.750

7 vs 4 -3.414 <0.0001 Yes -6.153 -0.675

7 vs 6 -2.840 0.001 Yes -5.579 -0.101

7 vs 3 -2.817 0.001 Yes -5.556 -0.078

7 vs 2 -2.209 0.012 No -4.948 0.530

7 vs 1 -2.004 0.022 No -4.743 0.735

1 vs 8 -2.329 0.008 No -5.068 0.410

1 vs 5 -1.485 0.090 No -4.223 1.254

1 vs 4 -1.410 0.108 No -4.149 1.329

1 vs 6 -0.836 0.340 No -3.575 1.903

1 vs 3 -0.813 0.354 No -3.551 1.926

1 vs 2 -0.205 0.815 No -2.944 2.534

2 vs 8 -2.124 0.015 No -4.863 0.615

2 vs 5 -1.280 0.144 No -4.019 1.459

2 vs 4 -1.205 0.169 No -3.944 1.534

2 vs 6 -0.631 0.471 No -3.370 2.108

2 vs 3 -0.608 0.488 No -3.346 2.131

3 vs 8 -1.516 0.084 No -4.255 1.222

3 vs 5 -0.672 0.443 No -3.411 2.067

3 vs 4 -0.597 0.495 No -3.336 2.142

3 vs 6 -0.023 0.979 No -2.762 2.716

6 vs 8 -1.493 0.088 No -4.232 1.245

6 vs 5 -0.649 0.459 No -3.388 2.090

6 vs 4 -0.574 0.512 No -3.313 2.165

4 vs 8 -0.919 0.294 No -3.658 1.820

4 vs 5 -0.075 0.932 No -2.814 2.664

5 vs 8 -0.844 0.335 No -3.583 1.894

**Southern Africa. children 6 to 24 months vs. altitude range**

Contrast Difference P Significant Lo (95%) Up (95%)

1 vs 6 -1.341 <0.0001 Yes -2.035 -0.647

1 vs 5 -1.046 <0.0001 Yes -1.390 -0.702

1 vs 4 -0.851 <0.0001 Yes -1.123 -0.579

1 vs 3 -0.423 <0.0001 Yes -0.681 -0.165

1 vs 2 -0.200 0.047 No -0.497 0.096

2 vs 6 -1.141 <0.0001 Yes -1.836 -0.446

2 vs 5 -0.845 <0.0001 Yes -1.191 -0.499

2 vs 4 -0.650 <0.0001 Yes -0.925 -0.376

2 vs 3 -0.223 0.012 No -0.483 0.038

3 vs 6 -0.918 <0.0001 Yes -1.597 -0.239

3 vs 5 -0.623 <0.0001 Yes -0.936 -0.309

3 vs 4 -0.428 <0.0001 Yes -0.659 -0.196

4 vs 6 -0.490 0.036 No -1.175 0.195

4 vs 5 -0.195 0.078 No -0.520 0.130

5 vs 6 -0.295 0.226 No -1.012 0.421

**Southern Africa. children 25 to 59 months vs. altitude range**

Contrast Difference P Significant Lo (95%) Up (95%)

1 vs 6 -0.884 <0.0001 Yes -1.269 -0.499

1 vs 5 -0.639 <0.0001 Yes -0.854 -0.424

1 vs 4 -0.327 <0.0001 Yes -0.505 -0.148

1 vs 3 -0.086 0.152 No -0.263 0.090

1 vs 2 -0.026 0.694 No -0.220 0.168

2 vs 6 -0.858 <0.0001 Yes -1.238 -0.478

2 vs 5 -0.613 <0.0001 Yes -0.818 -0.408

2 vs 4 -0.301 <0.0001 Yes -0.467 -0.134

2 vs 3 -0.060 0.285 No -0.225 0.105

3 vs 6 -0.798 <0.0001 Yes -1.169 -0.427

3 vs 5 -0.553 <0.0001 Yes -0.742 -0.364

3 vs 4 -0.241 <0.0001 Yes -0.386 -0.095

4 vs 6 -0.558 <0.0001 Yes -0.930 -0.186

4 vs 5 -0.312 <0.0001 Yes -0.503 -0.122

5 vs 6 -0.245 0.066 No -0.636 0.146

**Southern Africa. not-pregnant women age 15 to 20 years vs altitude range**

Contrast Difference P Significant Lo (95%) Up (95%)

1 vs 6 -1.694 <0.0001 Yes -2.354 -1.034

1 vs 5 -1.393 <0.0001 Yes -1.692 -1.094

1 vs 4 -0.806 <0.0001 Yes -1.040 -0.572

1 vs 3 -0.550 <0.0001 Yes -0.792 -0.307

1 vs 2 -0.044 0.632 No -0.311 0.224

2 vs 6 -1.650 <0.0001 Yes -2.309 -0.992

2 vs 5 -1.349 <0.0001 Yes -1.645 -1.054

2 vs 4 -0.763 <0.0001 Yes -0.992 -0.533

2 vs 3 -0.506 <0.0001 Yes -0.744 -0.268

3 vs 6 -1.144 <0.0001 Yes -1.793 -0.496

3 vs 5 -0.843 <0.0001 Yes -1.116 -0.570

3 vs 4 -0.256 0.000 Yes -0.456 -0.056

4 vs 6 -0.888 <0.0001 Yes -1.533 -0.242

4 vs 5 -0.587 <0.0001 Yes -0.852 -0.321

5 vs 6 -0.301 0.188 No -0.973 0.371

**Southern Africa. not-pregnant women age > 20 years vs altitude range**

Contrast Difference P Significant Lo (95%) Up (95%)

1 vs 6 -1.717 <0.0001 Yes -2.064 -1.369

1 vs 5 -1.366 <0.0001 Yes -1.545 -1.188

1 vs 4 -0.708 <0.0001 Yes -0.843 -0.574

1 vs 3 -0.543 <0.0001 Yes -0.680 -0.406

1 vs 2 -0.131 0.013 No -0.286 0.024

2 vs 6 -1.586 <0.0001 Yes -1.933 -1.239

2 vs 5 -1.236 <0.0001 Yes -1.413 -1.059

2 vs 4 -0.578 <0.0001 Yes -0.711 -0.444

2 vs 3 -0.412 <0.0001 Yes -0.548 -0.276

3 vs 6 -1.174 <0.0001 Yes -1.513 -0.835

3 vs 5 -0.823 <0.0001 Yes -0.985 -0.661

3 vs 4 -0.165 <0.0001 Yes -0.278 -0.053

4 vs 6 -1.008 <0.0001 Yes -1.347 -0.670

4 vs 5 -0.658 <0.0001 Yes -0.818 -0.498

5 vs 6 -0.350 0.004 No -0.708 0.007

**Southern Africa. pregnant women age 15 to 20 years vs altitude range**

Contrast Difference P Significant Lo (95%) Up (95%)

1 vs 6 -2.086 0.015 No -4.600 0.428

1 vs 5 -0.835 0.048 No -2.081 0.410

1 vs 4 -0.541 0.149 No -1.650 0.569

1 vs 2 -0.474 0.239 No -1.666 0.719

1 vs 3 -0.132 0.708 No -1.182 0.917

3 vs 6 -1.954 0.019 No -4.399 0.491

3 vs 5 -0.703 0.059 No -1.802 0.397

3 vs 4 -0.408 0.199 No -1.351 0.534

3 vs 2 -0.341 0.330 No -1.380 0.697

2 vs 6 -1.613 0.058 No -4.122 0.897

2 vs 5 -0.362 0.386 No -1.598 0.875

2 vs 4 -0.067 0.856 No -1.166 1.032

4 vs 6 -1.545 0.065 No -4.016 0.926

4 vs 5 -0.295 0.450 No -1.451 0.862

5 vs 6 -1.251 0.144 No -3.786 1.284

**Southern Africa. pregnant women age > 20 years vs altitude range**

Contrast Difference P Significant Lo (95%) Up (95%)

2 vs 6 -1.180 0.000 Yes -2.109 -0.251

2 vs 5 -1.089 0.001 Yes -2.018 -0.160

2 vs 3 -0.632 0.046 No -1.561 0.297

2 vs 4 -0.621 0.049 No -1.550 0.308

2 vs 1 -0.370 0.241 No -1.299 0.559

1 vs 6 -0.810 0.010 No -1.739 0.119

1 vs 5 -0.719 0.023 No -1.648 0.210

1 vs 3 -0.262 0.407 No -1.191 0.667

1 vs 4 -0.251 0.427 No -1.180 0.678

4 vs 6 -0.559 0.077 No -1.488 0.370

4 vs 5 -0.468 0.138 No -1.397 0.461

4 vs 3 -0.011 0.972 No -0.940 0.918

3 vs 6 -0.548 0.083 No -1.477 0.381

3 vs 5 -0.457 0.148 No -1.386 0.472

5 vs 6 -0.091 0.773 No -1.020 0.838

**Eastern Africa. children 6 to 24 months vs. altitude range**

Contrast Difference P Significant Lo (95%) Up (95%)

1 vs 7 -2.036 <0.0001 Yes -2.569 -1.503

1 vs 6 -1.636 <0.0001 Yes -1.857 -1.415

1 vs 5 -1.536 <0.0001 Yes -1.646 -1.425

1 vs 4 -1.064 <0.0001 Yes -1.142 -0.986

1 vs 3 -0.593 <0.0001 Yes -0.661 -0.525

1 vs 2 -0.165 <0.0001 Yes -0.249 -0.080

2 vs 7 -1.871 <0.0001 Yes -2.406 -1.337

2 vs 6 -1.472 <0.0001 Yes -1.696 -1.247

2 vs 5 -1.371 <0.0001 Yes -1.488 -1.254

2 vs 4 -0.899 <0.0001 Yes -0.986 -0.813

2 vs 3 -0.428 <0.0001 Yes -0.507 -0.350

3 vs 7 -1.443 <0.0001 Yes -1.975 -0.911

3 vs 6 -1.043 <0.0001 Yes -1.262 -0.825

3 vs 5 -0.943 <0.0001 Yes -1.049 -0.837

3 vs 4 -0.471 <0.0001 Yes -0.542 -0.400

4 vs 7 -0.972 <0.0001 Yes -1.505 -0.438

4 vs 6 -0.572 <0.0001 Yes -0.794 -0.350

4 vs 5 -0.472 <0.0001 Yes -0.584 -0.360

5 vs 7 -0.500 0.005 No -1.039 0.039

5 vs 6 -0.100 0.195 No -0.336 0.135

6 vs 7 -0.400 0.034 No -0.972 0.173

**Eastern Africa. children 25 to 59 months vs. altitude range**

Contrast Difference P Significant Lo (95%) Up (95%)

1 vs 8 -3.073 <0.0001 Yes -3.930 -2.217

1 vs 7 -2.314 <0.0001 Yes -3.170 -1.457

1 vs 6 -1.716 <0.0001 Yes -2.573 -0.860

1 vs 5 -1.481 <0.0001 Yes -2.338 -0.625

1 vs 4 -1.025 0.000 Yes -1.881 -0.169

1 vs 3 -0.612 0.026 No -1.468 0.245

1 vs 2 -0.206 0.452 No -1.063 0.650

2 vs 8 -2.867 <0.0001 Yes -3.724 -2.011

2 vs 7 -2.107 <0.0001 Yes -2.964 -1.251

2 vs 6 -1.510 <0.0001 Yes -2.367 -0.654

2 vs 5 -1.275 <0.0001 Yes -2.132 -0.419

2 vs 4 -0.819 0.003 No -1.675 0.038

2 vs 3 -0.406 0.139 No -1.262 0.451

3 vs 8 -2.462 <0.0001 Yes -3.318 -1.605

3 vs 7 -1.702 <0.0001 Yes -2.558 -0.845

3 vs 6 -1.105 <0.0001 Yes -1.961 -0.248

3 vs 5 -0.870 0.002 Yes -1.726 -0.013

3 vs 4 -0.413 0.132 No -1.270 0.443

4 vs 8 -2.048 <0.0001 Yes -2.905 -1.192

4 vs 7 -1.289 <0.0001 Yes -2.145 -0.432

4 vs 6 -0.691 0.012 No -1.548 0.165

4 vs 5 -0.457 0.096 No -1.313 0.400

5 vs 8 -1.592 <0.0001 Yes -2.448 -0.736

5 vs 7 -0.832 0.002 No -1.688 0.024

5 vs 6 -0.235 0.391 No -1.091 0.621

6 vs 8 -1.357 <0.0001 Yes -2.213 -0.501

6 vs 7 -0.597 0.029 No -1.453 0.259

7 vs 8 -0.760 0.006 No -1.616 0.097

**Eastern Africa. not-pregnant women age 15 to 20 years vs altitude range**

Contrast Difference P Significant Lo (95%) Up (95%)

1 vs 7 -3.536 <0.0001 Yes -4.663 -2.408

1 vs 6 -3.056 <0.0001 Yes -4.184 -1.929

1 vs 5 -2.538 <0.0001 Yes -3.665 -1.410

1 vs 8 -2.497 <0.0001 Yes -3.625 -1.370

1 vs 4 -1.829 <0.0001 Yes -2.957 -0.702

1 vs 3 -1.073 0.003 No -2.200 0.055

1 vs 2 -0.709 0.050 No -1.836 0.419

2 vs 7 -2.827 <0.0001 Yes -3.955 -1.700

2 vs 6 -2.348 <0.0001 Yes -3.475 -1.220

2 vs 5 -1.829 <0.0001 Yes -2.957 -0.702

2 vs 8 -1.789 <0.0001 Yes -2.916 -0.661

2 vs 4 -1.121 0.002 No -2.248 0.007

2 vs 3 -0.364 0.313 No -1.491 0.763

3 vs 7 -2.463 <0.0001 Yes -3.591 -1.336

3 vs 6 -1.984 <0.0001 Yes -3.111 -0.856

3 vs 5 -1.465 <0.0001 Yes -2.593 -0.338

3 vs 8 -1.425 <0.0001 Yes -2.552 -0.297

3 vs 4 -0.756 0.036 No -1.884 0.371

4 vs 7 -1.707 <0.0001 Yes -2.834 -0.579

4 vs 6 -1.227 0.001 Yes -2.355 -0.100

4 vs 5 -0.709 0.050 No -1.836 0.419

4 vs 8 -0.668 0.064 No -1.796 0.459

8 vs 7 -1.038 0.004 No -2.166 0.089

8 vs 6 -0.559 0.121 No -1.686 0.569

8 vs 5 -0.041 0.911 No -1.168 1.087

5 vs 7 -0.998 0.006 No -2.125 0.130

5 vs 6 -0.518 0.151 No -1.646 0.609

6 vs 7 -0.480 0.184 No -1.607 0.648

**Eastern Africa. not-pregnant women age > 20 years vs altitude range**

Contrast Difference P Significant Lo (95%) Up (95%)

1 vs 8 -4.289 <0.0001 Yes -5.494 -3.085

1 vs 7 -3.149 <0.0001 Yes -3.484 -2.814

1 vs 6 -2.769 <0.0001 Yes -2.899 -2.640

1 vs 5 -2.416 <0.0001 Yes -2.485 -2.346

1 vs 4 -1.683 <0.0001 Yes -1.738 -1.627

1 vs 3 -1.058 <0.0001 Yes -1.108 -1.007

1 vs 2 -0.706 <0.0001 Yes -0.769 -0.644

2 vs 8 -3.583 <0.0001 Yes -4.788 -2.378

2 vs 7 -2.442 <0.0001 Yes -2.778 -2.106

2 vs 6 -2.063 <0.0001 Yes -2.195 -1.931

2 vs 5 -1.709 <0.0001 Yes -1.784 -1.635

2 vs 4 -0.976 <0.0001 Yes -1.038 -0.915

2 vs 3 -0.351 <0.0001 Yes -0.408 -0.295

3 vs 8 -3.232 <0.0001 Yes -4.436 -2.027

3 vs 7 -2.091 <0.0001 Yes -2.425 -1.757

3 vs 6 -1.712 <0.0001 Yes -1.839 -1.585

3 vs 5 -1.358 <0.0001 Yes -1.423 -1.293

3 vs 4 -0.625 <0.0001 Yes -0.674 -0.576

4 vs 8 -2.607 <0.0001 Yes -3.812 -1.402

4 vs 7 -1.466 <0.0001 Yes -1.801 -1.131

4 vs 6 -1.087 <0.0001 Yes -1.216 -0.958

4 vs 5 -0.733 <0.0001 Yes -0.802 -0.664

5 vs 8 -1.874 <0.0001 Yes -3.079 -0.668

5 vs 7 -0.733 <0.0001 Yes -1.070 -0.396

5 vs 6 -0.354 <0.0001 Yes -0.490 -0.218

6 vs 8 -1.520 <0.0001 Yes -2.731 -0.310

6 vs 7 -0.379 0.001 Yes -0.734 -0.025

7 vs 8 -1.141 0.004 No -2.390 0.109

**Eastern Africa. pregnant women age 15 to 20 years vs altitude range**

Contrast Difference P Significant Lo (95%) Up (95%)

1 vs 8 -6.979 <0.0001 Yes -10.412 -3.545

1 vs 5 -3.066 0.005 No -6.500 0.368

1 vs 6 -2.754 0.012 No -6.187 0.680

1 vs 4 -1.858 0.091 No -5.291 1.576

1 vs 7 -1.129 0.304 No -4.562 2.305

1 vs 3 -0.703 0.522 No -4.137 2.731

1 vs 2 -0.603 0.583 No -4.037 2.831

2 vs 8 -6.376 <0.0001 Yes -9.810 -2.942

2 vs 5 -2.463 0.025 No -5.897 0.971

2 vs 6 -2.151 0.050 No -5.585 1.283

2 vs 4 -1.255 0.253 No -4.688 2.179

2 vs 7 -0.526 0.632 No -3.960 2.908

2 vs 3 -0.100 0.928 No -3.534 3.334

3 vs 8 -6.276 <0.0001 Yes -9.710 -2.842

3 vs 5 -2.363 0.031 No -5.797 1.071

3 vs 6 -2.051 0.062 No -5.485 1.383

3 vs 4 -1.155 0.293 No -4.589 2.279

3 vs 7 -0.426 0.698 No -3.860 3.008

7 vs 8 -5.850 <0.0001 Yes -9.284 -2.416

7 vs 5 -1.937 0.078 No -5.371 1.497

7 vs 6 -1.625 0.139 No -5.059 1.809

7 vs 4 -0.729 0.507 No -4.163 2.705

4 vs 8 -5.121 <0.0001 Yes -8.555 -1.687

4 vs 5 -1.208 0.271 No -4.642 2.226

4 vs 6 -0.896 0.414 No -4.330 2.538

6 vs 8 -4.225 0.000 Yes -7.659 -0.791

6 vs 5 -0.312 0.776 No -3.746 3.122

5 vs 8 -3.913 0.000 Yes -7.347 -0.479

**Eastern Africa. pregnant women age > 20 years vs altitude range**

Contrast Difference P Significant Lo (95%) Up (95%)

1 vs 7 -2.906 <0.0001 Yes -4.813 -0.999

1 vs 6 -2.754 <0.0001 Yes -4.661 -0.847

1 vs 5 -2.379 <0.0001 Yes -4.286 -0.471

1 vs 8 -1.931 0.002 Yes -3.838 -0.024

1 vs 4 -1.619 0.008 No -3.526 0.289

1 vs 3 -0.857 0.160 No -2.765 1.050

1 vs 2 -0.561 0.358 No -2.468 1.346

2 vs 7 -2.345 0.000 Yes -4.253 -0.438

2 vs 6 -2.193 0.000 Yes -4.100 -0.286

2 vs 5 -1.818 0.003 No -3.725 0.090

2 vs 8 -1.370 0.025 No -3.278 0.537

2 vs 4 -1.058 0.083 No -2.965 0.850

2 vs 3 -0.296 0.627 No -2.204 1.611

3 vs 7 -2.049 0.001 Yes -3.956 -0.142

3 vs 6 -1.897 0.002 No -3.804 0.011

3 vs 5 -1.521 0.013 No -3.429 0.386

3 vs 8 -1.074 0.079 No -2.981 0.833

3 vs 4 -0.761 0.212 No -2.669 1.146

4 vs 7 -1.287 0.035 No -3.195 0.620

4 vs 6 -1.135 0.063 No -3.043 0.772

4 vs 5 -0.760 0.213 No -2.667 1.147

4 vs 8 -0.312 0.609 No -2.220 1.595

8 vs 7 -0.975 0.110 No -2.882 0.932

8 vs 6 -0.823 0.178 No -2.730 1.084

8 vs 5 -0.448 0.463 No -2.355 1.460

5 vs 7 -0.527 0.388 No -2.435 1.380

5 vs 6 -0.375 0.539 No -2.283 1.532

6 vs 7 -0.152 0.803 No -2.059 1.755

**Middle East. children 6 to 24 months vs. altitude range**

Contrast Difference P Significant Lo (95%) Up (95%)

4 vs 2 -1.524 0.015 No -3.419 0.371

4 vs 3 -0.998 0.110 No -2.893 0.897

4 vs 5 -0.536 0.390 No -2.431 1.359

4 vs 7 -0.488 0.434 No -2.383 1.407

4 vs 1 -0.426 0.494 No -2.321 1.469

4 vs 6 -0.348 0.577 No -2.243 1.547

6 vs 2 -1.176 0.059 No -3.071 0.719

6 vs 3 -0.650 0.297 No -2.545 1.245

6 vs 5 -0.188 0.763 No -2.083 1.707

6 vs 7 -0.140 0.822 No -2.036 1.755

6 vs 1 -0.078 0.900 No -1.974 1.817

1 vs 2 -1.098 0.078 No -2.993 0.797

1 vs 3 -0.571 0.359 No -2.466 1.324

1 vs 5 -0.110 0.860 No -2.005 1.785

1 vs 7 -0.062 0.921 No -1.957 1.833

7 vs 2 -1.036 0.097 No -2.931 0.859

7 vs 3 -0.509 0.414 No -2.404 1.386

7 vs 5 -0.048 0.939 No -1.943 1.847

5 vs 2 -0.988 0.113 No -2.883 0.907

5 vs 3 -0.461 0.459 No -2.357 1.434

3 vs 2 -0.526 0.398 No -2.422 1.369

**Middle East. children 25 to 59 months vs. altitude range**

Contrast Difference P Significant Lo (95%) Up (95%)

4 vs 2 -1.548 <0.0001 Yes -2.629 -0.467

4 vs 3 -0.892 0.012 No -1.973 0.189

4 vs 5 -0.393 0.269 No -1.474 0.688

4 vs 1 -0.362 0.309 No -1.443 0.719

4 vs 6 -0.352 0.323 No -1.433 0.729

4 vs 7 -0.146 0.681 No -1.227 0.935

7 vs 2 -1.402 <0.0001 Yes -2.483 -0.321

7 vs 3 -0.746 0.036 No -1.827 0.335

7 vs 5 -0.247 0.487 No -1.328 0.834

7 vs 1 -0.216 0.544 No -1.297 0.865

7 vs 6 -0.206 0.563 No -1.287 0.875

6 vs 2 -1.196 0.001 Yes -2.277 -0.115

6 vs 3 -0.540 0.129 No -1.621 0.541

6 vs 5 -0.041 0.907 No -1.122 1.040

6 vs 1 -0.010 0.977 No -1.091 1.071

1 vs 2 -1.186 0.001 Yes -2.267 -0.105

1 vs 3 -0.530 0.136 No -1.611 0.551

1 vs 5 -0.031 0.930 No -1.112 1.050

5 vs 2 -1.155 0.001 Yes -2.236 -0.074

5 vs 3 -0.499 0.161 No -1.580 0.582

3 vs 2 -0.656 0.065 No -1.737 0.425

**Middle East. not-pregnant women age 15 to 20 years vs altitude range**

Contrast Difference P Significant Lo (95%) Up (95%)

1 vs 3 -0.884 0.032 No -1.975 0.206

1 vs 4 -0.737 0.561 No -4.119 2.644

1 vs 2 -0.099 0.722 No -0.840 0.642

2 vs 3 -0.785 0.022 No -1.693 0.122

2 vs 4 -0.639 0.609 No -3.965 2.688

4 vs 3 -0.147 0.909 No -3.568 3.275

**Middle East. not-pregnant women age > 20 years vs altitude range**

Contrast Difference P Significant Lo (95%) Up (95%)

1 vs 4 -0.772 0.056 No -1.904 0.360

1 vs 3 -0.590 0.143 No -1.722 0.542

1 vs 2 -0.395 0.327 No -1.527 0.737

1 vs 5 -0.014 0.973 No -1.146 1.118

5 vs 4 -0.758 0.060 No -1.890 0.374

5 vs 3 -0.576 0.153 No -1.708 0.555

5 vs 2 -0.381 0.345 No -1.513 0.751

2 vs 4 -0.377 0.350 No -1.509 0.755

2 vs 3 -0.195 0.628 No -1.327 0.937

3 vs 4 -0.181 0.653 No -1.313 0.950

**Middle East. pregnant women age 15 to 20 years vs altitude range**

Contrast Difference P Significant Lo (95%) Up (95%)

2 vs 3 -0.514 0.322 No -1.781 0.753

2 vs 1 -0.125 0.787 No -1.262 1.012

1 vs 3 -0.389 0.547 No -1.969 1.191

**Middle East. pregnant women age > 20 years vs altitude range**

Contrast Difference P Significant Lo (95%) Up (95%)

1 vs 4 -1.168 0.018 No -2.557 0.222

1 vs 5 -1.131 0.022 No -2.520 0.258

1 vs 2 -0.388 0.432 No -1.778 1.001

1 vs 3 -0.371 0.453 No -1.760 1.018

3 vs 4 -0.797 0.107 No -2.186 0.593

3 vs 5 -0.760 0.124 No -2.149 0.629

3 vs 2 -0.017 0.972 No -1.407 1.372

2 vs 4 -0.779 0.115 No -2.168 0.610

2 vs 5 -0.743 0.133 No -2.132 0.646

5 vs 4 -0.036 0.941 No -1.426 1.353

**Central and West Asia. children 6 to 24 months vs. altitude range**

Contrast Difference P Significant Lo (95%) Up (95%)

1 vs 8 -1.589 <0.0001 Yes -2.630 -0.549

1 vs 7 -1.265 0.000 Yes -2.306 -0.224

1 vs 4 -0.584 0.080 No -1.624 0.457

1 vs 3 -0.444 0.183 No -1.484 0.597

1 vs 6 -0.408 0.220 No -1.449 0.633

1 vs 2 -0.359 0.280 No -1.400 0.681

1 vs 5 -0.263 0.429 No -1.304 0.777

5 vs 8 -1.326 <0.0001 Yes -2.367 -0.285

5 vs 7 -1.002 0.003 No -2.043 0.039

5 vs 4 -0.320 0.336 No -1.361 0.721

5 vs 3 -0.180 0.589 No -1.221 0.861

5 vs 6 -0.145 0.664 No -1.186 0.896

5 vs 2 -0.096 0.773 No -1.137 0.945

2 vs 8 -1.230 0.000 Yes -2.271 -0.189

2 vs 7 -0.906 0.007 No -1.947 0.135

2 vs 4 -0.224 0.501 No -1.265 0.817

2 vs 3 -0.084 0.801 No -1.125 0.957

2 vs 6 -0.049 0.884 No -1.090 0.992

6 vs 8 -1.181 0.000 Yes -2.222 -0.140

6 vs 7 -0.857 0.010 No -1.898 0.184

6 vs 4 -0.176 0.598 No -1.216 0.865

6 vs 3 -0.035 0.915 No -1.076 1.005

3 vs 8 -1.146 0.001 Yes -2.187 -0.105

3 vs 7 -0.822 0.014 No -1.862 0.219

3 vs 4 -0.140 0.674 No -1.181 0.901

4 vs 8 -1.006 0.003 No -2.046 0.035

4 vs 7 -0.681 0.041 No -1.722 0.359

7 vs 8 -0.324 0.330 No -1.365 0.717

**Central and West Asia. children 25 to 59 months vs. altitude range**

Contrast Difference P Significant Lo (95%) Up (95%)

1 vs 8 -1.758 <0.0001 Yes -2.402 -1.113

1 vs 7 -1.195 <0.0001 Yes -1.840 -0.551

1 vs 6 -0.735 0.000 Yes -1.379 -0.091

1 vs 4 -0.715 0.001 Yes -1.359 -0.070

1 vs 5 -0.664 0.001 Yes -1.308 -0.019

1 vs 3 -0.480 0.020 No -1.124 0.165

1 vs 2 -0.368 0.074 No -1.012 0.276

2 vs 8 -1.390 <0.0001 Yes -2.034 -0.745

2 vs 7 -0.827 <0.0001 Yes -1.472 -0.183

2 vs 6 -0.367 0.075 No -1.011 0.277

2 vs 4 -0.347 0.093 No -0.991 0.298

2 vs 5 -0.296 0.151 No -0.940 0.349

2 vs 3 -0.112 0.588 No -0.756 0.533

3 vs 8 -1.278 <0.0001 Yes -1.923 -0.634

3 vs 7 -0.716 0.001 Yes -1.360 -0.071

3 vs 6 -0.255 0.216 No -0.900 0.389

3 vs 4 -0.235 0.255 No -0.879 0.409

3 vs 5 -0.184 0.372 No -0.829 0.460

5 vs 8 -1.094 <0.0001 Yes -1.738 -0.450

5 vs 7 -0.532 0.010 No -1.176 0.113

5 vs 6 -0.071 0.730 No -0.716 0.573

5 vs 4 -0.051 0.805 No -0.695 0.594

4 vs 8 -1.043 <0.0001 Yes -1.688 -0.399

4 vs 7 -0.481 0.020 No -1.125 0.164

4 vs 6 -0.020 0.921 No -0.665 0.624

6 vs 8 -1.023 <0.0001 Yes -1.667 -0.378

6 vs 7 -0.460 0.026 No -1.105 0.184

7 vs 8 -0.562 0.006 No -1.207 0.082

**Central and West Asia. not-pregnant women age 15 to 20 years vs altitude range**

Contrast Difference P Significant Lo (95%) Up (95%)

1 vs 8 -2.372 <0.0001 Yes -3.216 -1.528

1 vs 6 -1.198 <0.0001 Yes -2.042 -0.354

1 vs 4 -1.152 <0.0001 Yes -1.996 -0.308

1 vs 7 -1.098 <0.0001 Yes -1.942 -0.255

1 vs 5 -1.065 <0.0001 Yes -1.909 -0.221

1 vs 3 -1.011 0.000 Yes -1.854 -0.167

1 vs 2 -0.661 0.014 No -1.505 0.182

2 vs 8 -1.710 <0.0001 Yes -2.554 -0.867

2 vs 6 -0.537 0.047 No -1.380 0.307

2 vs 4 -0.491 0.069 No -1.335 0.353

2 vs 7 -0.437 0.106 No -1.281 0.407

2 vs 5 -0.403 0.135 No -1.247 0.440

2 vs 3 -0.349 0.196 No -1.193 0.495

3 vs 8 -1.361 <0.0001 Yes -2.205 -0.517

3 vs 6 -0.187 0.487 No -1.031 0.656

3 vs 4 -0.142 0.600 No -0.985 0.702

3 vs 7 -0.088 0.745 No -0.932 0.756

3 vs 5 -0.054 0.841 No -0.898 0.790

5 vs 8 -1.307 <0.0001 Yes -2.151 -0.463

5 vs 6 -0.133 0.622 No -0.977 0.711

5 vs 4 -0.087 0.746 No -0.931 0.756

5 vs 7 -0.034 0.901 No -0.877 0.810

7 vs 8 -1.273 <0.0001 Yes -2.117 -0.430

7 vs 6 -0.100 0.712 No -0.943 0.744

7 vs 4 -0.054 0.842 No -0.898 0.790

4 vs 8 -1.220 <0.0001 Yes -2.063 -0.376

4 vs 6 -0.046 0.865 No -0.890 0.798

6 vs 8 -1.174 <0.0001 Yes -2.018 -0.330

**Central and West Asia. not-pregnant women age > 20 years vs altitude range**

Contrast Difference P Significant Lo (95%) Up (95%)

1 vs 8 -2.083 <0.0001 Yes -2.699 -1.468

1 vs 7 -1.818 <0.0001 Yes -2.310 -1.325

1 vs 6 -1.529 <0.0001 Yes -1.849 -1.210

1 vs 4 -1.304 <0.0001 Yes -1.427 -1.181

1 vs 5 -1.288 <0.0001 Yes -1.442 -1.133

1 vs 3 -1.125 <0.0001 Yes -1.242 -1.009

1 vs 2 -0.782 <0.0001 Yes -0.892 -0.672

2 vs 8 -1.301 <0.0001 Yes -1.910 -0.692

2 vs 7 -1.036 <0.0001 Yes -1.520 -0.551

2 vs 6 -0.747 <0.0001 Yes -1.054 -0.440

2 vs 4 -0.522 <0.0001 Yes -0.608 -0.435

2 vs 5 -0.506 <0.0001 Yes -0.633 -0.378

2 vs 3 -0.343 <0.0001 Yes -0.420 -0.266

3 vs 8 -0.958 <0.0001 Yes -1.568 -0.348

3 vs 7 -0.692 <0.0001 Yes -1.178 -0.207

3 vs 6 -0.404 <0.0001 Yes -0.713 -0.094

3 vs 4 -0.178 <0.0001 Yes -0.273 -0.084

3 vs 5 -0.162 0.000 Yes -0.295 -0.029

5 vs 8 -0.796 <0.0001 Yes -1.414 -0.177

5 vs 7 -0.530 0.001 Yes -1.027 -0.034

5 vs 6 -0.242 0.021 No -0.567 0.084

5 vs 4 -0.016 0.716 No -0.155 0.123

4 vs 8 -0.780 <0.0001 Yes -1.391 -0.168

4 vs 7 -0.514 0.001 Yes -1.001 -0.027

4 vs 6 -0.225 0.024 No -0.537 0.087

6 vs 8 -0.554 0.011 No -1.233 0.124

6 vs 7 -0.289 0.114 No -0.858 0.281

7 vs 8 -0.266 0.284 No -1.041 0.510

**Central and West Asia. pregnant women age 15 to 20 years vs altitude range**

Contrast Difference P Significant Lo (95%) Up (95%)

1 vs 2 -0.487 0.219 No -1.612 0.638

1 vs 3 -0.465 0.284 No -1.700 0.769

1 vs 4 -0.202 0.670 No -1.552 1.148

1 vs 5 -0.187 0.789 No -2.184 1.810

5 vs 2 -0.299 0.631 No -2.076 1.477

5 vs 3 -0.278 0.668 No -2.125 1.569

5 vs 4 -0.015 0.983 No -1.941 1.912

4 vs 2 -0.285 0.415 No -1.279 0.710

4 vs 3 -0.263 0.502 No -1.380 0.854

3 vs 2 -0.021 0.942 No -0.852 0.809

**Central and West Asia. pregnant women age > 20 years vs altitude range**

Contrast Difference P Significant Lo (95%) Up (95%)

1 vs 6 -2.238 0.004 No -4.692 0.216

1 vs 7 -1.468 0.061 No -3.922 0.986

1 vs 5 -1.240 0.114 No -3.694 1.214

1 vs 4 -1.044 0.183 No -3.498 1.409

1 vs 3 -0.838 0.285 No -3.292 1.616

1 vs 2 -0.710 0.365 No -3.164 1.743

1 vs 8 -0.693 0.377 No -3.147 1.761

8 vs 6 -1.545 0.049 No -3.999 0.909

8 vs 7 -0.775 0.323 No -3.229 1.679

8 vs 5 -0.547 0.486 No -3.000 1.907

8 vs 4 -0.351 0.654 No -2.805 2.103

8 vs 3 -0.145 0.853 No -2.599 2.309

8 vs 2 -0.017 0.982 No -2.471 2.436

2 vs 6 -1.528 0.052 No -3.981 0.926

2 vs 7 -0.758 0.334 No -3.211 1.696

2 vs 5 -0.529 0.500 No -2.983 1.924

2 vs 4 -0.334 0.670 No -2.788 2.120

2 vs 3 -0.128 0.871 No -2.581 2.326

3 vs 6 -1.400 0.074 No -3.854 1.054

3 vs 7 -0.630 0.422 No -3.084 1.824

3 vs 5 -0.402 0.609 No -2.855 2.052

3 vs 4 -0.206 0.793 No -2.660 2.248

4 vs 6 -1.194 0.128 No -3.648 1.260

4 vs 7 -0.424 0.589 No -2.878 2.030

4 vs 5 -0.195 0.803 No -2.649 2.258

5 vs 6 -0.998 0.203 No -3.452 1.455

5 vs 7 -0.228 0.771 No -2.682 2.225

7 vs 6 -0.770 0.326 No -3.224 1.684

**South/South-East Asia. children 6 to 24 months vs. altitude range**

Contrast Difference P Significant Lo (95%) Up (95%)

1 vs 11 -2.636 0.000 Yes -5.085 -0.188

1 vs 10 -2.136 0.004 No -4.585 0.312

1 vs 9 -1.315 0.075 No -3.763 1.133

1 vs 8 -0.964 0.192 No -3.412 1.485

1 vs 3 -0.846 0.252 No -3.294 1.602

1 vs 5 -0.789 0.285 No -3.238 1.659

1 vs 4 -0.764 0.301 No -3.212 1.684

1 vs 7 -0.569 0.441 No -3.018 1.879

1 vs 6 -0.437 0.554 No -2.885 2.011

1 vs 2 -0.279 0.705 No -2.728 2.169

2 vs 11 -2.357 0.001 No -4.805 0.091

2 vs 10 -1.857 0.012 No -4.305 0.591

2 vs 9 -1.036 0.160 No -3.484 1.413

2 vs 8 -0.684 0.354 No -3.133 1.764

2 vs 3 -0.567 0.443 No -3.015 1.882

2 vs 5 -0.510 0.490 No -2.958 1.938

2 vs 4 -0.485 0.511 No -2.933 1.964

2 vs 7 -0.290 0.694 No -2.738 2.158

2 vs 6 -0.158 0.831 No -2.606 2.291

6 vs 11 -2.199 0.003 No -4.647 0.249

6 vs 10 -1.699 0.021 No -4.147 0.749

6 vs 9 -0.878 0.234 No -3.326 1.570

6 vs 8 -0.527 0.476 No -2.975 1.922

6 vs 3 -0.409 0.580 No -2.857 2.039

6 vs 5 -0.352 0.633 No -2.800 2.096

6 vs 4 -0.327 0.658 No -2.775 2.121

6 vs 7 -0.132 0.858 No -2.580 2.316

7 vs 11 -2.067 0.005 No -4.515 0.381

7 vs 10 -1.567 0.034 No -4.015 0.881

7 vs 9 -0.746 0.312 No -3.194 1.702

7 vs 8 -0.394 0.593 No -2.843 2.054

7 vs 3 -0.277 0.708 No -2.725 2.172

7 vs 5 -0.220 0.766 No -2.668 2.228

7 vs 4 -0.195 0.792 No -2.643 2.254

4 vs 11 -1.872 0.011 No -4.321 0.576

4 vs 10 -1.372 0.063 No -3.821 1.076

4 vs 9 -0.551 0.455 No -2.999 1.897

4 vs 8 -0.200 0.787 No -2.648 2.249

4 vs 3 -0.082 0.912 No -2.530 2.366

4 vs 5 -0.025 0.973 No -2.474 2.423

5 vs 11 -1.847 0.012 No -4.295 0.601

5 vs 10 -1.347 0.068 No -3.795 1.101

5 vs 9 -0.526 0.476 No -2.974 1.922

5 vs 8 -0.174 0.813 No -2.623 2.274

5 vs 3 -0.057 0.939 No -2.505 2.392

3 vs 11 -1.790 0.015 No -4.239 0.658

3 vs 10 -1.290 0.080 No -3.739 1.158

3 vs 9 -0.469 0.525 No -2.918 1.979

3 vs 8 -0.118 0.873 No -2.566 2.331

8 vs 11 -1.673 0.023 No -4.121 0.776

8 vs 10 -1.173 0.112 No -3.621 1.276

8 vs 9 -0.351 0.634 No -2.800 2.097

9 vs 11 -1.321 0.073 No -3.769 1.127

9 vs 10 -0.821 0.266 No -3.269 1.627

10 vs 11 -0.500 0.498 No -2.948 1.948

**South/South-East Asia. children 25 to 59 months vs. altitude range**

Contrast Difference P Significant Lo (95%) Up (95%)

1 vs 11 -1.565 <0.0001 Yes -2.527 -0.603

1 vs 9 -1.317 <0.0001 Yes -2.279 -0.355

1 vs 10 -1.227 <0.0001 Yes -2.189 -0.265

1 vs 3 -0.961 0.001 No -1.924 0.001

1 vs 4 -0.822 0.005 No -1.784 0.140

1 vs 8 -0.803 0.006 No -1.765 0.159

1 vs 5 -0.712 0.014 No -1.674 0.250

1 vs 6 -0.650 0.025 No -1.612 0.312

1 vs 7 -0.585 0.044 No -1.547 0.377

1 vs 2 -0.359 0.216 No -1.321 0.603

2 vs 11 -1.206 <0.0001 Yes -2.168 -0.244

2 vs 9 -0.958 0.001 No -1.921 0.004

2 vs 10 -0.869 0.003 No -1.831 0.093

2 vs 3 -0.603 0.038 No -1.565 0.359

2 vs 4 -0.463 0.110 No -1.425 0.499

2 vs 8 -0.444 0.125 No -1.407 0.518

2 vs 5 -0.353 0.224 No -1.315 0.609

2 vs 6 -0.291 0.315 No -1.253 0.671

2 vs 7 -0.226 0.435 No -1.188 0.736

7 vs 11 -0.980 0.001 Yes -1.942 -0.018

7 vs 9 -0.732 0.012 No -1.694 0.230

7 vs 10 -0.642 0.027 No -1.604 0.320

7 vs 3 -0.376 0.194 No -1.338 0.586

7 vs 4 -0.237 0.414 No -1.199 0.725

7 vs 8 -0.218 0.452 No -1.180 0.744

7 vs 5 -0.127 0.662 No -1.089 0.835

7 vs 6 -0.065 0.823 No -1.027 0.897

6 vs 11 -0.915 0.002 No -1.877 0.047

6 vs 9 -0.667 0.021 No -1.629 0.295

6 vs 10 -0.577 0.046 No -1.540 0.385

6 vs 3 -0.311 0.283 No -1.274 0.651

6 vs 4 -0.172 0.553 No -1.134 0.790

6 vs 8 -0.153 0.597 No -1.115 0.809

6 vs 5 -0.062 0.831 No -1.024 0.900

5 vs 11 -0.853 0.003 No -1.815 0.109

5 vs 9 -0.605 0.037 No -1.568 0.357

5 vs 10 -0.516 0.075 No -1.478 0.446

5 vs 3 -0.250 0.389 No -1.212 0.712

5 vs 4 -0.110 0.704 No -1.072 0.852

5 vs 8 -0.091 0.752 No -1.054 0.871

8 vs 11 -0.762 0.009 No -1.724 0.200

8 vs 9 -0.514 0.076 No -1.476 0.448

8 vs 10 -0.424 0.144 No -1.386 0.538

8 vs 3 -0.158 0.585 No -1.120 0.804

8 vs 4 -0.019 0.948 No -0.981 0.943

4 vs 11 -0.743 0.010 No -1.705 0.219

4 vs 9 -0.495 0.088 No -1.457 0.467

4 vs 10 -0.405 0.162 No -1.367 0.557

4 vs 3 -0.139 0.631 No -1.101 0.823

3 vs 11 -0.603 0.037 No -1.566 0.359

3 vs 9 -0.356 0.220 No -1.318 0.606

3 vs 10 -0.266 0.359 No -1.228 0.696

10 vs 11 -0.338 0.245 No -1.300 0.625

10 vs 9 -0.090 0.757 No -1.052 0.872

9 vs 11 -0.248 0.393 No -1.210 0.714

**South/South-East Asia. not-pregnant women age 15 to 20 years vs altitude range**

Contrast Difference P Significant Lo (95%) Up (95%)

1 vs 11 -2.732 <0.0001 Yes -3.851 -1.613

1 vs 9 -1.534 <0.0001 Yes -2.653 -0.415

1 vs 10 -1.415 <0.0001 Yes -2.533 -0.296

1 vs 6 -1.061 0.002 No -2.180 0.058

1 vs 8 -1.051 0.002 No -2.170 0.068

1 vs 5 -0.906 0.007 No -2.025 0.213

1 vs 3 -0.904 0.007 No -2.023 0.215

1 vs 7 -0.839 0.013 No -1.958 0.280

1 vs 4 -0.746 0.027 No -1.865 0.373

1 vs 2 -0.336 0.319 No -1.455 0.783

2 vs 11 -2.396 <0.0001 Yes -3.515 -1.277

2 vs 9 -1.198 0.000 Yes -2.317 -0.079

2 vs 10 -1.078 0.001 No -2.197 0.040

2 vs 6 -0.725 0.032 No -1.844 0.394

2 vs 8 -0.715 0.034 No -1.834 0.404

2 vs 5 -0.570 0.091 No -1.689 0.549

2 vs 3 -0.568 0.092 No -1.687 0.551

2 vs 7 -0.503 0.136 No -1.622 0.616

2 vs 4 -0.410 0.225 No -1.529 0.709

4 vs 11 -1.987 <0.0001 Yes -3.106 -0.868

4 vs 9 -0.788 0.019 No -1.907 0.331

4 vs 10 -0.669 0.047 No -1.788 0.450

4 vs 6 -0.315 0.350 No -1.434 0.804

4 vs 8 -0.306 0.365 No -1.425 0.813

4 vs 5 -0.161 0.634 No -1.280 0.958

4 vs 3 -0.158 0.639 No -1.277 0.961

4 vs 7 -0.094 0.782 No -1.212 1.025

7 vs 11 -1.893 <0.0001 Yes -3.012 -0.774

7 vs 9 -0.695 0.039 No -1.814 0.424

7 vs 10 -0.575 0.088 No -1.694 0.544

7 vs 6 -0.222 0.511 No -1.341 0.897

7 vs 8 -0.212 0.529 No -1.331 0.907

7 vs 5 -0.067 0.842 No -1.186 1.052

7 vs 3 -0.065 0.848 No -1.184 1.054

3 vs 11 -1.828 <0.0001 Yes -2.947 -0.709

3 vs 9 -0.630 0.062 No -1.749 0.489

3 vs 10 -0.511 0.130 No -1.629 0.608

3 vs 6 -0.157 0.641 No -1.276 0.962

3 vs 8 -0.147 0.662 No -1.266 0.972

3 vs 5 -0.002 0.994 No -1.121 1.117

5 vs 11 -1.826 <0.0001 Yes -2.945 -0.707

5 vs 9 -0.628 0.063 No -1.747 0.491

5 vs 10 -0.508 0.132 No -1.627 0.611

5 vs 6 -0.155 0.646 No -1.274 0.964

5 vs 8 -0.145 0.667 No -1.264 0.974

8 vs 11 -1.681 <0.0001 Yes -2.800 -0.562

8 vs 9 -0.483 0.152 No -1.602 0.636

8 vs 10 -0.363 0.282 No -1.482 0.756

8 vs 6 -0.010 0.977 No -1.129 1.109

6 vs 11 -1.671 <0.0001 Yes -2.790 -0.552

6 vs 9 -0.473 0.161 No -1.592 0.646

6 vs 10 -0.353 0.295 No -1.472 0.765

10 vs 11 -1.318 <0.0001 Yes -2.437 -0.199

10 vs 9 -0.119 0.723 No -1.238 0.999

9 vs 11 -1.198 0.000 Yes -2.317 -0.079

**South/South-East Asia. not-pregnant women age > 20 years vs altitude range**

Contrast Difference P Significant Lo (95%) Up (95%)

1 vs 11 -2.777 <0.0001 Yes -3.214 -2.340

1 vs 10 -1.541 <0.0001 Yes -1.978 -1.104

1 vs 9 -1.454 <0.0001 Yes -1.891 -1.017

1 vs 6 -1.169 <0.0001 Yes -1.606 -0.732

1 vs 8 -1.157 <0.0001 Yes -1.594 -0.720

1 vs 7 -1.126 <0.0001 Yes -1.563 -0.689

1 vs 5 -1.045 <0.0001 Yes -1.482 -0.608

1 vs 3 -0.908 <0.0001 Yes -1.345 -0.471

1 vs 4 -0.772 <0.0001 Yes -1.209 -0.335

1 vs 2 -0.295 0.025 No -0.731 0.142

2 vs 11 -2.483 <0.0001 Yes -2.920 -2.046

2 vs 10 -1.246 <0.0001 Yes -1.683 -0.810

2 vs 9 -1.160 <0.0001 Yes -1.596 -0.723

2 vs 6 -0.874 <0.0001 Yes -1.311 -0.438

2 vs 8 -0.862 <0.0001 Yes -1.299 -0.426

2 vs 7 -0.831 <0.0001 Yes -1.268 -0.394

2 vs 5 -0.750 <0.0001 Yes -1.187 -0.313

2 vs 3 -0.613 <0.0001 Yes -1.050 -0.176

2 vs 4 -0.477 0.000 Yes -0.914 -0.040

4 vs 11 -2.005 <0.0001 Yes -2.442 -1.569

4 vs 10 -0.769 <0.0001 Yes -1.206 -0.332

4 vs 9 -0.682 <0.0001 Yes -1.119 -0.245

4 vs 6 -0.397 0.003 No -0.834 0.040

4 vs 8 -0.385 0.003 No -0.822 0.052

4 vs 7 -0.354 0.007 No -0.791 0.083

4 vs 5 -0.273 0.038 No -0.710 0.164

4 vs 3 -0.136 0.301 No -0.573 0.301

3 vs 11 -1.869 <0.0001 Yes -2.306 -1.433

3 vs 10 -0.633 <0.0001 Yes -1.070 -0.196

3 vs 9 -0.546 <0.0001 Yes -0.983 -0.109

3 vs 6 -0.261 0.047 No -0.698 0.176

3 vs 8 -0.249 0.059 No -0.686 0.188

3 vs 7 -0.218 0.098 No -0.655 0.219

3 vs 5 -0.137 0.298 No -0.574 0.300

5 vs 11 -1.732 <0.0001 Yes -2.169 -1.296

5 vs 10 -0.496 0.000 Yes -0.933 -0.059

5 vs 9 -0.409 0.002 No -0.846 0.028

5 vs 6 -0.124 0.345 No -0.561 0.313

5 vs 8 -0.112 0.395 No -0.549 0.325

5 vs 7 -0.081 0.539 No -0.518 0.356

7 vs 11 -1.651 <0.0001 Yes -2.088 -1.215

7 vs 10 -0.415 0.002 No -0.852 0.022

7 vs 9 -0.328 0.013 No -0.765 0.109

7 vs 6 -0.043 0.743 No -0.480 0.394

7 vs 8 -0.031 0.813 No -0.468 0.406

8 vs 11 -1.620 <0.0001 Yes -2.057 -1.183

8 vs 10 -0.384 0.004 No -0.821 0.053

8 vs 9 -0.297 0.024 No -0.734 0.140

8 vs 6 -0.012 0.927 No -0.449 0.425

6 vs 11 -1.608 <0.0001 Yes -2.045 -1.171

6 vs 10 -0.372 0.005 No -0.809 0.065

6 vs 9 -0.285 0.030 No -0.722 0.152

9 vs 11 -1.323 <0.0001 Yes -1.760 -0.886

9 vs 10 -0.087 0.509 No -0.524 0.350

10 vs 11 -1.236 <0.0001 Yes -1.673 -0.799

**South/South-East Asia. pregnant women age 15 to 20 years vs altitude range**

Contrast Difference P Significant Lo (95%) Up (95%)

1 vs 8 -2.250 0.030 No -5.491 0.991

1 vs 6 -2.200 0.034 No -5.441 1.041

1 vs 7 -1.650 0.112 No -4.891 1.591

1 vs 4 -1.080 0.298 No -4.321 2.161

1 vs 3 -0.695 0.503 No -3.936 2.546

1 vs 2 -0.228 0.826 No -3.469 3.014

1 vs 5 -0.094 0.928 No -3.335 3.148

5 vs 8 -2.157 0.038 No -5.398 1.085

5 vs 6 -2.107 0.042 No -5.348 1.135

5 vs 7 -1.557 0.133 No -4.798 1.685

5 vs 4 -0.987 0.341 No -4.228 2.255

5 vs 3 -0.601 0.562 No -3.843 2.640

5 vs 2 -0.134 0.897 No -3.375 3.107

2 vs 8 -2.022 0.051 No -5.264 1.219

2 vs 6 -1.972 0.057 No -5.214 1.269

2 vs 7 -1.422 0.170 No -4.664 1.819

2 vs 4 -0.852 0.411 No -4.094 2.389

2 vs 3 -0.467 0.652 No -3.709 2.774

3 vs 8 -1.555 0.134 No -4.796 1.686

3 vs 6 -1.505 0.147 No -4.746 1.736

3 vs 7 -0.955 0.357 No -4.196 2.286

3 vs 4 -0.385 0.710 No -3.626 2.856

4 vs 8 -1.170 0.259 No -4.411 2.071

4 vs 6 -1.120 0.280 No -4.361 2.121

4 vs 7 -0.570 0.583 No -3.811 2.671

7 vs 8 -0.600 0.563 No -3.841 2.641

7 vs 6 -0.550 0.596 No -3.791 2.691

6 vs 8 -0.050 0.962 No -3.291 3.191

**South/South-East Asia. pregnant women age > 20 years vs altitude range**

Contrast Difference P Significant Lo (95%) Up (95%)

1 vs 11 -2.999 <0.0001 Yes -5.099 -0.900

1 vs 10 -2.699 <0.0001 Yes -4.799 -0.600

1 vs 9 -1.490 0.019 No -3.589 0.610

1 vs 8 -1.291 0.041 No -3.391 0.808

1 vs 6 -1.121 0.076 No -3.220 0.978

1 vs 5 -1.045 0.099 No -3.144 1.055

1 vs 7 -0.829 0.190 No -2.929 1.270

1 vs 4 -0.751 0.236 No -2.850 1.349

1 vs 3 -0.734 0.246 No -2.833 1.366

1 vs 2 -0.304 0.631 No -2.403 1.796

2 vs 11 -2.696 <0.0001 Yes -4.795 -0.596

2 vs 10 -2.396 0.000 Yes -4.495 -0.296

2 vs 9 -1.186 0.061 No -3.285 0.913

2 vs 8 -0.988 0.119 No -3.087 1.112

2 vs 6 -0.817 0.196 No -2.917 1.282

2 vs 5 -0.741 0.242 No -2.840 1.359

2 vs 7 -0.526 0.406 No -2.625 1.574

2 vs 4 -0.447 0.480 No -2.546 1.653

2 vs 3 -0.430 0.497 No -2.530 1.669

3 vs 11 -2.265 0.000 Yes -4.365 -0.166

3 vs 10 -1.965 0.002 No -4.065 0.134

3 vs 9 -0.756 0.232 No -2.855 1.343

3 vs 8 -0.558 0.378 No -2.657 1.542

3 vs 6 -0.387 0.541 No -2.487 1.712

3 vs 5 -0.311 0.623 No -2.410 1.789

3 vs 7 -0.095 0.880 No -2.195 2.004

3 vs 4 -0.017 0.979 No -2.116 2.083

4 vs 11 -2.249 0.000 Yes -4.348 -0.149

4 vs 10 -1.949 0.002 No -4.048 0.151

4 vs 9 -0.739 0.243 No -2.839 1.360

4 vs 8 -0.541 0.393 No -2.640 1.559

4 vs 6 -0.371 0.558 No -2.470 1.729

4 vs 5 -0.294 0.642 No -2.393 1.805

4 vs 7 -0.079 0.901 No -2.178 2.021

7 vs 11 -2.170 0.001 Yes -4.269 -0.071

7 vs 10 -1.870 0.003 No -3.969 0.229

7 vs 9 -0.661 0.297 No -2.760 1.439

7 vs 8 -0.462 0.465 No -2.562 1.637

7 vs 6 -0.292 0.645 No -2.391 1.808

7 vs 5 -0.215 0.734 No -2.315 1.884

5 vs 11 -1.955 0.002 No -4.054 0.145

5 vs 10 -1.655 0.009 No -3.754 0.445

5 vs 9 -0.445 0.482 No -2.545 1.654

5 vs 8 -0.247 0.696 No -2.346 1.852

5 vs 6 -0.077 0.904 No -2.176 2.023

6 vs 11 -1.878 0.003 No -3.978 0.221

6 vs 10 -1.578 0.013 No -3.678 0.521

6 vs 9 -0.369 0.560 No -2.468 1.731

6 vs 8 -0.170 0.788 No -2.270 1.929

8 vs 11 -1.708 0.007 No -3.807 0.392

8 vs 10 -1.408 0.026 No -3.507 0.692

8 vs 9 -0.198 0.754 No -2.298 1.901

9 vs 11 -1.510 0.017 No -3.609 0.590

9 vs 10 -1.210 0.056 No -3.309 0.890

10 vs 11 -0.300 0.635 No -2.399 1.799

**Table S8. P-values of multiple comparisons of the intercepts and slopes of the regressions between age- and pregnancy subgroups within a region.** Intercepts and slopes of the regressions are shown in **table 2** of the main manuscript. A P-value of “0” indicates a P<0.0005. Italic print for P-values >0.05. Int. … intercept (sea-level [Hb]), slope … delta [Hb]/km of altitude, mo ... months, y ... years, np ... not pregnant, pr ... pregnant.

**Central America**

**6-24 mo 25-59 mo 15-20 y np >20 y np 15-20 y pr >20y pr**

**Int. Slope Int. Slope Int. Slope Int. Slope Int. Slope Int. Slope**

**6-24 mo** 0 0 0 0 0 0 0 0 0 0

**24-59 mo** 0 0 0 0 0 0 0.001 0.025 0 0.006

**15-20 y np** 0 0 0 0 *0.191* 0 0 *0.300* 0 0.005

**>20 y np** 0 0 0 0 *0.191* 0 0 *0.649* 0 *0.718*

**15-20 y pr** 0 0 0.001 0.025 0 *0.300* 0 *0.649* *0.676* *0.581*

**>20 y pr** 0 0 0 0.006 0 0.005 0 *0.718* *0.676* *0.581*

**South America**

**6-24 mo 25-59 mo 15-20 y np >20 y np 15-20 y pr >20y pr**

**Int. Slope Int. Slope Int. Slope Int. Slope Int. Slope Int. Slope**

**6-24 mo** 0 0.027 0 0 0 0 0 0 0 0

**24-59 mo** 0 0.027 0 0 0 0 0.009 0 0.021 0.008

**15-20 y np** 0 0 0 0 0.002 0 0 *0.725* 0 0

**>20 y np** 0 0 0 0 0.002 0 0 *0.246* 0 0.002

**15-20 y pr** 0 0 0.009 0 0 *0.725* 0 *0.246* 0.001 0.018

**>20 y pr** 0 0 0.021 0.008 0 0 0 0.002 0.001 0.018

**West and Central Africa**

**6-24 mo 25-59 mo 15-20 y np >20 y np 15-20 y pr >20y pr**

**Int. Slope Int. Slope Int. Slope Int. Slope Int. Slope Int. Slope**

**6-24 mo** 0 *0.237* 0 0 0 0.036 0 *0.764* 0 0.018

**24-59 mo** 0 *0.237* 0 0 0 *0.254* *0.226* *0.965* 0 *0.070*

**15-20 y np** 0 0 0 0 *0.084* 0.003 0 *0.272* 0 *0.431*

**>20 y np** 0 0.036 0 *0.254* *0.084* 0.003 0 *0.829* 0 *0.213*

**15-20 y pr** 0 *0.764* *0.226* *0.965* 0 *0.272* 0 *0.829* *0.326* *0.484*

**>20 y pr** 0 0.018 0 *0.070* 0 *0.431* 0 *0.213* *0.326* *0.484*

**Southern Africa**

**6-24 mo 25-59 mo 15-20 y np >20 y np 15-20 y pr >20y pr**

**Int. Slope Int. Slope Int. Slope Int. Slope Int. Slope Int. Slope**

**6-24 mo** 0 0 0 0.018 0 *0.139* 0 *0.633* 0 *0.079*

**24-59 mo** 0 0 0 0 0 0 *0.059* *0.480* 0.003 *0.788*

**15-20 y np** 0 0.018 0 0 *0.513* *0.119* 0.008 *0.188* 0 0.001

**>20 y np** 0 *0.139* 0 0 0.52 *0.119* 0.004 *0.347* 0 0.006

**15-20 y pr** 0 *0.633* *0.059* *0.480* 0.009 *0.188* 0.004 *0.347* *0.781* *0.618*

**>20 y pr** 0 *0.079* 0.003 *0.788* 0 0.001 0 0.006 *0.781* *0.618*

**Eastern Africa**

**6-24 mo 25-59 mo 15-20 y np >20 y np 15-20 y pr >20y pr**

**Int. Slope Int. Slope Int. Slope Int. Slope Int. Slope Int. Slope**

**6-24 mo** 0 *0.525* 0 0 0 0 0.001 0 0 0

**24-59 mo** 0 *0.525* 0 0 0 0 0 0 0 0

**15-20 y np** 0 0 0 0 0.026 0 0 *0.679* 0 0

**>20 y np** 0 0 0 0 0.026 0 0 *0.449* 0 *0.394*

**15-20 y pr** 0.001 0 0 0 0 *0.679* 0 *0.449* 0 *0.304*

**>20 y pr** 0 0 0 0 0 0 0 *0.394* 0 *0.304*

**Middle East**

**6-24 mo 25-59 mo 15-20 y np >20 y np 15-20 ypr >20y pr**

**Int. Slope Int. Slope Int. Slope Int. Slope Int. Slope Int. Slope**

**6-24 mo** 0 *0.508* 0 0.043 0 0 *0.514 0.109* 0 0

**24-59 mo** 0 *0.508* 0.008 0.034 0 0 *0.515 0.100* 0.007 0

**15-20 y np** 0 0.043 0.008 0.034 *0.518* *0.908* *0.072 0.627* 0.001 *0.763*

**>20 y np** 0 0 0 0 *0.518 0.908* *0.092 0.545* 0 *0.555*

**15-20 y pr** *0.514* *0.103* *0.515* *0.100* *0.072 0.627* *0.092* *0.545* *0.892 0.483*

**>20 y pr** 0 0 0.007 0 0.001 *0.763* 0 *0.555* *0.892 0.483*

**Central and Western Asia**

**6-24 mo 25-59 mo 15-20 y np >20 y np 15-20 y pr >20y pr**

**Int. Slope Int. Slope Int. Slope Int. Slope Int. Slope Int. Slope**

**6-24 mo** 0 0.021 0 0 0 0 0.021 *0.473* 0.001 0

**24-59 mo** 0 0.021 0 0.001 0 0 *0.894* *0.270* 0 0.004

**15-20 y np** 0 0 0 0 0 0.034 0.005 *0.086* 0 *0.575*

**>20 y np** 0 0 0 0 0 0.034 0.025 0.039 0 *0.532*

**15-20 y pr** 0.021 *0.473* *0.894 0.270* 0.005 *0.0869* 0.025 0.039 *0.223 0.066*

**>20 y pr** 0.001 0 0 0.004 0 *0.575* 0 *0.532* *0.223* *0.066*

**South and South-Eastern Asia**

**6-24 mo 25-59 mo 15-20 y np >20 y np 15-20 y pr >20y pr**

**Int. Slope Int. Slope Int. Slope Int. Slope Int. Slope Int. Slope**

**6-24 mo** 0 0.001 0 0 0 0 0 0.019 0 0.007

**24-59 mo** 0 0.001 0 0 0 0 *0.108 0.126* 0 *0.633*

**15-20 y np** 0 0 0 0 0.008 *0.787* 0 *0.679* 0 0.004

**>20 y np** 0 0 0 0 0.008 *0.787* 0 *0.644* 0 0.003

**15-20 y pr** 0 0.019 *0.108* *0.126* 0 *0.679* 0 *0.644* *0.204 0.185*

**>20 y pr** 0 0.007 0 *0.633* 0 0.004 0 0.003 *0.204 0.185*

**Table S9. P-values of multiple comparisons of the intercepts and slopes of the regressions between regions within age- and pregnancy subgroups.** Intercepts and slopes of the regressions are shown in **table 2** of the main manuscript. A P-value of “0” indicates a P<0.0005. Italic print for P-values >0.05. Int. … intercept (sea-level [Hb]), slope … delta [Hb]/km of altitude, mo ... months, y ... years, np ... not pregnant, pr ... pregnant

**Children age 6 to 24 months**

**SSEA EAfr CAm SAm CWA ME WCAfr SAfr**

**Int. Slope Int. Slope Int. Slope Int. Slope Int. Slope Int. Slope Int. Slope Int. Slope**

**SSEA** 0 0 0 0.001 0 0 0 0 0 0 0 0 *0.330* 0.008

**EAfr** 0 0 0 0 0 0 0 0 0 0 0 *0.186* 0 0.002

**CAm** 0 *0.122* 0 0 0 0 *0.279* 0.003 *0.512* 0 0 0 0 0.001

**SAm** 0 0 0 0 0 0 0 0 0 0 0 0.044 0 *0.345*

**CWA** 0 0 0 0 *0.279* 0.003 0 0 *0.714* 0 0 0 0 0

**ME** 0 0 0 0 *0.512* 0 0 0 *0.714* 0 0 0 0 0

**WCAfr** 0 0 0 *0.186* 0 0 0 0.044 0 0 0 0 0 0.046

**SAfr** *0.330* 0.008 0 0.002 0 0.011 0 *0.345* 0 0 0 0 0 0.046

**Children age 24 to 59 months**

**SSEA EAfr CAm SAm CWA ME WCAfr SAfr**

**Int. Slope Int. Slope Int. Slope Int. Slope Int. Slope Int. Slope Int. Slope Int. Slope**

**SSEA** 0 0 0 0.117 0 0 0 0 0 0 0 0 0 0

**EAfr** 0 0 0 0 0 0 0 0 0 0 0 *0.530* 0 0

**CAm** 0 0.012 0 0 0.029 0 0.003 0 *0.858* 0 0 0 0 0

**SAm** 0 0 0 0 0.029 0 0 0 *0.077* 0 0 0 0 0

**CWA** 0 0 0 0 0.003 0 0 0 0.023 0 0 0 0 *0.378*

**ME** 0 0 0 0 *0.856* 0 *0.077* 0 0.023 0 0 0 0 0

**WCAfr** 0 0 0 *0.530* 0 0 0 0 0 0 0 0 0 0

**SAfr** 0 0 0 0 0 0 0 0 0 *0.378* 0 0 0 0

**Females 15 to 20 years, not pregnant**

**SSEA EAfr CAm SAm CWA ME WCAfr SAfr**

**Int. Slope Int. Slope Int. Slope Int. Slope Int. Slope Int. Slope Int. Slope Int. Slope**

**SSEA** 0 0 0 0 0 0 0 *0.754* 0.026 *0.965* *0.754* 0 0 0

**EAfr** 0 0 0 0 0 0 0 0 0.008 *0.085* 0.001 0 0 0

**CAm** 0 0 0 0 0 0.001 0 0 0.025 *0.647* 0 0.001 0 *0.610*

**SAm** 0 0 0 0 0 0.001 0 0 0.110 *0.524* 0 0.042 0 0.026

**CWA** 0 *0.754* 0 0 0 0 0 0 *0.997* *0.938* 0 0 *0.246* 0.001

**ME** 0.026 *0.965* 0.008 *0.085* 0.025 *0.647* *0.110* *0.524* *0.997* *0.938* 0.030 *0.389* *0.763 0.691*

**WCAfr** *0.754* 0 0.001 0 0 0.001 0 0.042 0 0 0.030 *0.389* 0 0.002

**SAfr** 0 0 0 0 0 *0.61* 0 *0.026* *0.246* 0.001 *0.763 0.691* 0 0.002

**Females > 20 years, not pregnant**

**SSEA EAfr CAm SAm CWA ME WCAfr SAfr**

**Int. Slope Int. Slope Int. Slope Int. Slope Int. Slope Int. Slope Int. Slope Int. Slope**

**SSEA** 0 0 0 0 0 0 0 0 0 *0.519* 0.001 0 0 0

**EAfr** 0 0 0 0 0 0 0 0 0 0 0 0 0 0

**CAm** 0 0 0 0 0 0 0 *0.571* 0 0.009 0 0 0 *0.969*

**SAm** 0 0 0 0 0 0 0 0 0 0 0 *0.452* 0 0

**CWA** 0 0 0 0 0 *0.571* 0 0 *0.807* 0.026 0 0 0 *0.648*

**ME** 0 *0.519* 0 0 0 0.009 0 0 *0.807* 0.026 0 0 0 0.014

**WCAfr** 0.001 0 0 0 0 0 0 *0.452* 0 0 0 0 0 0.001

**SAfr** 0 0 0 0 0 *0.969* 0 0 0 *0.648* 0 0.014 0 0.001

**Females 15-20 years. pregnant**

**SSEA EAfr CAm SAm CWA ME WCAfr SAfr**

**Int. Slope Int. Slope Int. Slope Int. Slope Int. Slope Int. Slope Int. Slope Int. Slope**

**SSEA** 0 0 0 *0.347* 0 0.004 *0.054 0.092 0.595 0.575* 0.005 *0.421* 0.001 *0.562*

**EAfr** 0 0 0 0 0 0 0 0 *0.071 0.822* 0.010 0.007 0 0

**CAm** 0 *0.347* 0 0 0.002 *0.109* *0.403* 0.048 *0.278 0.661* 0 *0.803* *0.864 0.296*

**SAm** 0 0.004 0 0 0.002 *0.109* *0.674* 0.012 *0.741 0.810* 0 *0.517* *0.167 0.058*

**CWA** *0.054 0.092* 0 0 *0.403* 0.048 *0.674* 0.012 *0.607 0.251* 0.005 *0.054* *0.567 0.247*

**ME** *0.595 0.575* 0.071 *0.822* *0.278* *0.661* *0.741 0.810* *0.607 0.251* *0.256 0.707* *0.354 0.491*

**WCAfr** 0.005 *0.421* 0.01 0.007 0 *0.803* 0 *0.517* 0.005 *0.054* *0.256 0.707* 0 *0.300*

**SAfr** 0.001 *0.562* 0 0 *0.864* *0.296* *0.167 0.058* *0.567 0.247* *0.354 0.491* 0 *0.300*

**Females >20 years. pregnant**

**SSEA EAfr CAm SAm CWA ME WCAfr SAfr**

**Int. Slope Int. Slope Int. Slope Int. Slope Int. Slope Int. Slope Int. Slope Int. Slope**

**SSEA** 0 0 0 0.004 0 0 0.022 *0.247* 0.001 *0.658* 0 0 0 *0.183*

**EAfr** 0 0 0 0 0 0 0 0 0 0 0.002 0 0 0

**CAm** 0 0.004 0 0 *0.665* *0.165* 0 *0.614* 0 *0.151* 0 0.008 *0.495* 0.011

**SAm** 0 0 0 0 *0.665* *0.165* 0 *0.159* 0 0.043 0 *0.051* *0.648* 0.001

**CWA** 0.022 *0.247* 0 0 0 *0.614* 0 *0.159* *0.356* *0.317* 0 0.013 0.001 *0.071*

**ME** 0.001 *0.658* 0 0 0 *0.151* 0 0.043 *0.356* *0.317* 0 0.005 0.014 *0.642*

**WCAfr** 0 0 0.002 0 0 0.008 0 *0.051* 0 0.013 0 0.005 0 0

**SAfr** 0 *0.183* 0 0 *0.495* 0.011 *0.648* 0.001 0.001 *0.071* 0.014 *0.642* 0 0

**Supplementary references**

1. Sullivan KM. Mei Z. Grummer-Strawn L. Parvanta I. Haemoglobin adjustments to define anaemia. *Trop Med Int Health*. Oct 2008;13(10):1267-71. doi:10.1111/j.1365-3156.2008.02143.x

2. Nestel P. Adjusting hemoglobin values for program surveys. *Internatioanl Nutritional Anemia Consultative Group (INACG)*. 2002;

3. WHO. Haemoglobin concentration for the diagnosis of anaemia and assessment of severity. Vitamin and Mineral Nutrition Information System. Geneva. World Health Organization. 2011 (WHO/NMH/NHD/MNM/11.1) (<http://www.who.int/vmnis/indicators/haemoglobin.pdf>); (accessed last Nov. 25. 2020).

4. Gassmann M. Mairbäurl H. Livshits L. et al. The increase in hemoglobin concentration with altitude varies among human populations. *Ann N Y Acad Sci*. Jun 30 2019;1450:204-220. doi:10.1111/nyas.14136

5. Fulwood R. Johnson CL. Bryner JD. Gunter EW. McGrath CR. Hematological and nutritional biochemistry data for persons 6 months - 74 years of age: United States. 1976 - 80. *DHHS Publication No (PHS) 83-1682*. 1982:1-183.

6. Accinelli RA. Leon-Abarca JA. Age and altitude of residence determine anemia prevalence in Peruvian 6 to 35 months old children. *PloS one*. 2020;15(1):e0226846. doi:10.1371/journal.pone.0226846

7. Ocas-Cordova S. Tapia V. Gonzales GF. Hemoglobin Concentration in Children at Different Altitudes in Peru: Proposal for [Hb] Correction for Altitude to Diagnose Anemia and Polycythemia. *High altitude medicine & biology*. Dec 2018;19(4):398-403. doi:10.1089/ham.2018.0032
